# Controlled Polymerization Catalysis for the Synthesis of Degradable Amphiphilic Polycarbonates from CO_2_

**DOI:** 10.1021/jacs.5c20433

**Published:** 2026-02-12

**Authors:** Diego A. Resendiz-Lara, Thomas Habets, Steven P. Armes, Charlotte K. Williams

**Affiliations:** † Chemistry Research Laboratory, Department of Chemistry, 6396 University of Oxford, 12 Mansfield Road, Oxford OX1 3TA, U.K.; ‡ School of Mathematics and Physical Sciences, 7315 University of Sheffield, Dainton Building, Brook Hill, Sheffield, South Yorkshire S3 7HF, U.K.

## Abstract

Synthetic water-soluble polymers are ubiquitous in solution-based applications, but their petroleum origin and nondegradable bonds create environmental concerns. Here, CO_2_- and glycerol-derived polycarbonates incorporating hydrophilic diglycerol motifs are prepared as a general-purpose water-soluble degradable polymer platform. A high-performance heterodinuclear [Co­(III)/K­(I)] catalyst enables controlled ring-opening copolymerization (ROCOP) of CO_2_ with an acetal-protected epoxide, delivering well-defined polycarbonates with low dispersity (*D̵* < 1.2) and predictable molecular weights (≈2000–20,000 g mol^–1^). The catalysis is tolerant to protic initiators (chain transfer agents, CTAs), enabling control over both chain length and end-group chemistry. Deprotection of the acetals is quantitative and affords water-soluble polycarbonates incorporating hydrophilic diglycerol motifs. Using natural hydrophobic initiators yields amphiphilic polymers that self-assemble in water to form nanostructures of ≈7–11 nm with a critical micelle concentration of ≈30 mg L^–1^. These polymers are stable at either neutral or acidic pH but depolymerize in alkaline solution to form nontoxic small molecules. Degradation proceeds by hydroxyl chain-end–initiated backbiting, i.e. by self-immolation, with pH- and end-cap-dependent kinetics, with complete degradation occurring over minutes to one month. Overall, this renewable polycarbonate chemistry, which is ∼23 wt % CO_2_-derived; ∼77 wt % glycerol-derived, combines precise polymerization catalysis, spontaneous aqueous self-assembly and controllable aqueous degradability which are important for next-generation surfactants.

## Graphical abstract

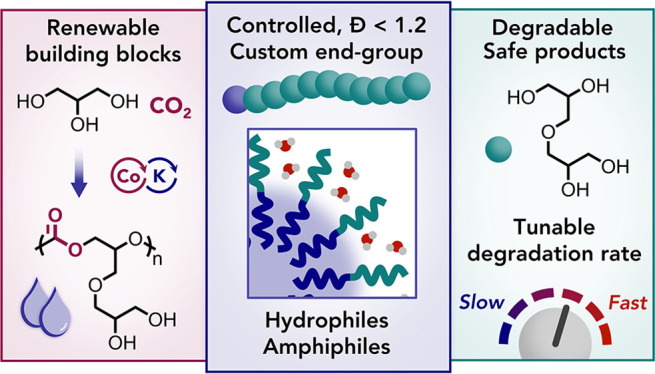

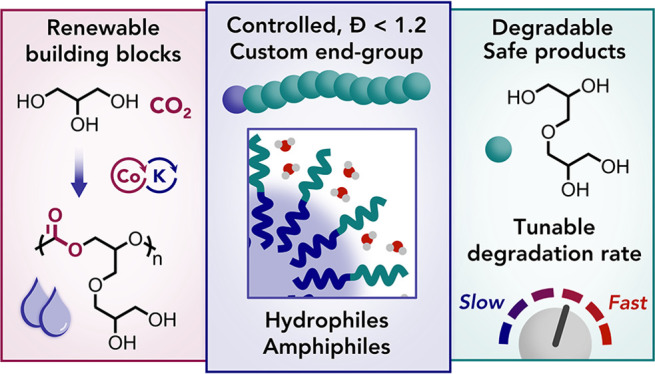

## Introduction

Using carbon dioxide as a C_1_-source in chemistry is attractive as it is abundant, inexpensive, of low toxicity and a common waste product of many industrial and biochemical processes.^1,2^ Making polymers from carbon dioxide is particularly important since they are among the largest volume chemicals produced.^3,4^ Delivering more sustainable polymer manufacturing requires severe limits to the consumption of virgin petrochemical feedstocks so as to stop the ever-growing greenhouse gas emissions arising from oil refining and monomer production.^3^ Since lower product embedded emissions generally correlate with simpler, energy efficient manufacturing processes, attention has focused on the direct use of carbon dioxide as a monomer.^3,5–9^ Accordingly, the ring-opening copolymerization (ROCOP) of CO_2_ with epoxides is a promising carbon dioxide utilization producing polycarbonates showing genuinely lower embedded emissions (vs petrochemical analogues) and comprising up to 50 wt % CO_2_.^3,5–7^ Successful CO_2_/epoxide ROCOP requires a catalyst, with the best ones showing excellent rates, productivity, selectivity and control.^10–13^ Controlled polymerization catalysts must successfully tolerate the addition of chain transfer agents (CTAs) which are protic compounds such as water or diols.^14^ These protic compounds undergo rapid and reversible exchange with the propagating chain and enable control over the polycarbonate chain length, end group chemistry and architecture.^14^ The development of high-performance catalysts that copolymerize a range of epoxides has motivated research into the resulting polycarbonate material properties and application potential;^15–19^ promising performances are already demonstrated as polyols for polyurethane production, engineering thermoplastics, thermoplastic elastomers, adhesives and ion-conducting electrolytes.^5,19,20^

There is also a need to reconsider the design of polymers for aqueous or liquid formulations (PLFs) so as to ensure properties are compatible with the wide-range of current applications while tackling growing concerns associated with their end-life and wastes.^21–24^ These polymer formulations are produced at very large scale, ∼36 million tonnes/annum, and applied in agriculture, medicine, cleaning, personal care, paints, coatings and many more.^25^ Their solution behavior depends upon the polymer structure and for some chemistries it was now quite well understood how to tailor polymer structure for desired application performance.^26–33^ Amphiphilic polymer surfactants are particularly widely applied and comprise hydrophilic and hydrophobic segments that undergo aqueous self-assembly useful for the controlled delivery and/or release of other active molecules, e.g. pharmaceuticals or fragrances. One challenge is that the highly dilute aqueous polymer solutions typically end up being dispersed in wastewater or soil after use; unlike other polymers they cannot be collected for reuse or recycling.^3^ To address environmental concerns, new design criteria for these polymers are emerging: (i) maximize renewable carbon uptake in the polymers, by using biobased and/or CO_2_-derived monomers and (ii) deliver polymers that undergo aqueous (bio)­degradation after use. Currently polyacrylates and -acrylamides show really impressive performances and by using controlled radical polymerization synthetic methods, outstanding selectivity for target chain lengths, architectures, and comonomer compositions can be achieved, but their hydrocarbon backbones prevent (bio)­degradation (Scheme 1A).^22,34–36^ Although recent copolymerization strategies have demonstrated methods to introduce hydrolytically cleavable units into the polyacrylate/acrylamide backbones, the degradable chemistry is often part of the hydrophobic domain and may result in the release of nondegradable polymer segments.^32,37,38^ CO_2_–polycarbonates may be able to offer comparable structural tuneability to these vinyl-derived polymers while the regular carbonate repeat unit chemistries both maximize carbon dioxide use and provide sites for chain degradation after use.

**Scheme 1:**
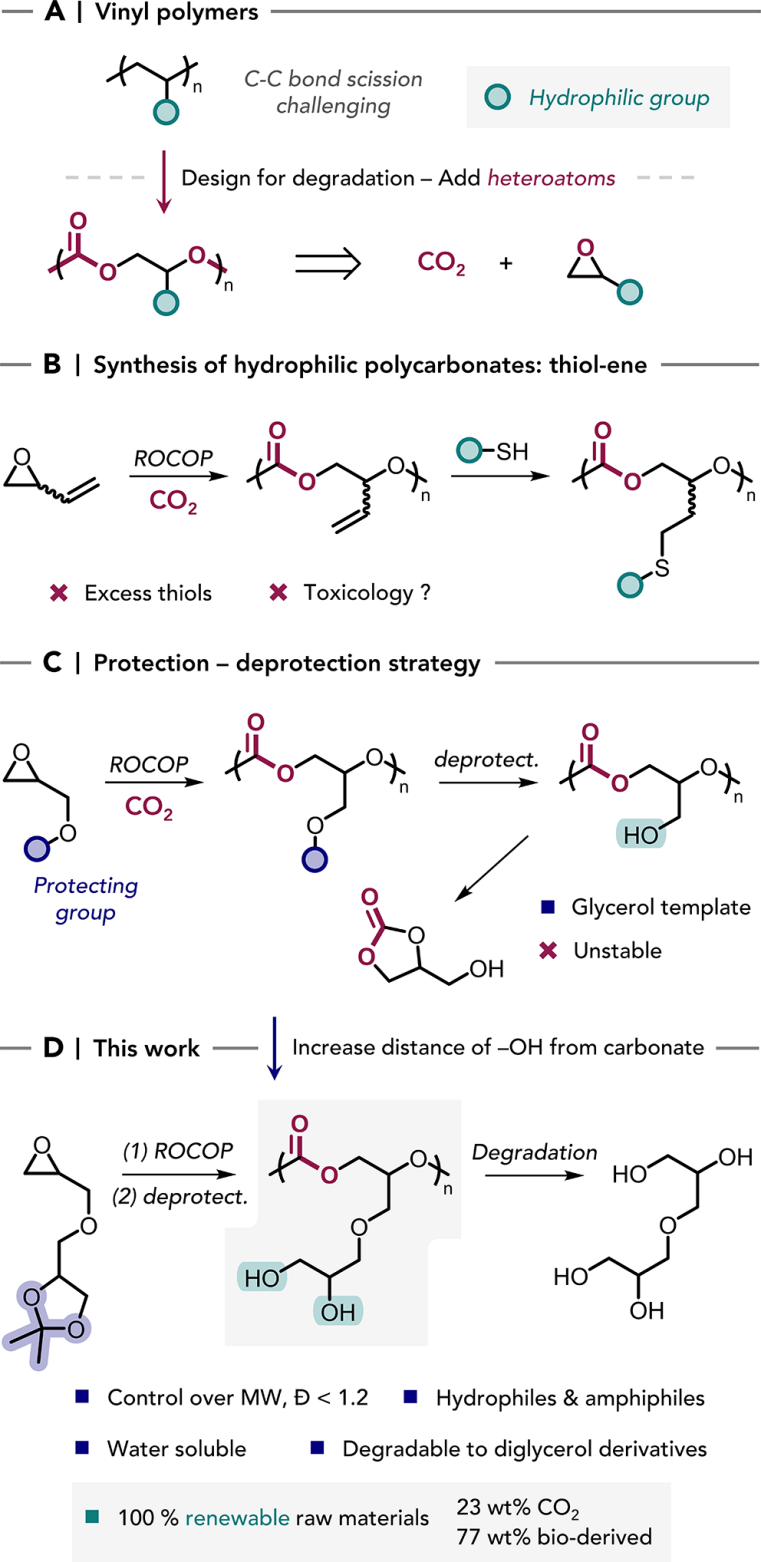
(A) Moving from Non-degradable Vinyl Polymers to Heteroatom-Rich Analogues for Degradation; The ROCOP CO_2_–epoxide Produces Carbonate-Containing Polymer Backbones; (B) Hydrophilic Polymers Made by ROCOP can be Accessed by Post-functionalization of Double Bonds by the Thiol–Ene Reaction; (C) Hydrophilic Polymers can be Obtained from Monomers Containing Protected Hydroxyl Groups; (D) This work: the Controlled Synthesis of Hydrophilic and Amphiphilic Polycarbonates Exhibiting Degradability.

One significant challenge in developing CO_2_-polycarbonates for use in aqueous formulations is installing hydrophilic functionalities to the polymer backbone, as protic groups, like alcohols or acids, act as chain transfer agents during the catalysis. One way to overcome this issue is to make polycarbonates from alkene-substituted epoxides which can be postfunctionalized with hydrophiles, e.g. by thiol–ene reaction with hydrophilic thiols (Scheme 1B).^39–45^ However, this approach requires use of excess hydrophilic thiols which gives rise to concerns over any residual thiols, which may be toxic and have very unpleasant odors, uncertain polymer stability since thioethers can be oxidized and results in the release of small-molecule byproducts of uncertain toxicology upon chain degradation. Alternatively, functionalized, but protected, epoxides may be used so that the hydrophilic functionality is inert under ROCOP conditions but can be easily deprotected after polymerization. There is good precedent for use of protecting groups including *O*-benzyl^46,47^ and *N*-benzyl groups,^48^ benzyl esters,^49,50^ and acetals.^47,51–54^ In particular, glycerol-derived polycarbonates were prepared by ROCOP of benzyl- or acetal-protected epoxides with carbon dioxide. Such an approach was used to make poly­(1,2-glycerol carbonate) featuring pendant primary alcohols (Scheme 1C). This polymer was shown to swell in water, but unfortunately the product was unstable, undergoing fast and spontaneous decomposition to form the 5-membered ring, glycerol carbonate.^46,47^ This stability issue was solved by introducing an acetal-protected diglycerol-based epoxide 1,2-isopropylidene glyceryl glycidyl ether (**IGG**). Solaro and Frey both independently attempted the ROCOP of **IGG** with CO_2_, using zinc­(II) catalysts, and also evaluated its terpolymerization with propylene oxide or glycidyl methyl ether.^51,52^ Upon deprotection, vicinal diols are unmasked, with the increased separation between the reactive hydroxyl groups and the carbonate linkages improving the polymer stability. Unfortunately, the Zn­(II) catalysts used were not so effective as they either failed to produce any **IGG**/CO_2_-polycarbonates^51^ or produced ill-defined polycarbonates with rather high dispersity (*D̵* = 2.1–2.5).^52^ None of the previously prepared polycarbonates showed any water solubility, rather they were only reported to swell in water and their degradability was not assessed. Recent advances in highly active, selective catalysts for CO_2_/epoxide ROCOP motivate the exploration of **IGG**, featuring the acetal functionality, as a building block for hydrophilic polymers. These catalysts might be able to address prior limitations in reactivity and selectivity, while also showing better tolerance to protic additives and delivering polycarbonates with controlled molecular weights, low dispersity and chain-end functionality. Such controlled hydrophilic polycarbonates would be very useful to delineate the structure–performance relationships underpinning any future use of them in aqueous formulations.^55^

## Results and Discussion

### Controlled ROCOP Catalysis Delivers Acetal-Protected Polycarbonates

The glycerol-derived epoxide monomer, **IGG**, was either synthesized on a 100 g scale from epichlorohydrin and solketal (a coproduct of biofuel production), or was commercially provided on kilogram scale. It was purified by distillation and was characterized by NMR spectroscopy (Figures S1 and S2). The ROCOP of **IGG** and CO_2_ was evaluated using a heterodinuclear [Co­(III)/K­(I)] catalyst (Figure 1A). This catalyst was previously shown to copolymerize various alkylene oxides with high activity and productivity, as well as showing good selectivity and tolerance to protic chain transfer agents. These protic species typically undergo chain transfer reactions significantly faster than polymer propagation, and therefore either end-cap (alcohols) or chain-extend (diols) the polymer chain (Scheme S1).^56^ When using diols as the chain transfer agent, it is feasible to produce di-hydroxyl telechelic polycarbonates, or polyols, with predictable molecular weights *M*_n_, whose degree of polymerization (DP) is proportional to the epoxide/initiator molar ratio.^14,57,58^ In this case, a monofunctional alcohol, methyl benzyl alcohol (MBA) was selected since it should form α-hydroxyl-ω-benzyl alcohol end-groups which are needed for subsequent investigations into diblock polycarbonate surfactants. Further, the use of methyl-benzyl alcohol (MBA) enables convenient determination of the polycarbonate *M*_n,NMR_ by end group analysis using ^1^H NMR spectroscopy. The **IGG**-CO_2_ ROCOP was conducted at 50 °C with a constant 20 bar CO_2_ pressure and using reactors equipped to enable in situ IR spectroscopy monitoring (Figure 1B). The polymerizations were conducted using [cat]/[MBA]/[**IGG**] of 1:40:1000, with an overall epoxide (**IGG**) concentration of 4.4 M (in toluene). These conditions were selected to yield polycarbonates with a mean degree of polymerization (DP) of 25.

**Figure 1:**
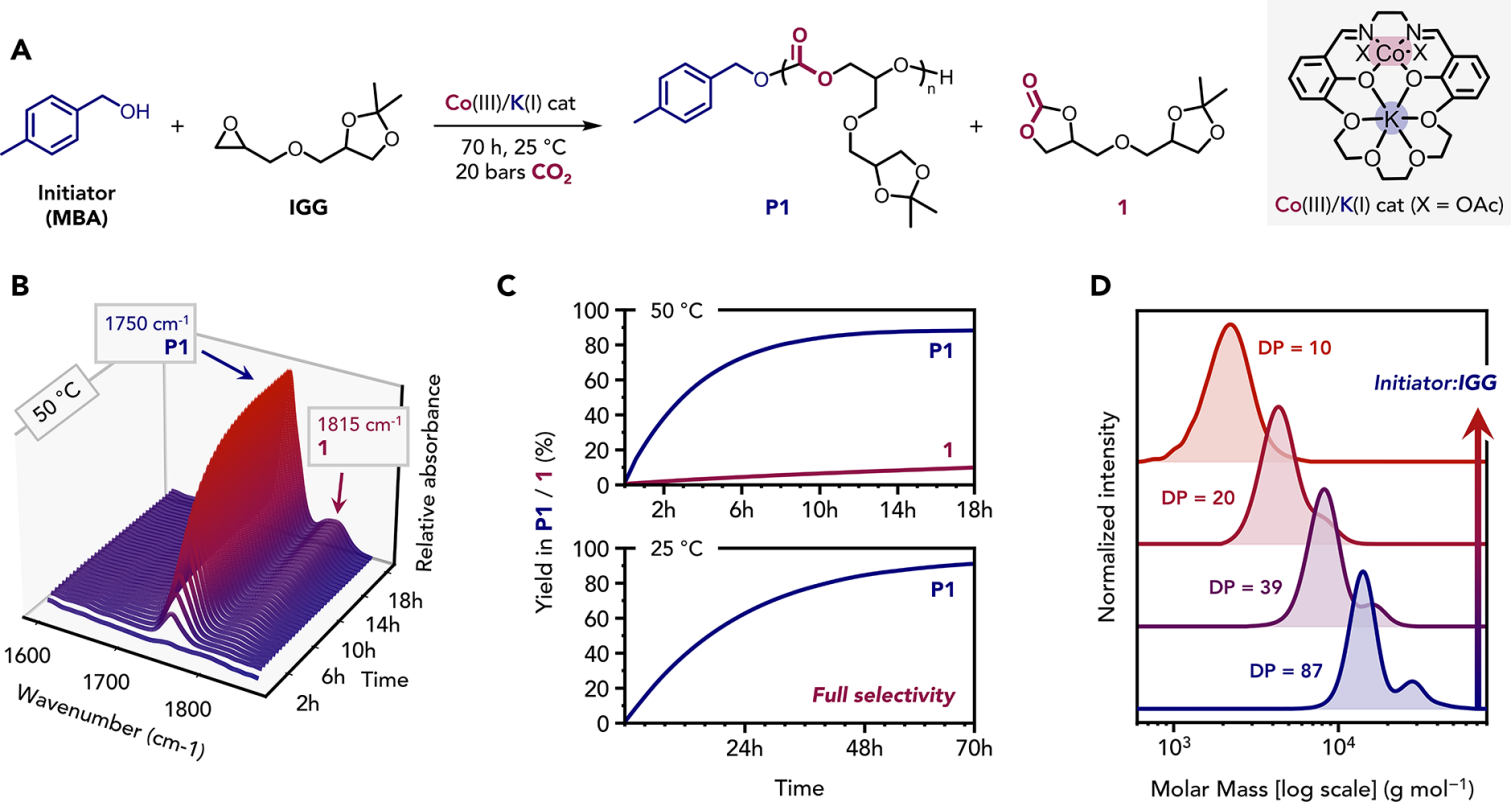
Controlled ring-opening copolymerization (ROCOP) of **IGG** and CO_2_. (A) Synthesis of **P1** from the [Co­(III)/K­(I)]-catalyzed ROCOP between **IGG** and CO_2_ using MBA as initiator. **1** is obtained as a side product. (B) In situ IR spectroscopy monitoring of the CO_2_-**IGG** ROCOP at 50 °C (Table 1, entry 1). (C) Formation of **P1** and **1** vs time curves obtained for the polymerizations conducted at either 50 °C (top) or 25 °C (bottom) to produce polycarbonate (**P1**) or the byproduct **1**. (D) Stacked SEC traces (in THF) of pure derivatives of **P1** with various mean degrees of polymerization (DP).

Monomer conversion and selectivity were continuously monitored, using in situ IR spectroscopy, by following the growth of the polymer **P1** carbonate band at 1750 cm^–1^, and the cyclic carbonate byproduct **1** at 1815 cm^–1^, respectively (Figure 1B). ^1^H NMR spectroscopy analysis of the crude reaction mixture was used to quantify the final monomer conversion and yields for **P1** and **1** (Figure S3). The polymer formation occurred rapidly over the first 8 h before reaching a plateau, whereas the formation of **1** proceeded much more slowly over time owing to slower backbiting cyclization occurring from the chain ends (Figure 1C, top). As the aim is to produce polymers with predictable chain lengths, this cyclic carbonate side reaction must be suppressed. By performing the **IGG**/CO_2_ ROCOP at 25 °C, the catalyst selectivity for **P1** increased from 90 to 98% (Figure S4). The lower temperatures needed for the improved selectivity result in longer reaction times, with 91% **IGG** conversion requiring ∼70 h under these conditions (Figure 1C, bottom).

The purified polycarbonate was characterized by ^1^H and ^13^C NMR spectroscopy (Figures S5 and S6). The selective formation of carbonate linkages was evidenced by the presence of the -CH- signal (δ_1H_ = 5.02 ppm) directly adjacent to the carbonate function (δ_13C_ = 154 ppm). The acetal protecting group remained intact after polymerization and purification of the polymer, as highlighted by the two strong -CH_3_ resonances (δ_1H_ = 1.34 and 1.40 ppm). The characteristic -CH_3_ signal (δ_1H_ = 2.34 ppm) of the chain-end group (MBA) was used to determine the polycarbonate mean DP of 20, which matches the value expected from the catalyst loading and monomer conversion.

Systematic variation of the catalyst/initiator (MBA) loading provided control over the resulting polycarbonate *M*_n_, as expected since it controls the number of initiated chains, providing **P1** samples with *M*_n_ values ranging from 2000 to 20,000 g mol^–1^ and consistently high **IGG** conversions (>79%) with low dispersities (D̵ ≤ 1.13) (Table 1). Reducing the initiator loading vs catalyst resulted in a high molecular weight shoulder in the SEC trace owing to some chain initiation from diols (Figure 1D).^57^ *M*_n_ values determined from ^1^H NMR spectroscopy by integration of the end (7.16 ppm) vs main chain (5.02 ppm) groups were consistent with values determined by SEC in THF, suggesting only minor differences in hydrodynamic volume when using polystyrene calibration standards in this molar mass regime (Figure S7).

**Table 1:** Synthesis of **P1** by the ROCOP of CO_2_ and **IGG** with Varying Amounts of Methyl Benzyl Alcohol (MBA) as Initiator^a^

entry	MBA initiator (equiv)	*M*_n,target_ ^c^ (g mol^–1^)	IGG Conv.^d^ (%)		P1 DP^e^	*M*_n,NMR_ ^e^ (g mol^–1^)	*M*_n,SEC_ ^f^ (g mol^–1^)	*D̵* ^f^
				P1 Select.^d^ (%)				
1^b^	40	5900	98	90	19	4500	4400	1.09
2	10	23,300	81	95	87	20,300	14,900	1.13
3	20	11,700	79	94	39	9200	8300	1.11
4	40	5900	91	98	20	4800	4300	1.08
5	80	3000	85	96	10	2400	2100	1.08

a Reaction conditions: [cat]:[MBA]:[**IGG**] = 1:*x*:1000 where x is varied from 10 to 80, dry toluene, 25 °C, 70 h, 20 bar CO_2_, [**IGG**] = 4.4 M.

b Reaction performed at 50 °C for 18 h.

c Targeted *M*_n_ calculated from theoretical initiator loading (i.e., targeted DP) at full monomer conversion and selectivity: (DP_targeted_ × *M*_n,repeating unit_) + *M*_n,MBA_.

d Conversion in **IGG** and selectivity in **P1** vs **1** measured by ^1^H NMR spectroscopy (CDCl_3_) using the crude product (see Supporting Information for further information).

e Molar mass determined by NMR: (DP × *M*_n,repeating unit_) + *M*_n,MBA_ on the pure polymer.

f Molar mass determined by SEC analysis using the pure polymer, with THF as eluent and polystyrene calibration standards. Dispersity, *D̵* = *M*_w_/*M*_n_.

MALDI-TOF analysis of the polycarbonate with a mean DP of 20 shows two distributions assigned to chains associated with either potassium cations or protons, respectively. No other polymer series were detected, suggesting the selective formation of the target polycarbonate, without any ether linkages. The experimentally determined repeat unit mass (232.20 g mol^–1^) closely matched the theoretical value (232.09 g mol^–1^) (Figure 2A). Plotting *m*/*z* vs number of repeat units (*N* ^th^) showed a gradient with the expected repeat unit together with the target MBA and hydroxyl end-groups (Figure 2B).

**Figure 2:**
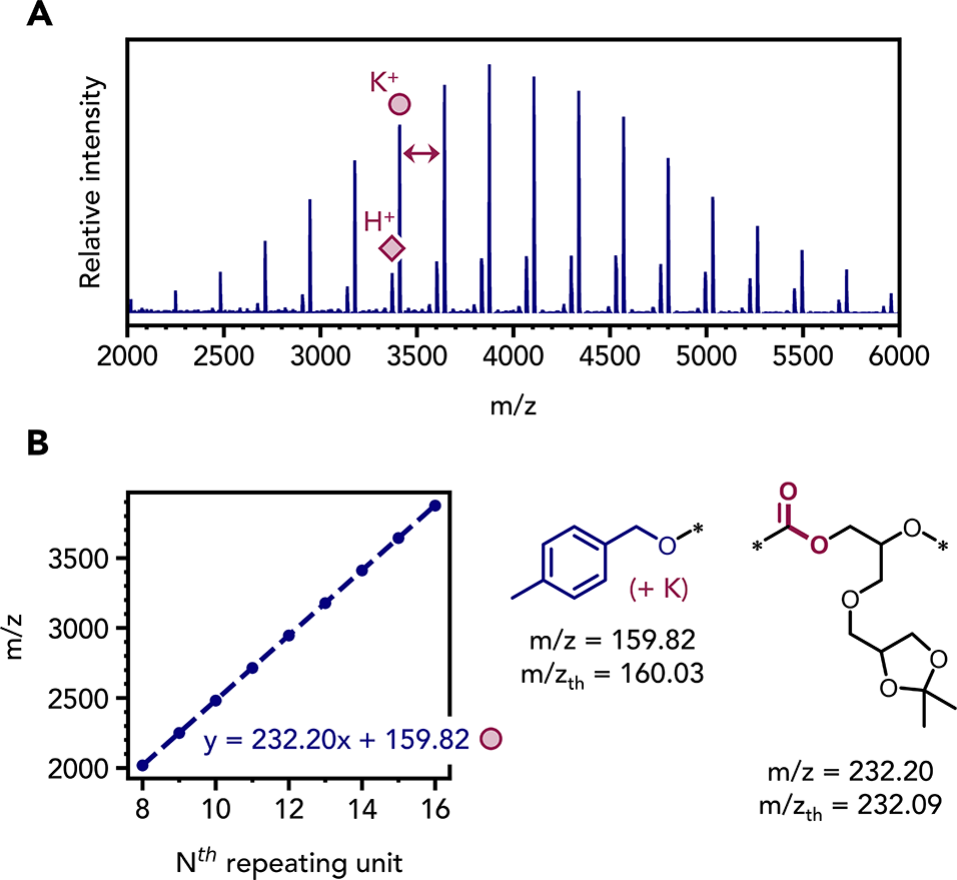
(A) The MALDI-TOF spectrum for **P1** (DP 20). (B) Plot of *m*/*z* vs N^th^ repeat unit, with experimental and theoretical *m*/*z* values for the poly­(**IGG**-*alt*-CO_2_) and MBA end-group.

Thermal gravimetric analysis (TGA) of the polycarbonates show an onset degradation temperature (*T*_deg,5%_) ranging from 180 to 230 °C (Figure S8). DSC analyses revealed that all the polycarbonates are amorphous and, as expected for such low molar mass samples, the glass transition temperatures (*T*_g_) increase with the polymer DP, from −36 °C (DP = 10) to 3 °C (DP = 87) (Figure S9).

Overall, the [Co­(III)/K­(I)] catalyst shows excellent polymerization control and conversion; it is fully compatible with the sensitive acetal **IGG** functionality. The resulting polycarbonates have predictable *M*_n_ values and narrow molecular weight distributions. Polymer **P1** (DP = 20) was selected as the lead sample for further characterization and degradation studies.

### Hydrophilic Polycarbonates

Next, deprotection of the **P1** acetal substituents was targeted to obtain hydrophilic, diol-containing polycarbonates **P1d**. Previously, other researchers reported the acetal deprotection of related **IGG**-derived polyethers using a solid acid catalyst with mixtures of aprotic organic solvents (e.g., THF) and protic solvents (methanol or water) at slightly elevated temperature (40–50 °C).^59–61^ This method was attempted for **P1**, but we encountered reproducibility issues and contamination of the polymer with catalyst residues. To overcome this issue, an alternative deprotection strategy involving aqueous hydrochloric acid (HCl) as an inexpensive mineral acid enabled efficient removal of the acetal groups. After optimization, it was found that a 1:1 acetonitrile/water solution of **P1** (100 mg mL^–1^), with HCl (0.6 equiv. vs acetal linkages), led to quantitative deprotection of **P1** within 1 h at 25 °C (Figure 3A). The polymer deprotection reaction was monitored by ^1^H NMR spectroscopy, which showed progressive loss of the acetal –CH_3_ signals at 1.24 and 1.29 ppm over time until they were no longer detectable, along with a constant polycarbonate backbone -CH- signal at 4.97 ppm (Figures 3B and S10).

**Figure 3:**
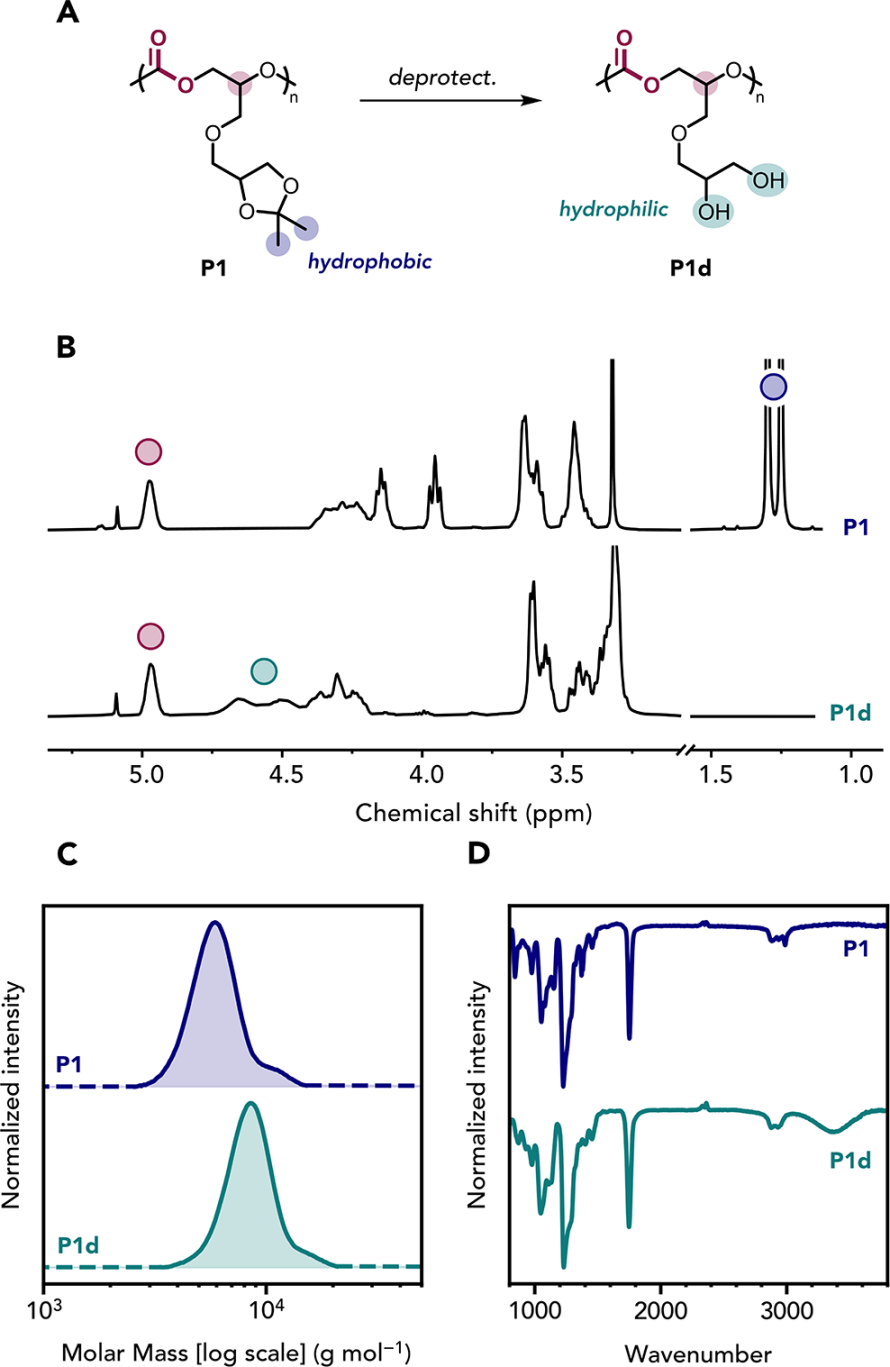
Deprotection of the acetal-containing polymer **P1**. (A) Deprotection of **P1** into **P1d**. Reaction conditions: **P1** (100 mg mL^–1^), HCl (0.6 equiv. vs polymer linkage), v/v acetonitrile/water = 1:1, 25 °C, 1 h. (B) ^1^H NMR spectra (400 MHz, DMSO-*d*_6_), (C) SEC data (DMF/LiBr as eluent), and (D) ATR-IR spectra recorded for pure **P1** (top) and **P1d** (bottom).

Following purification, **P1d** was recovered in high yield (93%) and characterized by NMR spectroscopy (Figures S11 and S12), SEC (Figure 3C) and MALDI-TOF (Figure S13). Notably, these hydrophilic polycarbonates are entirely renewable, comprising 23 wt % CO_2_ and 77 wt % biobased, glycerol-derived molecules. The formation of the diol chain substituents was confirmed by the complete disappearance of the acetal methyl protons, and the appearance of broad -OH resonances (δ_1H_ = 4.8–4.4 ppm) in the purified samples of **P1d** (Figure 3B). The end-group (MBA) vs main chain group integrals were the same as those for **P1**, confirming that the polycarbonate backbone remained intact after deprotection (Figure S11). As **P1d** is insoluble in THF, the SEC traces of both **P1** and **P1d** were compared using DMF as the eluent: identical peak shapes and similar retention times were observed, with only a slight shift toward higher apparent molar mass for **P1d** (Figure 3C). This shift is attributed to a subtle change in the hydrodynamic volume of **P1d** (vs **P1**) and may arise from polymer–column interactions. MALDI-TOF analysis confirmed the expected repeat unit molar mass and chain end-groups (MBA and hydroxyl), without any additional distributions (Figure S13). Infrared (IR) spectroscopy of **P1** and **P1d** both showed the carbonate carbonyl band, along with the appearance of a broad O–H stretch at 3400 cm^–1^ for **P1d** (Figure 3D). The carbonate band was also slightly broadened in **P1d**, suggesting hydrogen bonding between the newly formed hydroxyl groups and the carbonate linkages (Figure S14). Although **P1d** was insoluble in THF, it proved to be highly soluble (>100 mg mL^–1^) in polar aprotic solvents (e.g., DMF or DMSO) and, importantly it showed high solubility in water. **P1d** exhibits a slightly higher decomposition temperature than **P1** (*T*_deg,5%_ = 227 °C) and a higher *T*_g_ value (from −17 for **P1** to 4 °C for **P1d**) (Figure S15), which is consistent with hydrogen bonding interactions reducing chain mobility.^62^

**P1** and **P1d** show remarkably different physical properties: **P1** is a viscous liquid whereas **P1d** forms a self-supporting film. Rheological measurements were conducted to assess both polymer properties within the viscoelastic region, at lower and upper temperatures as evaluated by amplitude sweeps (Figures S16 and S17). Time–temperature superposition (TTS) experiments were used to obtain master curves over a wider frequency range (Figures S18 and S19). In the terminal (low frequency) regime, both materials exhibited Rouse-like behavior that is typical of unentangled polymer melts, with a power law exponent for *G*′ approaching 2.^63^ For **P1**, this trend was observed up to the crossover between *G*′ and *G*″ at higher frequency (Figure 4A). In contrast, **P1d** showed some deviation in the medium-frequency regime: the power law exponent for *G*′ was reduced to 0.9, nearly matching that of *G*″, with both moduli evolving in parallel with frequency (Figure 4B).^64^ This behavior was previously reported for weakly hydrogen-bonded polymers and is attributed to a transient cluster regime controlled by the dynamic formation and dissociation of reversible hydrogen bonds, generating microstructures within the polymer melt.^63,65^ This behavior is responsible for the additional relaxation regime observed for **P1d**, before complete chain relaxation in the terminal regime. Notably, the crossover point at high frequency, where the storage modulus becomes greater than the loss modulus (i.e., when the chains do not have sufficient time to relax and exhibit an elastic behavior^66^) shifted to lower frequencies for **P1d**, with the terminal relaxation time^67^ increasing from 0.011 s for **P1** to 0.053 s for **P1d**. The zero-shear viscosity (η_0_) of **P1d** revealed a striking 288-fold increase at 20 °C, compared to the acetal-protected polycarbonate **P1**, which is consistent with enhanced intermolecular interactions via hydrogen bonding (Figure 4C).

**Figure 4:**
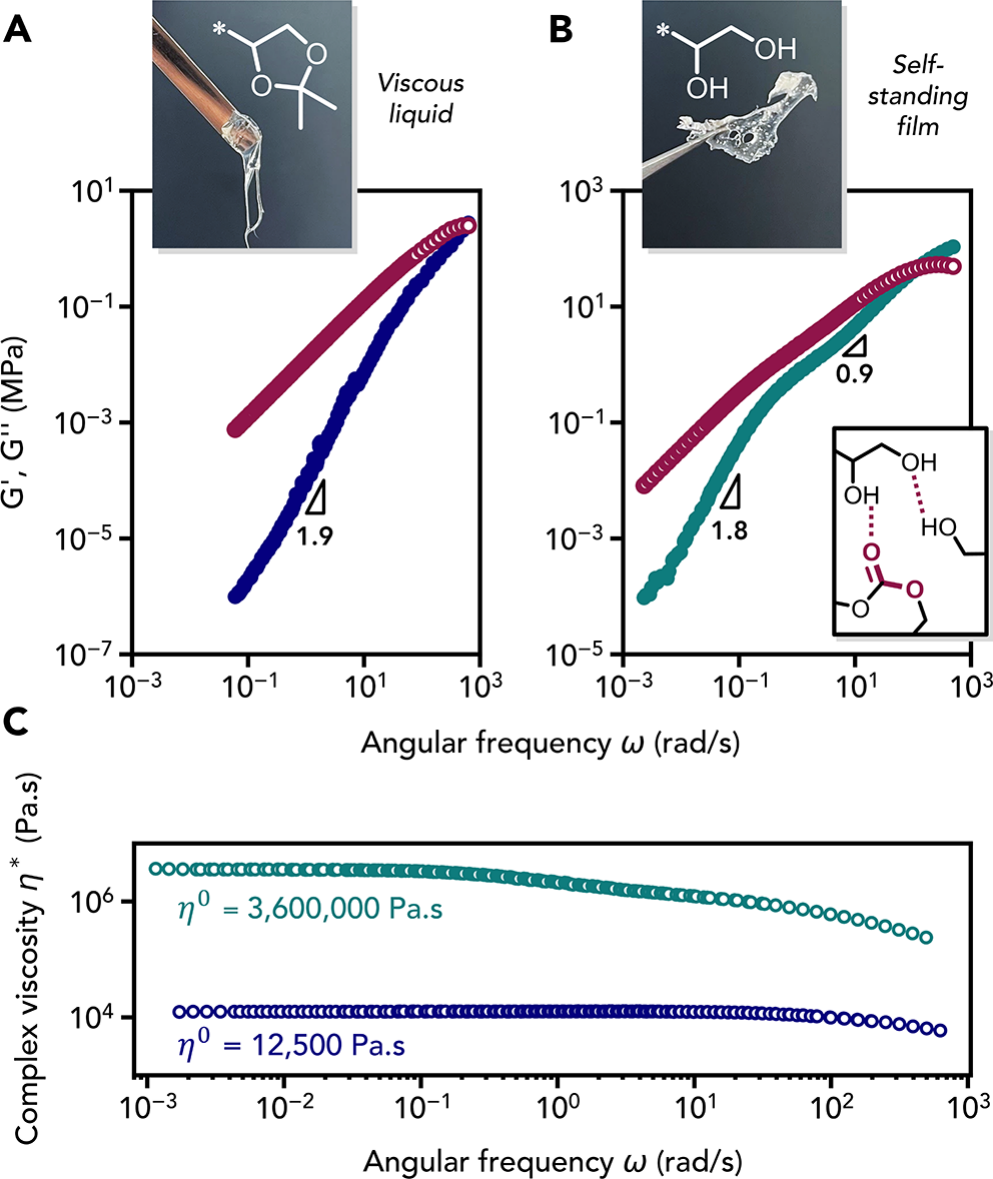
Rheological characterization of polycarbonates **P1** (before deprotection) and **P1d** (after deprotection). Master curves of (A) **P1** and (B) **P1d** constructed by time–temperature superposition (TTS) referenced to 20 °C: storage (*G*′) and loss (*G*″) moduli vs angular frequency. (C) The zero-shear viscosity η_0_ at 20 °C was determined from the complex viscosity η*.

### Hydrophilic and Amphiphilic Polycarbonates

Having established a consistent and effective preparation route for hydrophobic acetal polycarbonates that can be transformed into hydrophilic diol-containing analogues, we next targeted a series of polycarbonates with tailored solution properties. We targeted materials with the following property profiles: (i) hydrophilic polycarbonates (**P1d**) without the aromatic end-groups that may be undesirable in certain applications, and (ii) amphiphilic polycarbonates, comprising both hydrophilic and hydrophobic components, so as to investigate the aqueous solution self-assembly. Controlling the nature of the initiator used in the catalysis should offer a straightforward route to introduce functional end-groups into the polymers, including hydrophobic chains/substituents.^14,68–70^

To obtain a purely hydrophilic polycarbonate, solketal was selected as the initiator since it is a direct mimic of the **IGG** polymer repeat unit and, upon deprotection, should generate the same hydrophilic diol functionality (Figure 5A). ROCOP was conducted between **IGG**/CO_2_ using the same [cat]/[solketal]/[**IGG**] molar ratios employed to prepare **P1** (1:40:1000, DP = 25) and yielded polymer **P2**. The ^1^H NMR spectrum of the crude reaction product confirmed full **IGG** conversion with quantitative (>99%) selectivity for **P2**. End-group analysis by ^1^H NMR spectroscopy, recorded in CDCl_3_, was hindered by peak overlap, since the solketal end-group signal is almost identical to the acetal repeat units. Fortunately, the ^1^H NMR spectrum recorded in DMSO-*d*_6_ enabled reliable integration of the terminal hydroxyl signals, affording a mean DP of 22 (Figures S20 and S21). SEC analysis (in THF) of the polycarbonate indicated an *M*_n_ value consistent with that calculated by ^1^H NMR spectroscopy (*M*_n,NMR_ = 5200 g mol^–1^; *M*_n,SEC_ = 5700 g mol^–1^) (Figure S22). Quantitative deprotection of the acetal groups to afford the fully hydrophilic analogue **P2d** was achieved within 1 h, following the same protocol described above (Figures S23–S25). The thermal analysis data obtained for **P2**/**P2d** were similar to those for **P1**/**P1d** (Figures S26 and S27).

**Figure 5:**
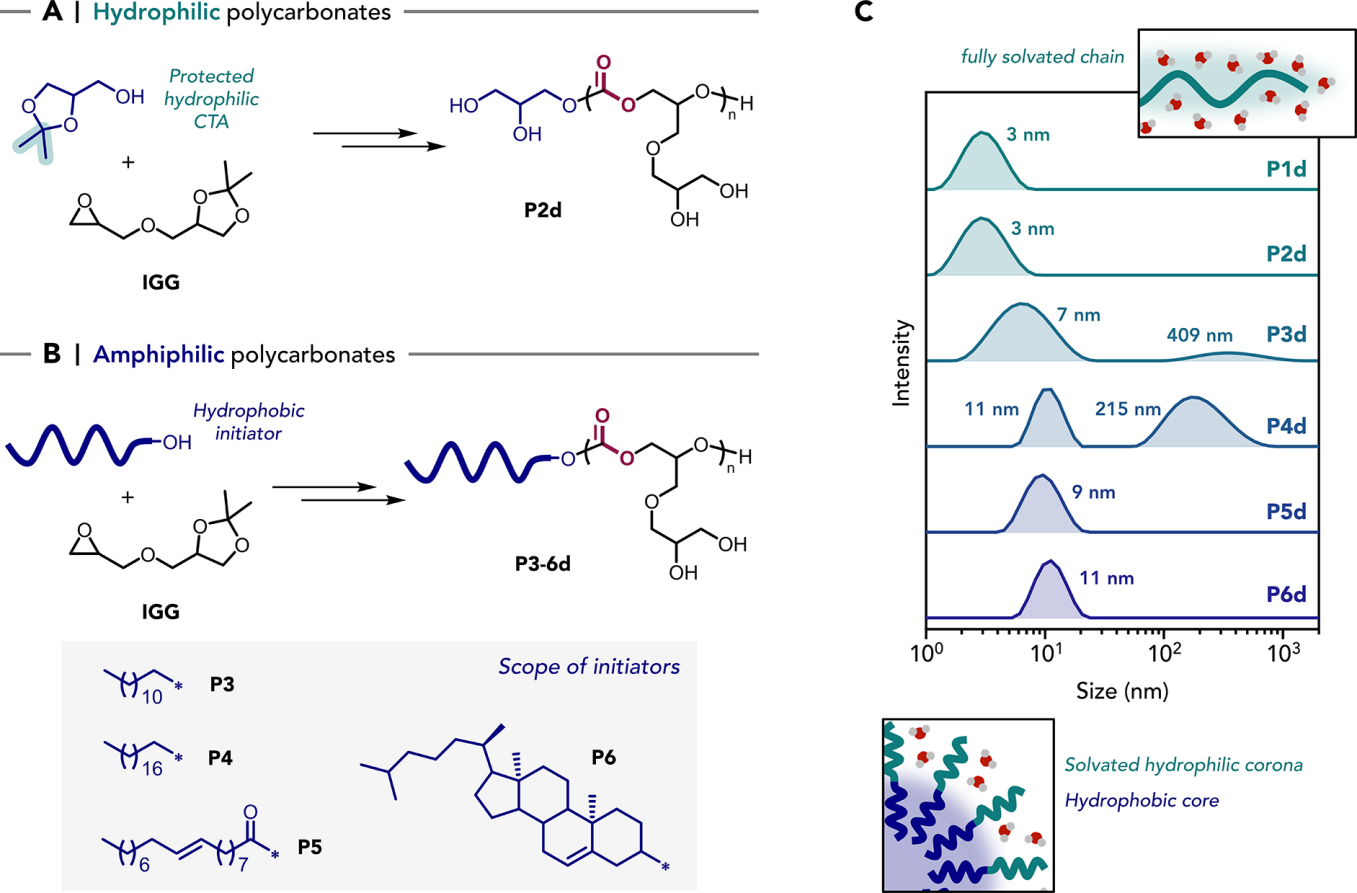
Synthesis of polymers **P2–6** and their deprotected analogues **P2d–6d** through variation of the initiator type. (A) Synthesis of **P2** using solketal as initiator, with deprotection yielding a fully hydrophilic polymer **P2d**. (B) Synthesis of **P3–P6** using hydrophobic initiators, yielding the corresponding amphiphilic polymers **P3d**-**P6d** after deprotection. (C) DLS particle size distributions recorded for 10 mg mL^–1^ aqueous solutions of the polymers.

A series of amphiphilic polycarbonates were targeted using four different hydrophobic natural products as the initiators: lauryl alcohol (**P3**), stearyl alcohol (**P4**), oleic acid (**P5**), and cholesterol (**P6**) (Figure 5B). To examine whether using such hydrophobic initiators affected the polymerization, in situ IR spectroscopy was used to monitor **P3** formation. The ROCOP to make **P3** showed exactly the same polymerization kinetic profile as that measured during the formation of **P1** (initiated from MBA), which shows that the catalyst is very tolerant to a range of different initiators (Figure S28). The ROCOP reached 93% **IGG** conversion, with polycarbonate selectivity of 99%, and the resulting polycarbonate has a monomodal molar mass distribution (Figure S29). For **P3**, the ^1^H NMR spectrum clearly shows the characteristic resonances of the –CH_2_– in the α position of the carbonate (δ_1H_ = 4.11 ppm), the more shielded –CH_2_– in the hydrophobic end-group (δ_1H_ = 1.65 and 1.22 ppm), and the terminal –CH_3_ (δ_1H_ = 0.86 ppm) from which a mean DP of 24 was determined (Figures S30–S31). MALDI-TOF analysis confirmed incorporation of the long-chain initiator end-group (Figure S32).

The other initiators were also successfully applied in the ROCOP and, in all cases, reactions achieved high **IGG** conversions and excellent polycarbonate selectivity values (Table 2), forming materials with predictable molar masses and low dispersity (Figures S33–S50). In the case of **P5**, which was initiated from a carboxylic acid (oleic acid), MALDI-TOF revealed a chain-end corresponding to an oleate-**IGG** unit, consistent with initiation via reaction of the carboxylic acid with the epoxide, whereas alcohols initiate chains via direct CO_2_ insertion (Figure S42). The ^1^H and ^13^C­{^1^H} NMR spectra of pure **P3**–**P6** confirmed the characteristic resonances from both the polycarbonates and the hydrophobic initiator end-groups. All polymers exhibited similar thermal stability and *T*_g_ values to those obtained for **P1**, with *T*_deg,5%_ values in the range 200–210 °C and the desirable low *T*_g_ values from −25 to −2 °C (Figures S51and S52).

**Table 2:** Synthesis of Polycarbonates (**PC**) **P2-6** From the ROCOP of CO_2_ and **IGG** Using Various Initiators^a^

entry	polymer (PC)^b^	IGG Conv.^c^ (%)	PC	PC	*M*_n,NMR_ ^d^ (g mol^–1^)	*M*_n,SEC_ ^e^ (g mol^–1^)	*D̵* ^e^
			select.^c^ (%)	DP^d^			
1	**P2**	99	99	22	5200	5700	1.14
2	**P3**	93	99	24	5700	6700	1.12
3	**P4**	95	99	21	5100	5700	1.17
4	**P5**	75	95	18	4500	4900	1.09
5	**P6**	64	94	16	4100	3900	1.09

a Reaction conditions: [cat]:[initiator]:[**IGG**] = 1:40:1000, dry CH_2_Cl_2_, 25 °C, 70 h, 20 bar CO_2_, [**IGG**] = 4.4 M.

b Initiator = solketal (**P2**), lauryl alcohol (**P3**), stearyl alcohol (**P4**), oleic acid (**P5**), cholesterol (**P6**).

c Conversion of **IGG** and selectivity for polymer vs **1** measured by ^1^H NMR spectroscopy in CDCl_3_ on the crude reaction product (see Supporting Information for further information).

d Molar mass determined by NMR: (DP × *M*_n,repeating unit_) + *M*_n,initiator_ on the pure polymer.

e Molar mass determined by SEC analysis (THF as eluent, polystyrene calibration standards) of the pure polymer. Dispersity *D̵* = *M*_w_/*M*_n_.

The hydrophobic segment in the polymer chains does not hinder quantitative deprotection of the acetal groups, which was achieved in all cases within 1 h and which was confirmed by the complete disappearance of the characteristic resonances at 1.24 and 1.29 ppm in the ^1^H NMR spectra. SEC analysis (in DMF) of the hydroxyl-functionalized polymers confirmed that the polycarbonate backbone remained stable under acidic conditions, in all cases. Polymers **P3d**–**P6d** exhibited *T*_deg,5%_ values of 220–240 °C (Figure S53) and slightly higher *T*_g_ values (−12 to −4 °C). Notably, **P4d** displayed a melting transition at 14 °C and a *T*_g_ value at −5 °C, with the former thermal transition attributed to partial crystallization of the stearyl chains in the polymer melt (Figure S54).^71^ In contrast, **P3d**, which features a shorter C_12_ end-group vs C_18_ for **P4d**, remained amorphous.

All the polymers show excellent water solubility over the concentration range 1–50 mg mL^–1^. Importantly, they could be directly dispersed in water without solvent exchange or thermal/pH triggers, and several of them spontaneously self-assembled under these simple conditions, a feature which could be useful for making surfactants at larger-scale.^72–74^ Dynamic light scattering (DLS) measurements were performed on 10 mg mL^–1^ aqueous solutions of **P1d**–**P6d** to evaluate any self-assembly of the polymer (Figure 5C). **P1d** and **P2d** both show hydrodynamic diameters below 3 nm, suggesting that they form molecularly dissolved chains in water. Polymers **P5d** and **P6d** each show a unimodal size distribution, with hydrodynamic diameters of 9 and 11 nm, respectively, which indicates the formation of small micelles or nanoparticles. **P3d** shows a bimodal distribution, with a major population at 7 nm and a minor population at 409 nm. **P4d** presents a primary peak at 11 nm and a relatively intense secondary population at 215 nm. Overall, these DLS results suggest that both oleic acid- and cholesterol-functionalized polymers form relatively small, well-defined micelles, whereas long linear alkyl chains promote the formation of larger aggregates. It must be noted that DLS is particularly sensitive to the presence of larger assemblies, even when they are present as a minor population. To further investigate the nature of the minor larger nanostructure population observed for **P4d**, cryogenic transmission electron microscopy (cryo-TEM) images were acquired and showed rod-like assemblies (Figure S55). These larger nanostructures are responsible for the secondary size distribution observed by DLS.

**P4d** was selected for further characterization as a potential degradable nonionic polymeric surfactant. Surface tensiometry measurements indicated a critical micelle concentration (CMC) of approximately 30 mg L^–1^, with a limiting surface tension of 55.8 mN m^–1^ (Figure 6 and Figure S56). In contrast, the more hydrophilic **P1d** exhibited no CMC up to 50 mg mL^–1^, indicating no surface activity in this case.

**Figure 6:**
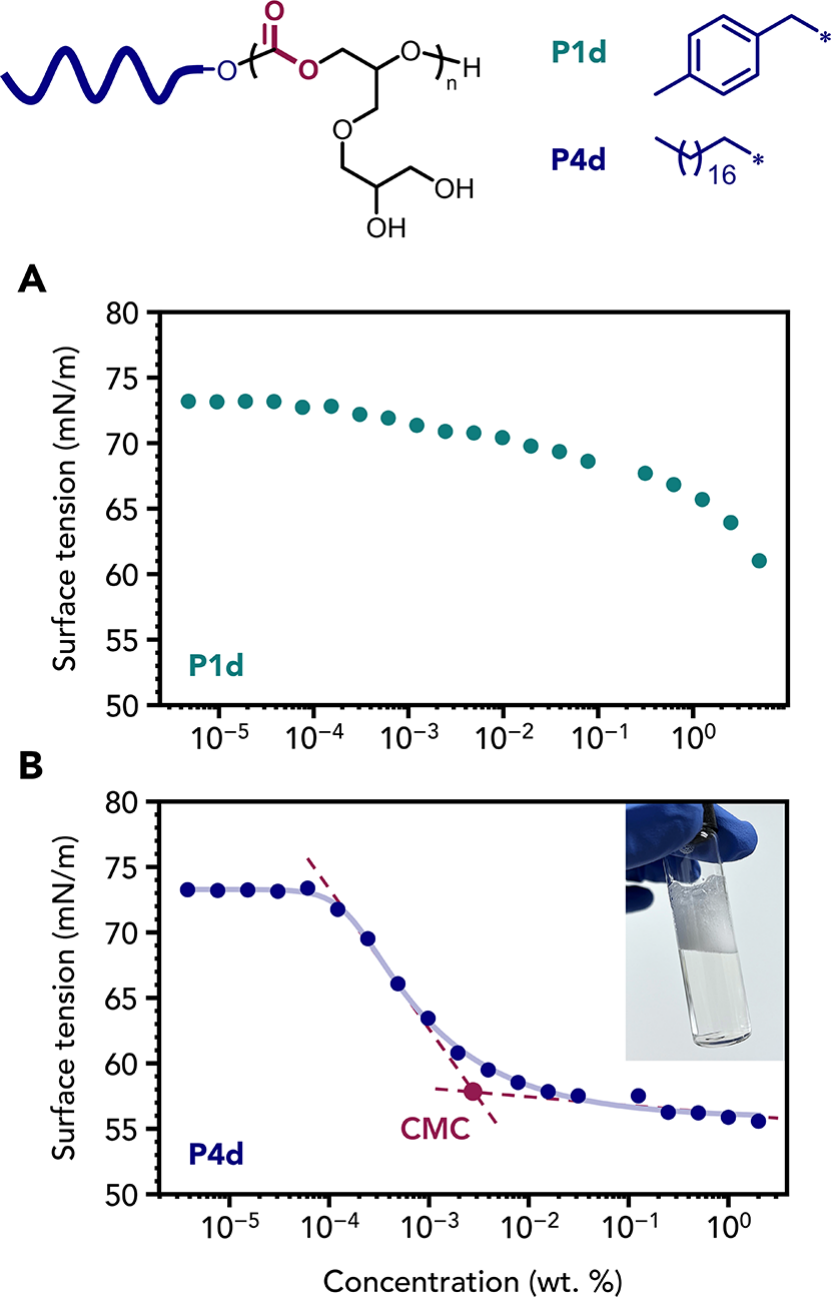
Aqueous surface tension vs concentration plots obtained for polymers (A) **P1d** and (B) **P4d** dissolved in water.

### Polycarbonate Degradability

Hydrolytic degradation in water is a critical feature for many aqueous formulations, as most products will ultimately enter wastewater streams, it is also clearly a feature which must be controlled during the formulation’s use phase.^75,76^ Understanding both the extent of depolymerization over time and the identity of the degradation products is essential. Polycarbonates bearing reactive pendant primary hydroxyl groups adjacent to the carbonate linkage are known to undergo spontaneous depolymerization, via intramolecular cyclization to form the stable cyclic carbonates.^46,47,77^ For these polycarbonates, the diglycerol-derived backbone increases the distance between hydroxyls and carbonate groups, which should increase their aqueous stability and prevent spontaneous decomposition (Scheme 1). However, the precise conditions and polymer chemistries that enable long-term aqueous stability as opposed to hydrolytic degradation are not well-established.

Investigations of the hydrolysis of aliphatic polycarbonates, prepared by ROCOP of CO_2_ and epoxides, are particularly scarce. Poly­(limonene carbonate) functionalized with hydrophilic groups, including a short PEG chain or an alcohol, was shown to be stable for 4 weeks in basic aqueous solution (pH 9) at 37 °C.^39^ In contrast, polymers bearing pendant ester groups, such as poly­(ethyl acrylate carbonate) or poly­(glycidyl ether ester carbonate), underwent hydrolysis to form CO_2_ and alcohols when stored in a THF/buffered water mixture at pH 5, 7 or 9.^78^ Given the significant differences in hydrolysis rates, it is clearly important to investigate the aqueous properties and degradation kinetics of the new hydrophilic polycarbonates, derived from diglycerol and carbon dioxide.

First, a 25 mg mL^–1^ aqueous solution of the hydrophilic polymer **P2d** was established to be highly stable when stored at 25 °C for more than 1 month at either neutral or acidic pH (pH 1) (Figure S57). The complete degradation of **P2d** occurred within 10 min in mildly alkaline solution, at 25 °C (sodium carbonate buffer, pH 10, Figure S58). The ^1^H NMR spectrum of the crude reaction mixture, in D_2_O, confirmed the complete disappearance of the polymer backbone -CH- resonance (δ_1H_ = 5.14 ppm), and the formation of a well-resolved multiplet attributed to the -CH- resonance (δ_1H_ = 5.07 ppm) for diglycerol carbonate **2** (Figure S58). This assignment was confirmed by independent synthesis of **2**, via the cycloaddition of CO_2_ and **IGG** to yield the cyclic carbonate precursor **1**, followed by acetal group removal to yield **2**. Products **1** and **2** were fully characterized by ^1^H NMR spectroscopy and high-resolution mass spectrometry (HRMS) (Figures S59–S62).

Next, the hydrolytic degradation of a 25 mg mL^–1^ aqueous solution of the polycarbonate **P2d** at pH 8 (sodium phosphate buffer) was monitored by ^1^H NMR spectroscopy (in D_2_O) using DMSO-*H*_6_ as the internal standard. Monitoring the characteristic resonances for **P2d** and the cyclic carbonate **2** revealed that the degradation at pH 8 was much slower than that at pH 10 (Figures S63 and S64A). Nevertheless, at pH 8, 95% of **P2d** degraded within 8 h at 25 °C and complete degradation was achieved within 24 h. The polymer backbone degradation appeared to be pseudo zero-order in polymer concentration up to ≈50% conversion, followed by a regime better described by first-order kinetics (Figure S64B, S65). This mixed kinetic profile is characteristic of self-immolative polymers, whereby degradation begins at the chain end-groups. In such reactions, at the beginning of the degradation the end-group concentration is constant, which results in zero-order kinetics.^79,80^ As depolymerization progresses, the progressive formation of short oligomers, increases the dispersity of the chains, and a decline in the concentration of active end-groups leads to more complex kinetics. Formation of the cyclic carbonate **2** by a backbiting chain-unzipping mechanism initiated from the hydroxyl end-groups is consistent with this interpretation (Scheme 2).

**Scheme 2:**
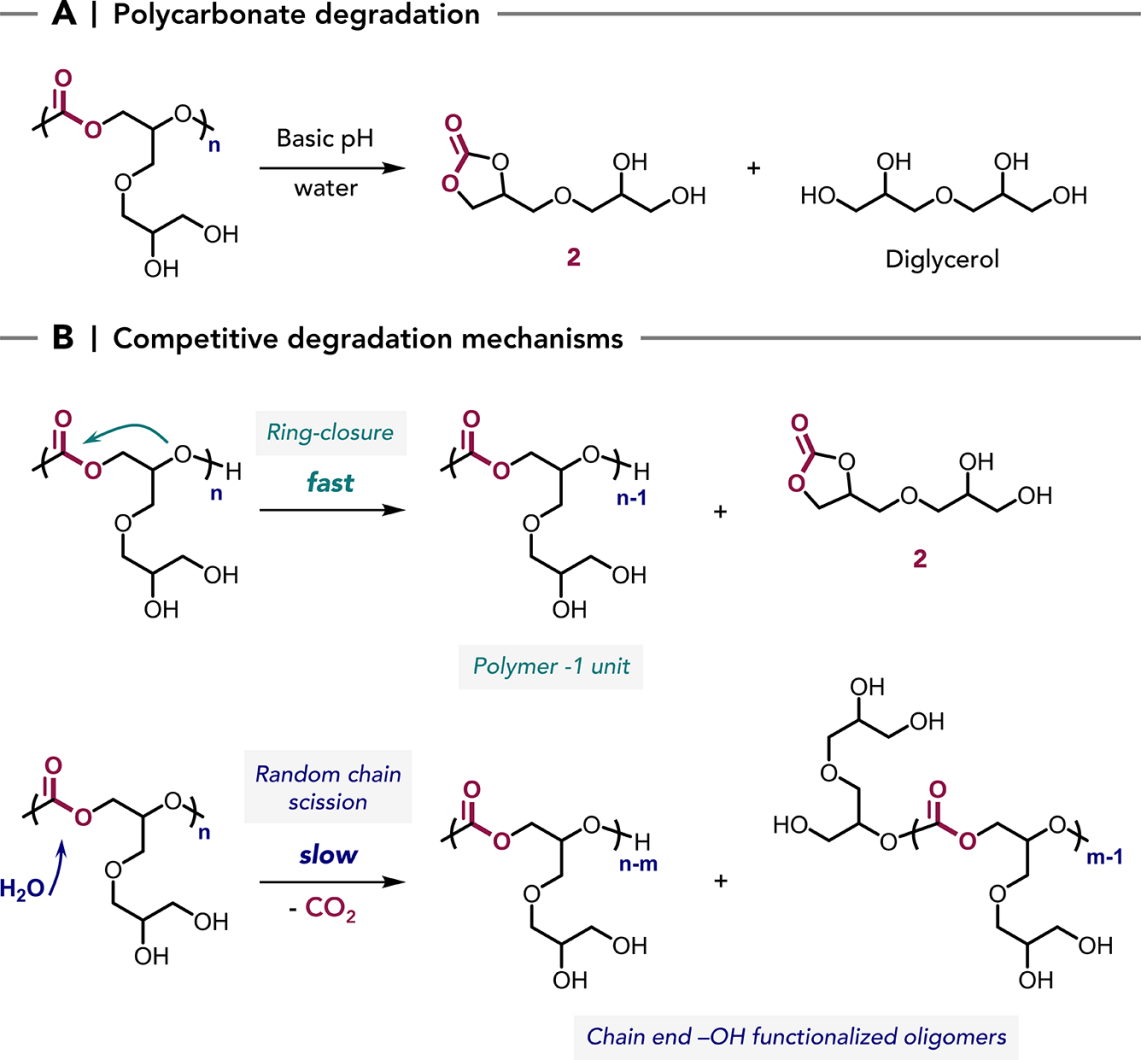
Hydrolytic Degradation of the Deprotected Hydroxyl-Functional Polycarbonates in Aqueous Solution at 25 °C

a (A) Degradation of deprotected **IGG**-derived polycarbonate to afford **2** and diglycerol. Reaction conditions: polymer (25 mg mL^–1^) in mildly alkaline aqueous solution at either pH 8 (phosphate buffer) or pH 10 (carbonate buffer), 25 °C. (B) Proposed competitive degradation mechanisms in basic aqueous solution: (i) rapid chain-end hydroxyl-initiated backbiting (ring-closure) vs (ii) slow random chain scission by backbone hydrolysis.

The molar mass of the degrading polycarbonate was periodically analyzed by SEC. A progressive reduction in *M*_n_ was observed, while the corresponding dispersity remained low, e.g. *M*_n_ = 8100 g mol^–1^, *D̵* = 1.15 at *t*_0_ and *M*_n_ = 5500 g mol^–1^, *D̵* = 1.22 at 2 h, at 50% conversion (Figures 7 and S66). This data indicates a uniform reduction in chain length (as opposed to random chain scission) and is consistent with a chain-end initiated degradation mechanism.

**Figure 7:**
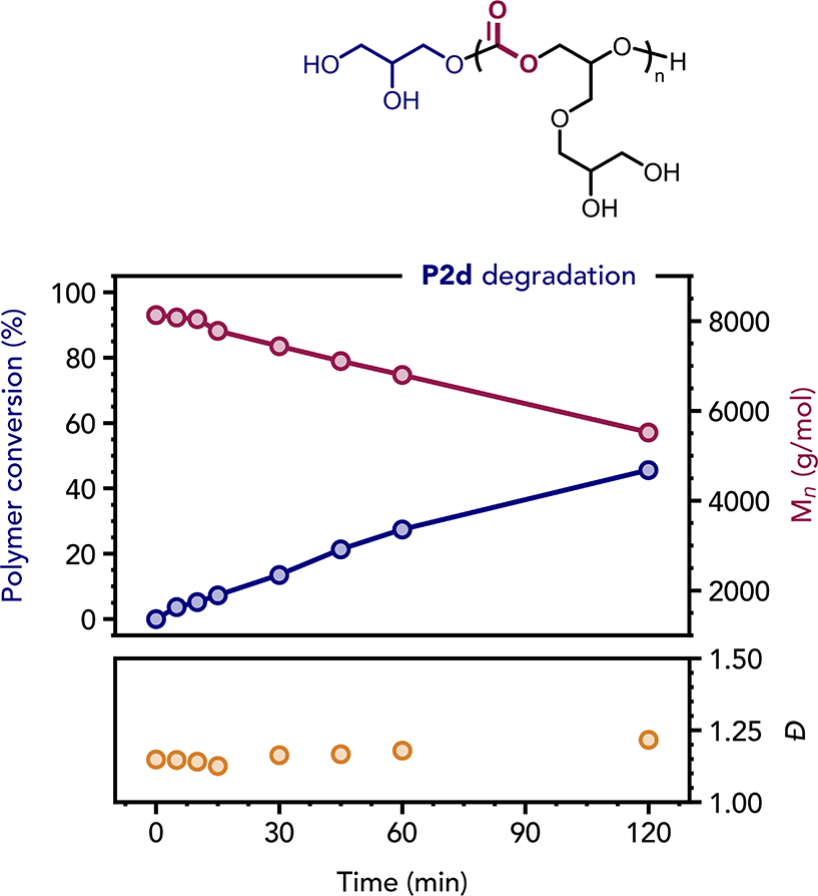
Polymer conversion (by ^1^H NMR spectroscopy), *M*_n_ and dispersity (*D̵*) (by SEC in DMF/LiBr) vs time plots for the degradation of **P2d** in water (pH 8). The SEC refractive index signal became rather weak within 2 h, preventing further reliable data collection. The complete reaction profile is reported in Figure S64B and all SEC chromatograms are shown in Figure S66.

At the end of the degradation experiment (24 h), the cyclic carbonate **2** was formed with 90% selectivity, with the remaining 10% being diglycerol. These assignments were made using ^13^C­{^1^H} NMR spectroscopy owing to signal overlap in the ^1^H NMR spectra (Figure S67). Low amounts of glycerol carbonate were also detected (Figure S68); this species is most likely formed by a second ring-closing reaction that is unique to the glycerol chain end-group in **P2d** (Scheme S2). These findings further support initiation of cyclization from the terminal hydroxyl groups (Scheme 2B).

Like the fully hydrophilic **P2d**, the stearyl-derived **P4d** remained stable for 1 month at 25 °C when stored at either neutral or acidic pH. **P4d** was then exposed to mildly alkaline aqueous solution (pH 8) and its degradation was monitored by ^1^H NMR spectroscopy. In this case, 57% degradation was observed after 8 h, and complete degradation within 24 h (Figure S69A). A satisfactory fit to the initial conversion vs time data could be obtained by assuming pseudo zero-order kinetics (Figure S69B). This approach yielded an apparent rate constant *k*_0_ of 2.02 × 10^–3^ M s^–1^, which is more than 3-fold lower than that observed for **P2d** (*k*_0_ = 6.36 × 10^–3^ M s^–1^, see Table 3). According to the chain unzipping mechanism outlined above, this rate difference can be rationalized because **P2d** contains two hydroxyl end-groups, whereas **P4d** possesses only one chain end-group (the other being an alkyl chain). In other words, depolymerization should be twice as fast for **P2d** (Figure 8). The 3-fold (instead of 2-fold) increase in *k*_0_ may arise because ring-closing is faster for primary vs secondary terminal alcohols.^46,81^ Interestingly, an opaque solution was obtained only at the end of degradation, which is attributed to the formation of water-insoluble stearyl alcohol. Although characteristic resonances for the stearyl functional group backbone (–CH_2_– and –CH_3_) were readily identified during degradation, such signals almost disappeared after 24 h. This suggests that the stearyl chain-ends are only released into the aqueous solution after polymer backbone degradation is complete, i.e. at the final carbonate linkage (Figure S70).

**Table 3:** Apparent Rate Constants Calculated for the Hydrolytic Degradation of Three Polycarbonates at 25 °C

entry	Polymer	*k*_0_ (M s^–1^)^a^	*k*_1_ (s^–1^)^b^
1	**P2d**	6.36 × 10^–3^	1.14 × 10^–4^
2	**P4d**	2.02 × 10^–3^	2.88 × 10^–5^
3	**P1cd**	n.d.^c^	1.82 × 10^–6^

a Calculated from linear fits to plots of [polymer] vs time from t_0_ up to 50% conversion.

b Calculated from linear fits to plots of ln­[polymer] vs time from t_0_ to 56–95% conversion.

c not determined (see Supporting Information for further information).

**Figure 8:**
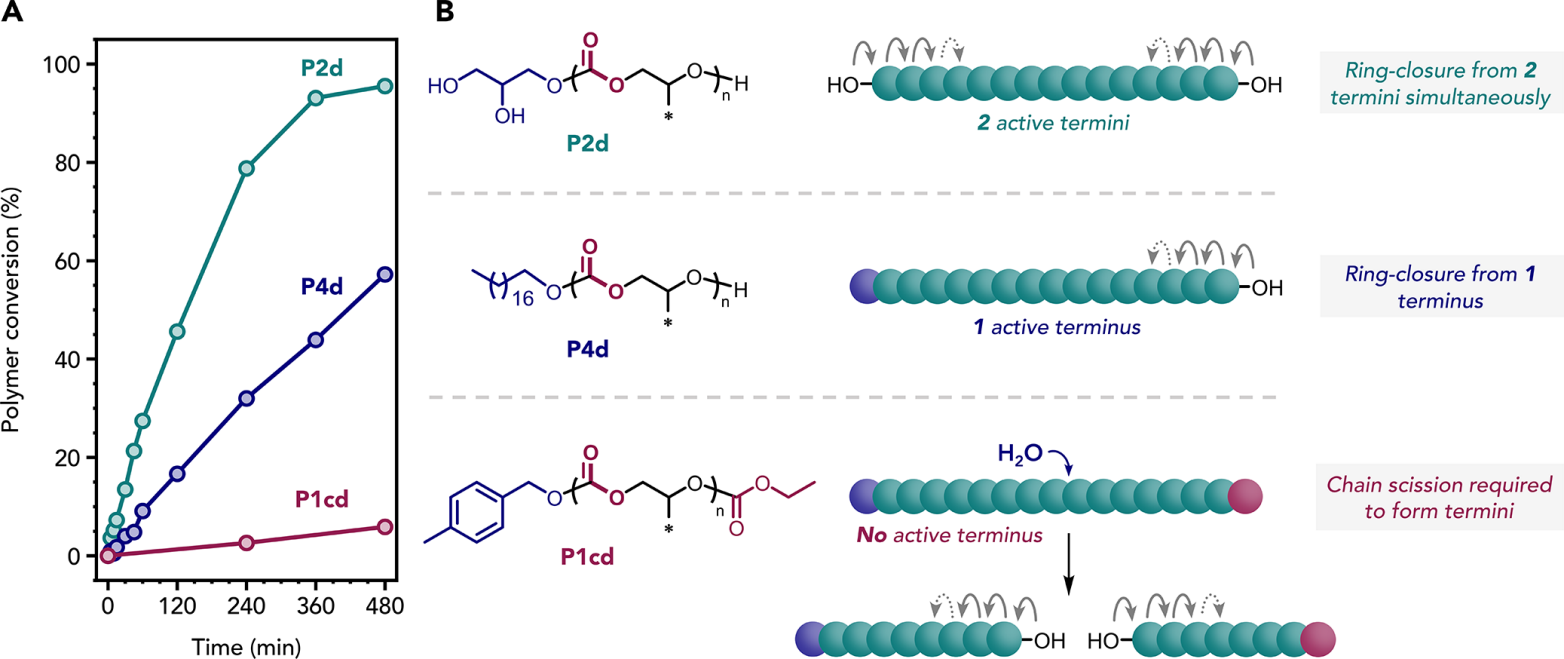
Influence of the nature of the polymer end-groups on the rate of hydrolytic degradation. (A) Conversion vs time plots obtained for the degradation of **P2d**, **P4d** and **P1cd** (pH 8, 25 °C). (B) Schematic representation of the proposed polymer degradation pathways governed by end-groups.

To further test the chain-end mechanism for the rapid degradation observed for **P2d** and **P4d**, the synthesis of **P1c**, an end-capped version of **P1**, was achieved by reaction of **P1** with ethyl chloroformate. ^1^H NMR spectroscopy studies confirmed successful functionalization via appearance of new resonances at 1.21 and 4.11 ppm that are characteristic of the ethyl carbonate group (Figure S71). Subsequent acetal deprotection yielded **P1cd** without affecting the end-groups, since the integrated signals for the MBA and ethyl carbonate end-groups were precisely as expected (Figures S72–S75). The thermal properties of the polymers, **P1c** and **P1cd**, were barely affected by such chain end-group functionalization (Figures S76 and S77).

When **P1cd** was examined under identical conditions at pH 8, its rate of degradation was substantially slower (Figure 8). Indeed, 56% conversion required 5 days while 95% conversion was only achieved after 22 days (Figure S78A). Moreover, the initial reaction kinetics (from *t*_0_ – *t*_1/2_) did not obey zero-order kinetics, suggesting a different degradation mechanism (Figure S78). In the absence of any hydroxyl termini, fast chain unzipping could not occur for **P1cd** (Figure 8). Instead, degradation required relatively slow backbone hydrolysis to generate polymer segments with free hydroxyl end-groups (Scheme 2B). These segments then undergo relatively fast chain unzipping on a shorter time scale than that required for the backbone hydrolysis. This mechanism is consistent with the formation of hydroxyl-terminated polymers via backbone hydrolysis, which is the rate-determining step for the hydrolytic degradation of end-capped polymers. The release of MBA and ethanol, the two products of end-group hydrolysis for **P1cd**, were observed in similar quantities by ^1^H NMR spectroscopy during **P1cd** degradation. Moreover, 46% of these end-groups were released for a total polymer conversion of 56% after 5 days, supporting the hypothesis of fast chain unzipping after slow chain hydrolysis (Figure S79).

As polycarbonate degradation proceeds, **2** is formed by a backbiting process. Over long reaction times, the concentration of **2** decreased, suggesting its hydrolysis to afford diglycerol and CO_2_ (Figure S78A). To verify this hypothesis, a pure sample of **2** was subjected to the same degradation conditions (pH 8) and conversion was monitored by ^1^H NMR spectroscopy over 41 days (Figure 9). Over this time period, 74% of **2** was converted into diglycerol. This observation is consistent with previous studies of the hydrolysis of glycerol carbonate in mildly alkaline aqueous solution.^81^ It is particularly encouraging that the cyclic carbonate **2** undergoes such degradation as it means that all the ultimate degradation products from these polycarbonates can be expected to be of low toxicity: (i) diglycerol is classified as nontoxic and readily biodegradable;^82^ (ii) CO_2_ was originally used to make the polymers; (iii) fatty acids and fatty alcohols, used as initiators, are naturally occurring compounds of very low toxicity, and have a widespread use in the surfactant industry.^83^ Although data on the biodegradability of diglycerol carbonate is not available, its structural similarity to glycerol carbonate, which is recognized to be biodegradable,^84^ suggests that it most likely follows a similar degradation pathway.

**Figure 9:**
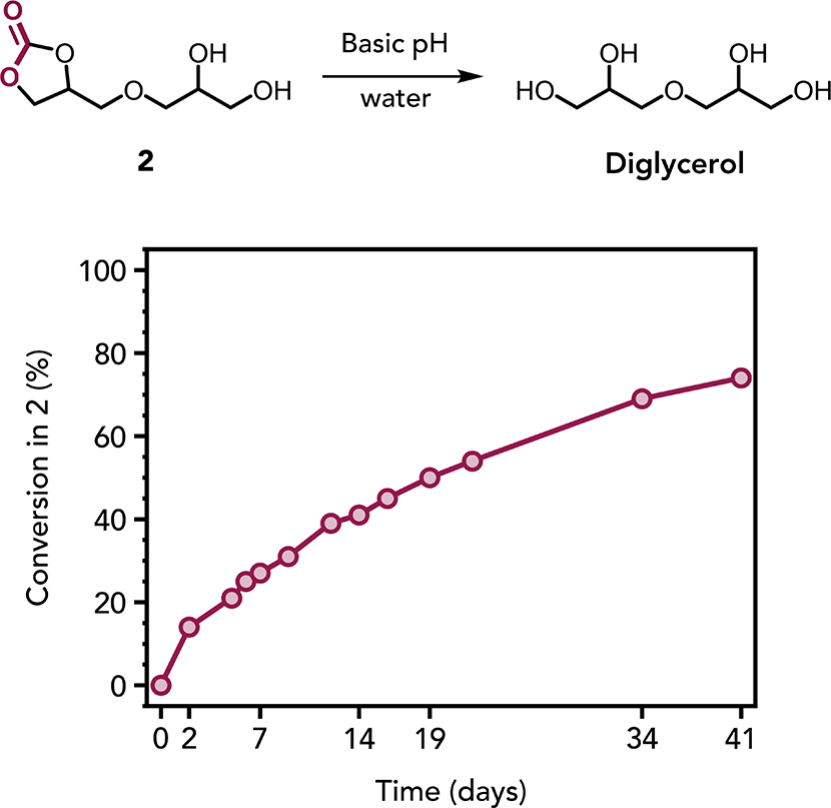
Conversion vs time plot for the degradation of **2** to form diglycerol in basic water. Reaction conditions: **2** (25 mg mL^–1^) in aqueous basic solution at pH 8 (phosphate buffer), 25 °C.

Overall, here, we have demonstrated that water-soluble polycarbonates are readily degraded under relatively mild conditions in water. The rate of degradation is strongly pH-dependent, with high stability being observed at either acidic or neutral pH, while either slow or rapid degradation occurs as the pH is raised from 8 to 10. The degradation mechanism involves a chain-end initiated backbiting mechanism to form cyclic carbonate. The degradation time scale can be tuned via end-group modification, whereby hydrolysis of the carbonate linkages becomes rate-determining.

Although chain unzipping via cyclic carbonate formation is known for ROCOP-derived polycarbonates, this reaction typically requires both a high temperature and a catalyst.^58,85^ Our results suggest that aqueous solvation of the hydrophilic polycarbonate substantially reduces the activation energy required for backbiting, thereby enabling rapid depolymerization in water.

## Conclusion

The controlled copolymerization of diglycerol-derived epoxide and CO_2_ to produce well-defined polycarbonates was achieved using a [Co­(III)/K­(I)] heterodinuclear catalyst which showed very good control over the polycarbonate molecular weight, low dispersity, and well-defined chain-end group chemistry. Deprotection of the polycarbonate acetal substituents yielded water-soluble polycarbonates bearing pendant diols functionalities. Employing different initiators in the catalysis produced a series of hydrophilic and amphiphilic polycarbonates, providing access to spontaneous self-assembly when dissolving the polymers in water and suggesting they could show future benefits as polymer surfactants. Importantly, the polycarbonates are highly stable in neutral or acidic aqueous solutions but undergo complete degradation in alkaline aqueous solutions to form known compounds of low toxicity, including diglycerol and carbon dioxide. The rate of hydrolytic degradation depends on the polycarbonate structure, with time scales varying from a few hours to up to one month. Overall, this study introduces a promising new polycarbonate-based platform technology for the rational design of inherently degradable hydrophilic or amphiphilic polymers prepared from renewable carbon sources. These polycarbonates may be more sustainable alternatives to conventional fossil-based and nondegradable polymers that are currently used in many liquid formulations. These diglycerol and carbon dioxide-derived polycarbonates should be prioritized for use in controlled release and liquid formulation applications.

## Supplementary Material



## Notes

The open-access experimental data can be found via the DOI 10.5287/ora-g29ybxgq7. The Supporting Information is available free of charge at https://pubs.acs.org/doi/10.1021/jacs.5c20433.

Materials, instrumentation, synthetic procedures, NMR spectra of synthesized molecules and polymers, SEC data, MALDI-TOF, thermal data by TGA and DSC, IR spectra of polymerization kinetics and pure polymers, rheological analyses, additional CryoTEM images, NMR spectra supporting the degradation study (PDF)

## References

[1] Hepburn C., Adlen E., Beddington J., Carter E. A., Fuss S., Mac Dowell N., Minx J. C., Smith P., Williams C. K. (2019). The technological
and economic prospects for CO2 utilization and removal. Nature.

[2] Artz J., Müller T. E., Thenert K., Kleinekorte J., Meys R., Sternberg A., Bardow A., Leitner W. (2018). Sustainable
Conversion of Carbon Dioxide: An Integrated Review of Catalysis and
Life Cycle Assessment. Chem. Rev..

[3] Vidal F., van der Marel E. R., Kerr R. W. F., McElroy C., Schroeder N., Mitchell C., Rosetto G., Chen T. T. D., Bailey R. M., Hepburn C., Redgwell C., Williams C. K. (2024). Designing a circular
carbon and plastics economy for a sustainable future. Nature.

[4] Grignard B., Gennen S., Jérôme C., Kleij A. W., Detrembleur C. (2019). Advances in the use of CO2 as a renewable
feedstock
for the synthesis of polymers. Chem. Soc. Rev..

[5] Liu Y., Lu X.-B. (2023). Current Challenges
and Perspectives in CO2-Based Polymers. Macromolecules.

[6] Paul S., Zhu Y., Romain C., Brooks R., Saini P. K., Williams C. K. (2015). Ring-opening
copolymerization (ROCOP): synthesis and properties of polyesters and
polycarbonates. Chem. Commun..

[7] Wang Y., Darensbourg D. J. (2018). Carbon
dioxide-based functional polycarbonates: Metal
catalyzed copolymerization of CO2 and epoxides. Coord. Chem. Rev..

[8] Tang S., Lin B.-L., Tonks I., Eagan J. M., Ni X., Nozaki K. (2024). Sustainable Copolymer
Synthesis from Carbon Dioxide
and Butadiene. Chem. Rev..

[9] Xia Y., Zhang C., Zhang X. (2025). Making polymers
with low carbon content:
a sustainable option. Green Chem..

[10] Lidston C. A. L., Severson S. M., Abel B. A., Coates G. W. (2022). Multifunctional
Catalysts for Ring-Opening Copolymerizations. ACS Catal..

[11] Della
Monica F., Kleij A. W. (2020). From terpenes to sustainable and
functional polymers. Polym. Chem..

[12] Siragusa F., Detrembleur C., Grignard B. (2023). The advent of recyclable CO2-based
polycarbonates. Polym. Chem..

[13] Diment W. T., Lindeboom W., Fiorentini F., Deacy A. C., Williams C. K. (2022). Synergic
Heterodinuclear Catalysts for the Ring-Opening Copolymerization (ROCOP)
of Epoxides, Carbon Dioxide, and Anhydrides. Acc. Chem. Res..

[14] Darensbourg D. J. (2019). Chain transfer
agents utilized in epoxide and CO2 copolymerization processes. Green Chem..

[15] Scharfenberg M., Hilf J., Frey H. (2018). Functional Polycarbonates from Carbon
Dioxide and Tailored Epoxide Monomers: Degradable Materials and Their
Application Potential. Adv. Funct. Mater..

[16] Yang G.-W., Xie R., Zhang Y.-Y., Xu C.-K., Wu G.-P. (2024). Evolution of Copolymers
of Epoxides and CO2: Catalysts, Monomers, Architectures, and Applications. Chem. Rev..

[17] Li H., Wang W., Liu S., Xue D., Wang J., Liu Y., Huang Q. (2024). Design and syntheses of functional carbon dioxide-based
polycarbonates via ternary copolymerization. J. CO2 Util..

[18] Deacy A. C., Gregory G. L., Sulley G. S., Chen T. T. D., Williams C. K. (2021). Sequence
Control from Mixtures: Switchable Polymerization Catalysis and Future
Materials Applications. J. Am. Chem. Soc..

[19] Sulley G. S., Gregory G. L., Chen T. T. D., Peña Carrodeguas L., Trott G., Santmarti A., Lee K.-Y., Terrill N. J., Williams C. K. (2020). Switchable Catalysis
Improves the Properties of CO2-Derived
Polymers: Poly­(cyclohexene carbonate-b-ε-decalactone-b-cyclohexene
carbonate) Adhesives, Elastomers, and Toughened Plastics. J. Am. Chem. Soc..

[20] Cao H., Wang X. (2021). Carbon dioxide copolymers: Emerging sustainable materials for versatile
applications. SusMat.

[21] Zumstein M., Battagliarin G., Kuenkel A., Sander M. (2022). Environmental Biodegradation
of Water-Soluble Polymers: Key Considerations and Ways Forward. Acc. Chem. Res..

[22] Picken C. A. R., Buensoz O., Price P. D., Fidge C., Points L., Shaver M. P. (2023). Sustainable formulation polymers
for home, beauty and
personal care: challenges and opportunities. Chem. Sci..

[23] Kelly C. L. (2023). Addressing
the sustainability challenges for polymers in liquid formulations. Chem. Sci..

[24] Vandermeulen G. W. M., Boarino A., Klok H.-A. (2022). Biodegradation of water-soluble and
water-dispersible polymers for agricultural, consumer, and industrial
applications—Challenges and opportunities for sustainable materials
solutions. J. Polym. Sci..

[25] Polymers in liquid formulations: opportunities for a sustainable future; 2021. https://www.rsc.org/policy-and-campaigning/policy-library/polymers-in-liquid-formulation-opportunities-for-a-sustainable-future (accessed Aug 13 2025).

[26] Blanazs A., Ryan A. J., Armes S. P. (2012). Predictive
Phase Diagrams for RAFT
Aqueous Dispersion Polymerization: Effect of Block Copolymer Composition,
Molecular Weight, and Copolymer Concentration. Macromolecules.

[27] Cumming J. M., Deane O. J., Armes S. P. (2022). Reversible Addition-Fragmentation
Chain Transfer Aqueous Dispersion Polymerization of 4-Hydroxybutyl
Acrylate Produces Highly Thermoresponsive Diblock Copolymer Nano-Objects. Macromolecules.

[28] Sponchioni M., O’Brien C. T., Borchers C., Wang E., Rivolta M. N., Penfold N. J. W., Canton I., Armes S. P. (2020). Probing
the mechanism
for hydrogel-based stasis induction in human pluripotent stem cells:
is the chemical functionality of the hydrogel important?. Chem. Sci..

[29] Ellis C. E., Garcia-Hernandez J. D., Manners I. (2022). Scalable and Uniform Length-Tunable
Biodegradable Block Copolymer Nanofibers with a Polycarbonate Core
via Living Polymerization-Induced Crystallization-Driven Self-assembly. J. Am. Chem. Soc..

[30] MacFarlane L., Zhao C., Cai J., Qiu H., Manners I. (2021). Emerging applications
for living crystallization-driven self-assembly. Chem. Sci..

[31] Yu Q., Roberts M. G., Pearce S., Oliver A. M., Zhou H., Allen C., Manners I., Winnik M. A. (2019). Rodlike Block Copolymer
Micelles of Controlled Length in Water Designed for Biomedical Applications. Macromolecules.

[32] Georgiou P. G., Neal T. J., Newell M. A., Hepburn K. S., Tyler J. J. S., Farmer M. A. H., Chohan P., Roth P. J., Nicolas J., Armes S. P. (2025). Degradable Diblock Copolymer Vesicles
via Radical Ring-Opening
Polymerization-Induced Self-Assembly in Aqueous Media. J. Am. Chem. Soc..

[33] Farmer M. A. H., Musa O. M., Armes S. P. (2024). Combining Crystallization-Driven
Self-Assembly with Reverse Sequence Polymerization-Induced Self-Assembly
Enables the Efficient Synthesis of Hydrolytically Degradable Anisotropic
Block Copolymer Nano-objects Directly in Concentrated Aqueous Media. J. Am. Chem. Soc..

[34] Arp H. P. H., Knutsen H. (2020). Could We Spare a Moment of the Spotlight for Persistent,
Water-Soluble Polymers?. Environ. Sci. Technol..

[35] Zhu C., Nicolas J. (2022). (Bio)­degradable and
Biocompatible Nano-Objects from
Polymerization-Induced and Crystallization-Driven Self-Assembly. Biomacromolecules.

[36] Guerassimoff L., Cao J., Auguste M., Bossion A., Zhu C., Le D., Cailleau C., Ismail S. M., Mercier-Nomé F., Nicolas J. (2025). Degradable water-soluble polymer prodrugs for subcutaneous
delivery of irritant anticancer drugs. Chem.
Sci..

[37] Bingham N.
M., Roth P. J. (2019). Degradable
vinyl copolymers through thiocarbonyl addition–ring-opening
(TARO) polymerization. Chem. Commun..

[38] Smith R. A., Fu G., McAteer O., Xu M., Gutekunst W. R. (2019). Radical
Approach to Thioester-Containing Polymers. J.
Am. Chem. Soc..

[39] Hauenstein O., Agarwal S., Greiner A. (2016). Bio-based polycarbonate as synthetic
toolbox. Nat. Commun..

[40] Wang Y., Fan J., Darensbourg D. J. (2015). Construction of Versatile and Functional
Nanostructures Derived from CO2-based Polycarbonates. Angew. Chem., Int. Ed..

[41] Darensbourg D. J., Tsai F.-T. (2014). Postpolymerization Functionalization of Copolymers
Produced from Carbon Dioxide and 2-Vinyloxirane: Amphiphilic/Water-Soluble
CO2-Based Polycarbonates. Macromolecules.

[42] Deng K., Wang S., Ren S., Han D., Xiao M., Meng Y. (2016). A Novel Single-Ion-Conducting Polymer
Electrolyte Derived from CO2-Based
Multifunctional Polycarbonate. ACS Appl. Mater.
Interfaces.

[43] Darensbourg D. J., Chung W.-C., Arp C. J., Tsai F.-T., Kyran S. J. (2014). Copolymerization
and Cycloaddition Products Derived from Coupling Reactions of 1,2-Epoxy-4-cyclohexene
and Carbon Dioxide. Postpolymerization Functionalization via Thiol–Ene
Click Reactions. Macromolecules.

[44] Wei J., Ma Y., Cai Y., Zheng J., Fan H. (2022). Synthesis of high-toughness
waterborne polyurethane utilizing self-emulsifying CO2-based polyols. Prog. Org. Coat..

[45] Jia M., Zhang D., Gnanou Y., Feng X. (2021). Surfactant-Emulating
Amphiphilic Polycarbonates and Other Functional Polycarbonates through
Metal-Free Copolymerization of CO2 with Ethylene Oxide. ACS Sustainable Chem. Eng..

[46] Zhang H., Grinstaff M. W. (2013). Synthesis
of Atactic and Isotactic Poly­(1,2-glycerol
carbonate)­s: Degradable Polymers for Biomedical and Pharmaceutical
Applications. J. Am. Chem. Soc..

[47] Geschwind J., Frey H. (2013). Poly­(1,2-glycerol carbonate):
A Fundamental Polymer Structure Synthesized
from CO2 and Glycidyl Ethers. Macromolecules.

[48] Song P., Shang Y., Chong S., Zhu X., Xu H., Xiong Y. (2015). Synthesis and characterization of
amino-functionalized poly­(propylene
carbonate). RSC Adv..

[49] Zhang H., Lin X., Chin S., Grinstaff M. W. (2015). Synthesis and Characterization of
Poly­(glyceric Acid Carbonate): A Degradable Analogue of Poly­(acrylic
Acid). J. Am. Chem. Soc..

[50] Liu Y., Yu Y., Zhang Q.-X., Song T.-T., Lu X.-B. (2025). Amphiphilic
and
Chemically Recyclable CO2-Based Polycarbonates from Biosourced Epoxides. Macromolecules.

[51] Chiellini E., Bemporad L., Solaro R. (1994). Novel Hydroxyl Containing
Polyesters
and Polycarbonates by the Copolymerization of Glycidyl Ethers of Protected
Alditols and Cyclic Anhydrides. J. Bioact. Compat.
Polym..

[52] Geschwind J., Frey H. (2013). Stable, Hydroxyl Functional
Polycarbonates With Glycerol Side Chains
Synthesized From CO2 and Isopropylidene­(glyceryl glycidyl ether). Macromol. Rapid Commun..

[53] Lu X.-Y., Zhang R.-S., Yang G.-W., Li Q., Li B., Wu G.-P. (2024). Aqueous Developable and CO2-Sourced Chemical Amplification Photoresist
with High Performance. Angew. Chem., Int. Ed..

[54] Liu Y., Wang M., Ren W.-M., He K.-K., Xu Y.-C., Liu J., Lu X.-B. (2014). Stereospecific
CO2 Copolymers from 3,5-Dioxaepoxides:
Crystallization and Functionallization. Macromolecules.

[55] Doncom K. E. B., Blackman L. D., Wright D. B., Gibson M. I., O’Reilly R. K. (2017). Dispersity
effects in polymer self-assemblies: a matter of hierarchical control. Chem. Soc. Rev..

[56] Inoue S. (2000). Immortal polymerization:
The outset, development, and application. J.
Polym. Sci., Part A: Polym. Chem..

[57] Deacy A. C., Moreby E., Phanopoulos A., Williams C. K. (2020). Co­(III)/Alkali-Metal­(I)
Heterodinuclear Catalysts for the Ring-Opening Copolymerization of
CO(2) and Propylene Oxide. J. Am. Chem. Soc..

[58] Deacy A. C., Phanopoulos A., Lindeboom W., Buchard A., Williams C. K. (2022). Insights
into the Mechanism of Carbon Dioxide and Propylene Oxide Ring-Opening
Copolymerization Using a Co­(III)/K­(I) Heterodinuclear Catalyst. J. Am. Chem. Soc..

[59] Borke T., Korpi A., Pooch F., Tenhu H., Hietala S. (2017). Poly­(glyceryl
glycerol): A multi-functional hydrophilic polymer for labeling with
boronic acids. J. Polym. Sci., Part A: Polym.
Chem..

[60] Mangold C., Wurm F., Obermeier B., Frey H. (2010). “Functional
Poly­(ethylene glycol)”: PEG-Based Random Copolymers with 1,2-Diol
Side Chains and Terminal Amino Functionality. Macromolecules.

[61] Hofmann A. M., Wurm F., Frey H. (2011). Rapid Access to Polyfunctional Lipids
with Complex Architecture via Oxyanionic Ring-Opening Polymerization. Macromolecules.

[62] Kuo S.-W., Tsai H.-T. (2009). Complementary Multiple Hydrogen-Bonding Interactions
Increase the Glass Transition Temperatures to PMMA Copolymer Mixtures. Macromolecules.

[63] Lewis C. L., Stewart K., Anthamatten M. (2014). The Influence of Hydrogen Bonding
Side-Groups on Viscoelastic Behavior of Linear and Network Polymers. Macromolecules.

[64] Shabbir A., Goldansaz H., Hassager O., van Ruymbeke E., Alvarez N. J. (2015). Effect of Hydrogen Bonding on Linear and Nonlinear
Rheology of Entangled Polymer Melts. Macromolecules.

[65] Golkaram M., Fodor C., van Ruymbeke E., Loos K. (2018). Linear Viscoelasticity
of Weakly Hydrogen-Bonded Polymers near and below the Sol–Gel
Transition. Macromolecules.

[66] Barnes, H. A. A Handbook of Elementary Rheology; University of Wales, Institute of Non-Newtonian Fluid Mechanics, 2000.

[67] Ricarte R. G., Shanbhag S. (2024). A tutorial review of
linear rheology for polymer chemists:
basics and best practices for covalent adaptable networks. Polym. Chem..

[68] Hilf J., Schulze P., Frey H. (2013). CO2-Based Non-ionic Surfactants:
Solvent-Free Synthesis of Poly­(ethylene glycol)-block-Poly­(propylene
carbonate) Block Copolymers. Macromol. Chem.
Phys..

[69] Huang Z., Wang Y., Zhang N., Zhang L., Darensbourg D. J. (2018). One-Pot
Synthesis of Ion-Containing CO2-Based Polycarbonates Using Protic
Ionic Liquids as Chain Transfer Agents. Macromolecules.

[70] Fitzgerald D. M., Zhang H., Bordeianu C., Colson Y. L., Grinstaff M. W. (2023). Synthesis
of Polyethylene Glycol–Poly­(glycerol carbonate) Block Copolymeric
Micelles as Surfactant-Free Drug Delivery Systems. ACS Macro Lett..

[71] Jennings J., Butler M. F., McLeod M., Csányi E., Ryan A. J., Mykhaylyk O. O. (2018). Stearyl Methacrylate-Based Polymers
as Crystal Habit Modifiers for Triacylglycerols. Cryst. Growth Des..

[72] Webber S. E. (1998). Polymer
Micelles: An Example of Self-Assembling Polymers. J. Phys. Chem. B.

[73] Zhu Y., Yang B., Chen S., Du J. (2017). Polymer vesicles: Mechanism,
preparation, application, and responsive behavior. Prog. Polym. Sci..

[74] Lefley J., Waldron C., Becer C. R. (2020). Macromolecular
design and preparation
of polymersomes. Polym. Chem..

[75] Jena G., Dutta K., Daverey A. (2023). Surfactants
in water and wastewater
(greywater): Environmental toxicity and treatment options. Chemosphere.

[76] Palmer M., Hatley H. (2018). The role of surfactants
in wastewater treatment: Impact,
removal and future techniques: A critical review. Water Res..

[77] Takanashi M., Nomura Y., Yoshida Y., Inoue S. (1982). Functional polycarbonate
by copolymerization of carbon dioxide and epoxide: Synthesis and hydrolysis. Makromol. Chem..

[78] Beharaj A., Ekladious I., Grinstaff M. W. (2019). Poly­(Alkyl
Glycidate Carbonate)­s
as Degradable Pressure-Sensitive Adhesives. Angew. Chem., Int. Ed..

[79] McBride R. A., Gillies E. R. (2013). Kinetics of Self-Immolative Degradation in a Linear
Polymeric System: Demonstrating the Effect of Chain Length. Macromolecules.

[80] Chen E. K. Y., McBride R. A., Gillies E. R. (2012). Self-Immolative Polymers Containing
Rapidly Cyclizing Spacers: Toward Rapid Depolymerization Rates. Macromolecules.

[81] Chen F., Qi R., Huyer L. D., Amsden B. G. (2018). Degradation of poly­(5-hydroxy-trimethylene
carbonate) in aqueous environments. Polym. Degrad.
Stab..

[82] ECHACHEM database, Oxybispropanediol, dossier registered 11-Oct-2012. https://chem.echa.europa.eu/(accessed Aug 13 2025).

[83] Noweck, K. ; Grafahrend, W. Fatty Alcohols. In Ullmann’s Encyclopedia of Industrial Chemistry; John Wiley & Sons, Ltd, 2006.

[84] ECHACHEM database, 4-hydroxymethyl-1,3-dioxolan-2-one, dossier registered 10-Jul-2014. https://chem.echa.europa.eu/(accessed Aug 13 2025).

[85] Darensbourg D. J., Wei S.-H. (2012). Depolymerization of Polycarbonates
Derived from Carbon
Dioxide and Epoxides to Provide Cyclic Carbonates. A Kinetic Study. Macromolecules.

