# Supplementary material for: Controlled Polymerization Catalysis for the Synthesis of Degradable Amphiphilic Polycarbonates from CO2
Source: J Am Chem Soc. 2026 Feb 12;148(7):7426–39. doi: 10.1021/jacs.5c20433 (PMC12951427; doi:10.1021/jacs.5c20433)
Supplement: Supplementary file 1 [file ja5c20433_si_001.pdf]

Supporting Information

Controlled Polymerization Catalysis for the Synthesis of Degradable Amphiphilic Polycarbonates from CO_2

Diego A. Resendiz-Lara‡ [a], Thomas Habets‡ [a], Steven P. Armes [b], Charlotte K. Williams [a*]

[a] Chemistry Research Laboratory, Department of Chemistry, University of Oxford, 12 Mansfield Road, Oxford, UK, OX1 3TA.

[b] School of Mathematics and Physical Sciences, University of Sheffield, Dainton Building, Brook Hill, Sheffield, South Yorkshire S3 7HF, U.K.

* charlotte.williams@chem.ox.ac.uk

‡ D.A.R.L. and T.H. contributed equally to this work.

The experimental data can be found via the DOI 10.5287/ora-g29ybxgq7.

Contents

Materials and Instrumentation **6**

Materials 6

Instrumentation 7

Experimental procedures **9**

Synthesis and purification of IGG 9

Representative Polymerization of **IGG** and CO_2 into **P1** using MBA as initiator 9

Representative acetal deprotection strategies 11

Synthesis of **P2**-**P6** 11

Synthesis of **1** and **2** 11

Representative methodology for the monitoring of degradation 12

Synthesis of P1c 13

Methodology for the monitoring of degradation of 2 13

ROCOP mechanism **15**

Scheme S 1 – Key steps of the ROCOP of CO_2 and epoxides. 15

Characterization of IGG **16**

Figure S 1 – ^{1}H-NMR spectrum (400 MHz, DMSO-d_6) of **IGG**. 16

Figure S 2 – ^{13}C{^{1}H}-NMR spectrum (101 MHz, DMSO-d_6) of **IGG**. 16

Synthesis and characterization of P1 .... **17**

Figure S 3 – Typical ^{1}H-NMR spectrum (400 MHz, $CDCl_3$) of a crude reaction medium. 17

Figure S 4 – In-situ IR spectra monitoring used for the ROCOP of CO_2 and **IGG** at 25 °C (Table 1, entry 4). 17

Figure S 5 – ^{1}H-NMR spectrum (600 MHz, $CDCl_3$) of **P1**. 18

Figure S 6 – ^{13}C{^{1}H}-NMR spectrum (151 MHz, $CDCl_3$) of **P1**. 18

Figure S 7 – Stacked ^{1}H-NMR spectra (400 MHz, $CDCl_3$) of derivatives of **P1** with varied degree of polymerization (DP). 19

Figure S 8 – TGA data of **P1** samples with varied degree of polymerization (DP). 20

Figure S 9 – Stacked DSC data for **P1** samples with varied degree of polymerization (DP). 20

Deprotection of acetal in P1 and characterization of P1d **21**

Figure S 10 – (A) Stacked ^{1}H-NMR spectra (400 MHz, DMSO-d_6) of the crude reaction medium for the deprotection of **P1** into **P1d**, following protocol **B**. Over time (from bottom to top), the typical resonance of the polymer at 4.97 ppm remains intact, while the resonances attributed to the acetal groups at 1.24 and 1.29 ppm disappeared. (B) Plot of acetal conversion *vs* time for the deprotection of **P1**. 21

Figure S 11 – ^{1}H-NMR spectrum (400 MHz, DMSO-d_6) of **P1d**. 21

Figure S 12 – ^{13}C{^{1}H}-NMR spectrum (151 MHz, DMSO-d_6) of **P1d**. 22

Figure S 13 – (A) MALDI-TOF spectrum of **P1d** (DP 20). (B) Plot of *m/z* vs Nth repeat unit. The theoretical molecular weights of the repeating unit and the end group match the experimental values. 22

Figure S 14 – Zoomed ATR-IR spectra showing the C=O band for **P1** (blue) and **P1d** (green) (DP = 20). 23

Figure S 15 – TGA (left) and DSC (right) data for **P1d** (DP = 20). 23

Rheological analyses of P1 and P1d **24**

Figure S 16 – Rheological analyses for samples of **P1**: (A,B) Amplitude sweeps (frequency = 2 Hz) at 20 and 70 °C. (C) Temperature sweep (frequency = 2 Hz; strain = 5 %). 24

Figure S 17 – Rheological analyses for samples of **P1d**: (A,B) Amplitude sweeps (frequency = 2 Hz) at 20 and 60 °C. (C) Temperature sweep (frequency = 2 Hz; strain = 0.5 %).......... 25

Figure S 18 – Rheological analyses for **P1**: Frequency sweeps (strain = 5 %) from 20 to 50 °C. 26

Figure S 19 – Rheological analyses for **P1d**: Frequency sweeps (strain = 0.5 %) from 20 to 50 °C. 27

Characterization of P2-6 and P2d-6d.......... 28

Figure S 20 – ^{1}H-NMR spectrum (400 MHz, DMSO-d_6) of **P2**.......... 28

Figure S 21 – ^{13}C{^{1}H}-NMR spectrum (151 MHz, DMSO-d_6) of **P2**.......... 28

Figure S 22 – SEC trace (in THF) of **P2**. 28

Figure S 23 – ^{1}H-NMR spectrum (600 MHz, DMSO-d_6) of **P2d**. 29

Figure S 24 – ^{13}C{^{1}H}-NMR spectrum (151 MHz, DMSO-d_6) of **P2d**. 29

Figure S 25 – Stacked SEC traces (in DMF) of **P2** (top) and **P2d** (bottom) in term of molar mass (left) or retention time (right). 29

Figure S 26 – TGA data for **P2** and **P2d**. 30

Figure S 27 – DSC data for **P2** and **P2d**.......... 30

Figure S 28 – Plot of **IGG** conversion *vs* time for **P1** and **P3** using in-situ IR spectroscopy monitoring. 30

Figure S 29 – Stacked SEC traces (in DMF) of **P3** (top) and **P3d** (bottom). 31

Figure S 30 – ^{1}H-NMR spectrum (500 MHz, $CDCl_3$) of **P3**. 31

Figure S 31 – ^{13}C{^{1}H}-NMR spectrum (126 MHz, $CDCl_3$) of **P3**. 31

Figure S 32 – (A) MALDI-TOF spectrum of **P3**. (B) Plot of *m/z* vs Nth repeat unit. The theoretical molecular weights of the repeating unit and the end group match the experimental values. 32

Figure S 33 – ^{1}H-NMR spectrum (500 MHz, DMSO-d_6) of **P3d**. 32

Figure S 34 – ^{13}C{^{1}H}-NMR spectrum (126 MHz, DMSO-d_6) of **P3d**. 33

Figure S 35 – ^{1}H-NMR spectrum (600 MHz, DMSO-d_6) of **P4**.......... 33

Figure S 36 – ^{13}C{^{1}H}-NMR spectrum (151 MHz, DMSO-d_6) of **P4**.......... 33

Figure S 37 – ^{1}H-NMR spectrum (600 MHz, DMSO-d_6) of **P4d**. 34

Figure S 38 – ^{13}C{^{1}H}-NMR spectrum (151 MHz, DMSO-d_6) of **P4d**. 34

Figure S 39 – Stacked SEC traces (in DMF) of **P4** (top) and **P4d** (bottom). 34

Figure S 40 – ^{1}H-NMR spectrum (500 MHz, $CDCl_3$) of **P5**. 35

Figure S 41 – ^{13}C{^{1}H}-NMR spectrum (126 MHz, $CDCl_3$) of **P5**. 35

Figure S 42 – (A) MALDI-TOF spectrum of **P5**. (B) Plot of *m/z* vs Nth repeat unit. The theoretical molecular weights of the repeating unit and the end group match the experimental values. 36

Figure S 43 – ^{1}H-NMR spectrum (500 MHz, DMSO-d_6) of **P5d**. 36

Figure S 44 – ^{13}C{^{1}H}-NMR spectrum (126 MHz, DMSO-d_6) of **P5d**. 37

Figure S 45 – Stacked SEC traces (in DMF) of **P5** (top) and **P5d** (bottom). 37

Figure S 46 – ^{1}H-NMR spectrum (600 MHz, $CDCl_3$) of **P6**. 38

Figure S 47 – ^{13}C{^{1}H}-NMR spectrum (151 MHz, $CDCl_3$) of **P6**. 38

Figure S 48 – ^{1}H-NMR spectrum (700 MHz, DMSO-d_6) of **P6d**. 39

Figure S 49 – ^{13}C{^{1}H}-NMR spectrum (176 MHz, DMSO-d_6) of **P6d**. 39

Figure S 50 – Stacked SEC traces (in DMF) of **P6** (top) and **P6d** (bottom). 39

Figure S 51 – TGA data for **P3**, **P4**, **P5** and **P6**. 40

Figure S 52 – DSC data for **P3**, **P4**, **P5** and **P6**. 40

Figure S 53 – TGA data for **P3d**, **P4d**, **P5d** and **P6d**. 41

Figure S 54 – DSC data for **P3d**, **P4d**, **P5d** and **P6d**. 41

CryoTEM imaging of P4d 42

Figure S 55 – (A) DLS particle size distribution recorded for a 10 mg mL^{-1} aqueous solutions of the polymer **P4d** (same as in Figure 5). A CryoTEM image of the same solution of **P4d** shows rod-like assemblies, certainly responsible for the second minor population of larger particles with a hydrodynamic sphere-equivalent diameter of 215 nm detected by DLS. (B) Additional images from the same CryoTEM grid. The rods are characterized by average lengths of 70–230 nm and mean diameters of 6–13 nm. 42

Surface tensiometry of P4d 43

Figure S 56 – Aqueous surface tension vs. concentration plots obtained for polymer **P4d**. The raw data was fitted to a 5-parameter logistic function. 43

Degradation study and characterization of compounds 1 and 2, and polymers P1c and P1cd 44

Figure S 57 – Stacked ^{1}H-NMR spectra (400 MHz, D_2O) of a 25 mg mL^{-1} solution of **P2d** dissolved in neutral D_2O (top) and acidified D_2O by addition of 1 % (v/v) D_2SO_4 (bottom). DMSO-H_6 was added to the acidic solution to track degradation. No changes were observed in both cases after one month. 44

Figure S 58 – Stacked ^{1}H-NMR spectra (400 MHz, D_2O) of the crude reaction medium at t_0 (top) and 10 min (bottom) for the degradation of **P2d** at pH 10. 44

Figure S 59 – ^{1}H-NMR spectrum (400 MHz, DMSO-d_6) of **1**. 45

Figure S 60 – ^{13}C{^{1}H}-NMR spectrum (101 MHz, DMSO-d_6) of **1**. 45

Figure S 61 – ^{1}H-NMR spectrum (400 MHz, DMSO-d_6) of **2**. 46

Figure S 62 – ^{13}C{^{1}H}-NMR spectrum (101 MHz, DMSO-d_6) of **2**. 46

Figure S 63 – (A) Representative ^{1}H-NMR spectrum (400 MHz, D_2O) of the crude reaction medium (at t = 2h) for the degradation of **P2d** at pH 8. (B) Peak fitting was achieved on the well-defined triplet (4.66 ppm) to get accurate integration. 47

Figure S 64 – (A) Stacked ^{1}H-NMR spectra (400 MHz, D_2O) of the crude reaction medium for the degradation of **P2d** at pH 8. Over time (from bottom to top), the typical resonance of the polymer (5.14 ppm) disappears while typical resonances of product **2** appear (5.07 and 4.66 ppm). (B) Plot of the polymer linkage conversion *vs* time for the degradation of **P2d**. 47

Figure S 65 – Plots used to monitor the kinetics of degradation of **P2d**: (Left) Plot of the polymer linkage conversion *vs* time until half conversion. A zero-order was observed in this regime and a rate constant k_{zero} could be extracted from a linear fit. (Right) Plot of ln([Polymer]) *vs* time over a broader timescale. A first-order dependence on polymer concentration was observed and a rate constant k_{first} could be extracted from a linear fit. 48

Figure S 66 – Stacked SEC traces (in DMF) for the degradation of **P2d** along time (pH 8). 48

Figure S 67 – ^{13}C{^{1}H}-NMR spectrum (151 MHz, D_2O) of the crude reaction medium at the end of degradation of **P2d**. 49

Figure S 68 – Zoomed ^{1}H-NMR spectrum (400 MHz, D_2O) of the crude reaction medium (at t = 24h) for the degradation of **P2d** at pH 8. A small peak corresponding to glycerol carbonate is visible close to the peak corresponding to **2**. 49

Scheme S 2 – The degradation of **P2d** leads to two cyclic carbonates. A first ring-closure (i) from the hydroxyl end -group leads to **2**, and a second ring-closure (ii) from the hydroxyl of the deprotected solketal chain-end leads to glycerol carbonate. 50

Figure S 69 – Plots used to monitor the kinetics of degradation of **P4d**: (A) Complete plot of the polymer linkage conversion *vs* time for the degradation of **P4d**. (B) Plot of the polymer linkage conversion *vs* time until half conversion. A zero-order was observed in this regime and a rate constant k_{zero} could be extracted

from a linear fit. (C) Plot of ln([Polymer]) *vs* time over a broader timescale. A first-order dependence on polymer concentration was observed and a rate constant k_{first} could be extracted from a linear fit. 50

Figure S 70 – (A) Stacked ^{1}H-NMR spectra (400 MHz, D_2O) of the crude reaction medium for the degradation of **P4d** at pH 8 after 8 h and 24 h. (B) Photograph of the NMR tube prepared from the crude of reaction after 24 h: an opaque colloid is observed. 51

Figure S 71 – Stacked ^{1}H-NMR spectra (400 MHz, DMSO-d_6) of **P1** (top) and **P1c** (bottom). 51

Figure S 72 – ^{1}H-NMR spectrum (400 MHz, DMSO-d_6) of **P1c**. 52

Figure S 73 – ^{13}C{^{1}H}-NMR spectrum (151 MHz, DMSO-d_6) of **P1c**. 52

Figure S 74 – ^{1}H-NMR spectrum (400 MHz, DMSO-d_6) of **P1cd**. 52

Figure S 75 – ^{13}C{^{1}H}-NMR spectrum (151 MHz, DMSO-d_6) of **P1cd**. 53

Figure S 76 – TGA data for **P1c** and **P1cd**. 53

Figure S 77 – DSC data for **P1c** and **P1cd**. 53

Figure S 78 – Plots used to monitor the kinetics of degradation of **P1cd**: (A Complete plot of the polymer linkage conversion and yield in **2** *vs* time for the degradation of **P1cd**. (B) Plot of the polymer linkage conversion *vs* time until half conversion. A zero-order does not describe well the data. (C) Plot of ln([Polymer]) *vs* time over a broader timescale. A first-order dependence on polymer concentration was observed and a rate constant k_{first} could be extracted from a linear fit. 54

Figure S 79 – Zoomed ^{1}H-NMR spectra (400 MHz, D_2O) of the crude reaction medium for the degradation of **P1cd** at pH 8 after 5 days. (A) Both end groups and free MBA are observed. (B) Both end groups and free ethanol are observed. Integration proves that both small molecules are released at an identical loading. 54

References 55

Materials and Instrumentation

Materials

All experimental manipulations were performed under air unless otherwise specified. All solvents and reagents were obtained from commercial sources and used as received unless stated otherwise. Toluene and dichloromethane (CH_2Cl_2) used in polymerizations were obtained from an SPS system, and further dried with 3 Å molecular sieves, and stored under N_2. Research-grade CO_2 (BOC, CP grade, 99.995%) was dried by passing it through two drying columns (VICI Metronics carbon dioxide purifier) in series at 50 bar pressure before use at lower pressures in the copolymerizations.

- Amberchrom 50WX8 (hydrogen form, 100-200 mesh) was purchased from Sigma Aldrich.
- Benzoic acid (≥99.5%) was purchased from Sigma Aldrich.
- Cholesterol (≥99%) was purchased from Sigma Aldrich. It was dried under vacuum for 24 h.
- Colbalt (II) acetate (99.99%) was purchased from Sigma Aldrich.
- Deuterated dimethylsulfoxide (DMSO-d_6, 99+ atom% D) was purchased from Fisher Scientific.
- Deuterated sulfuric acid (96-98w% in D_2O 99.5 atom% D) was purchased from Sigma Aldrich.
- Deuterium oxide (99.9 atom D) was purchased from Sigma Aldrich.
- 1-Dodecanol (lauryl alcohol, >99%) was purchased from TCI. It was dried over CaH_2 and purified by fractional distillation.
- 4-dimethylaminopyridine (DMAP, ≥98%) was purchased from Sigma Aldrich. It was dried under vacuum for 24 h.
- Dimethylsulfoxide (DMSO-H_6, ≥99.9%) was purchased from Sigma Aldrich.
- Epichlorohydrin (>99%) was purchased from Sigma Aldrich.
- 4-[(2,3-Epoxypropoxy)methyl]-2,2-dimethyl-1,3-dioxolane (**IGG**, 95%) was purchased from Fluorochem. It was dried over CaH_2 and purified by fractional distillation twice.
- Ethyl chloroformate (97%) was purchased from Sigma Aldrich.
- Ethylene diamine (≥99%) was purchased from Sigma Aldrich.
- Hydrochloric acid (36% w/w equiv. solution) was purchased from Alfa Aesar.
- DL-1,2-Isopropylideneglycerol (solketal, 98%) was purchased from Fluorochem. It was dried over CaH_2 and purified by fractional distillation.
- 4-Methyl Benzyl Alcohol (**MBA**, 98%) was purchased from Sigma Aldrich. It was recrystallized from a stirring solution of hot hexane to which was added diethyl ether until complete solubilization. The crystals were formed overnight at 6 °C and were filtered and washed with pentane. The pure product was then dried under vacuum.
- 1-Octadecanol (stearyl alcohol, 99%) was purchased from Sigma Aldrich. It was dried under vacuum for 24 h.
- Oleic acid (≥99%) was purchased from Sigma Aldrich. It was dried under vacuum for 24 h.
- Perfluoro-tert-butanol (tBuF$_3$OH, 97%) was purchased from Fluorochem.
- Potassium acetate (≥99%) was purchased from Sigma Aldrich.
- Potassium hydroxide (≥85%) was purchased from Sigma Aldrich.
- Pyridine (≥99%) was purchased from Sigma Aldrich.
- Sodium bicarbonate ($NaHCO_3$, ≥99%) was purchased from Fluka.
- Sodium carbonate (Na_2CO_3, 99.6%) was purchased from Thermo Scientific.
- Tetrabutylammonium Iodide (TBAI, 98%) was purchased from Sigma Aldrich.
- Triethylamine (for synthesis) was purchased from Sigma Aldrich.

The catalyst [Co(III)/K(I)] was synthesized from the dialdehyde pro-ligand (Manchester Organics), via a literature procedure.[1]

Instrumentation

Nuclear Magnetic Resonance (NMR) Spectroscopy. ^{1}H and ^{13}C{^{1}H} NMR spectroscopy were performed at 25 °C using several instruments: Bruker Avance III HD nanobay 400 MHz, Bruker Avance III HD 500 MHz, Bruker NEO 600, with broadband helium cryoprobe equipped with a 14.1T magnet.

Size-Exclusion Chromatography (SEC, THF). Polymer samples dissolved in HPLC grade THF were syringe filtered through 25 µm PTFE filters. Next, they were injected into Shimadzu LC-20AD SEC instrument, with two PSS SDV 5 µm linear M columns, heated to 30 °C. HPLC grade THF was used as the eluent, at a flow rate 1.0 mL min^{-1}. RI and UV detectors were calibrated, using a series of narrow molecular weight polystyrene standards. Shimadzu SEC post run program was used to analyse the data.

Size-Exclusion Chromatography (SEC, DMF/LiBr). Polymer sample solutions were analysed using a 1260 Infinity II GPC MDS (refractive index detection only), equipped with a PSS GRAM guard column (8 × 50 mm, 10 µm) and two PSS GRAM linear columns (8 × 300 mm, 10 µm, 500–1 000 000 Da). The eluent was HPLC grade DMF containing 0.075% (w/w) LiBr and was run at a flow rate of 1 mL min^{-1} at 40 °C. Molecular weight calibration was performed using near-monodisperse poly(methyl methacrylate) standards (EasiVial, Agilent).

MALDI-TOF Mass Spectroscopy. Analyses were carried out on a Bruker Autoflex Speed MALDI-TOF. Spectra were acquired in positive-reflectron mode. Spotting method A, for polycarbonates: sample solutions of polymer (10 mg mL^{-1} in THF), dithranol (10 mg mL^{-1} in THF), and KTFA (10 mg mL^{-1} in MeOH) were pre-mixed in 1:4:1 ratio and subsequently spotted onto a metal plate and allowed to fully evaporate before analysis. Spotting method B, for hydroxyl-containing polycarbonates: sample solutions of polymer (10 mg mL^{-1} in H_2O), 2,5-Dihydroxybenzoic acid (10 mg mL^{-1} in MeOH), and KTFA (10 mg mL^{-1} in MeOH) were pre-mixed in 1:4:1:1 ratio and subsequently spotted onto a metal plate and allowed to fully evaporate before analysis.

High Resolution Mass Spectroscopy (HRMS). Solutions of the pure compounds of interest (**1** in acetonitrile and **2** in water) were prepared to a concentration of 1 mg/L and filtrated over nylon filters (0.22 µm). Flow injection analysis was performed on an ACQUITY I-Class PLUS UPLC System (Waters, Milford, MA, USA) coupled to an ACQUITY RDa mass spectrometer (Waters, Milford, MA, USA) equipped with an ESI probe, in positive ion mode. The flow rate was set to 0.300 mL/min using a 50% methanol$_{(aq)}$ + 0.1% formic acid eluent. Scan parameters were set as follows: analyser mode, full scan; scan range, 50 2000 *m/z*; scan rate, 2 Hz; cone voltage, 30 V; capillary voltage, 1.5 kV; desolvation temperature, 550 °C; and intelligent data capture, on.

Thermogravimetric Analysis (TGA). Polymer samples were analysed on a TGA/DSC 1 (Mettler-Toledo Ltd), or a TGA5500 (TA Instruments). Polymer samples were heated from 40 to 800 °C at a rate of 10 °C min^{-1}, under N_2 flow (50 mL min^{-1}).

Differential Scanning Calorimetry (DSC). Polymer samples were analysed on a DSC25 (TA Instruments), under a N_2 flow (50 mL min^{-1}). Samples were heated (10 °C min^{-1}) and equilibrated to 100 °C, then cooled to −80 °C (10 °C min^{-1}) before heating a second time to 100 °C, at a rate of 10 °C

min^{-1}. Glass transition temperatures (T_g) were determined from the midpoint of the transition in the second heating curve.

In-situ IR Spectroscopy. The copolymerizations were monitored using a ReactIR 15 instrument, including a liquid nitrogen MCT detector and a DiComp ATR-IR probe, bottom-mounted onto a 100 mL Parr Reactor. An IR spectrum was generated once every minute (from 256 scans), recording within a wavenumber range of 4000-650 cm^{-1}

Rheology. Oscillatory shear measurements were performed on an ARES-G2 rheometer (TA Instruments) between two stainless steel parallel plates (25 mm for **P1** and 8 mm for **P1d**). Measurements were performed in the viscoelastic linear region after performing amplitude sweeps at lower and upper temperatures. *For **P1**,* temperature ramps were performed at a rate of 2 °C/min, frequency of 2 Hz and constant strain of 5 %. Consecutive frequency sweeps (strain 5%) were performed from lower to upper usage temperatures and an equilibration time of 5 minutes between measures. *For **P1d**.* Temperature ramps were performed at a rate of 2 °C/min, frequency of 2 Hz and constant strain of 0.5 %. Consecutive frequency sweeps (strain 0.5%) were performed from lower to upper usage temperatures and an equilibration time of 5 minutes between measures.

ATR-IR Spectroscopy. Infrared spectra were recorded on a Bruker Tensor 27 Fourier Transform spectrometer using diamond ATR.

DLS. Aqueous polymer samples (90:10 H_2O/THF) were analysed using a Zetasizer Pro Blue system (Malvern Instruments). The scattering angle was fixed at 173°. Data processing was carried out using cumulant analysis of the experimental correlation function and the Stokes−Einstein equation was used to calculate the hydrodynamic radii. All solutions were analysed using polystyrene cuvettes.

Tensiometer. Surface tension was carried out using a Kibron Delta 8 Tensiometer which is based on the DeNouy method with a platinum rod probe. Serial dilutions of 0.5 from a concentrated stock solution were performed using a multi-channel micropipette into a 12 x 8 welled plate. Each well was filled with a total sample volume of 100 µL. A total of 4 replicates were measured for each concentration. A purified distilled water control was present on each plate. For the amphiphile polymer measurement, the tensiometry data was fitted to a 5-parameter logistic function using the following equation, where γ is the surface tension value at a concentration x, A_{min} and A_{max} the lower and upper plateau values, h the slope, s the asymmetry parameter defining the "broadness" of the transition, and x_0 the scale parameter.

$$\gamma = A_{min} + \frac{A_{max} - A_{min}}{(1 + \left(\frac{x_0}{x}\right)^h)^s}$$

CryoTEM. Lacey 400 mesh with 2 nm carbon (Agar) Grids were used without glow discharge. For each sample, 3.5 µL material was applied before plunge freezing with a GP2 (Leica) operating at room temperature and 95 % reported humidity and manual plunging. Clipped grids were screened on a Glacios (thermofisher) operating at 200 kV. Data were collected using EPU software with a magnification of 92 kX (pixel size 1.54 A/piX) and dose of 40 e/A2 in linear mode.

Experimental procedures

Synthesis and purification of IGG

IGG was synthesized by modification of a reported procedure.[2] In a three-necked round bottom flask, solketal (282 g, 1.14 mol, 1 equiv.) and epichlorohydrin (500 mL, 590 g, 6.4 mol, 5.6 equiv.) were dissolved into THF (1050 mL) under N_2. The flask was placed in an ice bath, adding potassium hydroxide pellets (169 g, 3 mol, 2.65 equiv.) previously crushed in a mortar. The reaction was kept at 0 °C for 3 hours, then left at room temperature overnight, and finally heated it to 50 °C for 2 hours. After cooling to ambient temperature, insoluble materials were filtered and the solution concentrated via rotary evaporation. The residue was diluted with CH_2Cl_2 (1000 mL) and washed with a 10 w/v% $NaHCO_3$ solution (3 x 300 mL) followed by brine (2 x 300 mL). The organic phase was recovered, dried over $MgSO_4$, filtered, and concentrated in vacuo. The product was purified by a first distillation under reduced pressure to yield **IGG** as a colorless oil (282 g, isolated yield 70%). It was then more thoroughly purified before ROCOP. It was dried over CaH_2 and purified by fractional distillation twice.

Representative Polymerization of **IGG** and CO_2 into **P1** using MBA as initiator

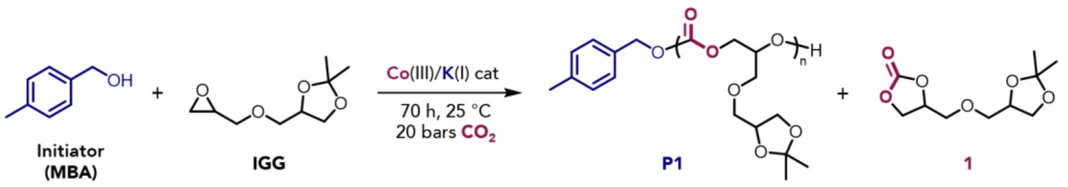

Synthesis

In a glovebox, an airtight vial was charged with [Co(III)/K(I)] catalyst (29 mg, 0.046 mmol, 1 equiv.), methyl benzyl alcohol (MBA) (224 mg, 1.83 mmol, 40 equiv.), **IGG** (8 mL, 8.63 g, 45.87 mmol, 1000 equiv.) and dry toluene (2 mL). The mixture was stirred until the solution was homogeneous. Then, the reaction mixture was transferred into a 25 mL Parr reactor and removed from the glovebox. The reactor was pressurized at 20 bar of CO_2 and was stirred, at 25 °C, for 70 h. The reactor was then depressurized and the crude reaction mixture was quenched by addition of benzoic acid (10 equiv.). Crude samples were analysed by ^{1}H-NMR spectroscopy in $CDCl_3$ to determine the reaction conversion and selectivity. The polymer was then precipitated once in pentane and recovered by centrifugation. It was dissolved in a solution of CH_2Cl_2 / Triethylamine (99/1) (100 mL) and passed through a short silica column using the same solution as eluent. The solvent was then removed under reduced pressure. The concentrated polymer solution was diluted with CH_2Cl_2 (10 mL), precipitated in pentane and recovered by centrifugation. This step was repeated two additional times. The viscous material was dried for 24 h under vacuum at room temperature to yield pure **P1** as a viscous transparent liquid (6.5 g, isolated yield 61 %).

It must be noted that in the presence of persistent cyclic carbonate byproduct **1**, the polymer solution could be precipitated in a mixture of pentane and ethanol (ratio of 75/25). Although more efficient to remove this impurity, a reduction in isolated yield was observed.

Monitoring

The same procedure was applied for the in-situ IR spectroscopic monitoring of the reaction. However, the reaction mixture was prepared in a Schlenk flask and was removed from the box. Under a flow of CO_2, the solution was injected in a mounted 100 mL Parr reactor, with a fixed bottom-mounted IR probe. The reactor was then pressurized. The evolution of the reaction in polycarbonate **P1** and cyclic carbonate **1** was observed by following characteristic bands at 1750 cm^{-1} (for **P1**) and 1815 cm^{-1} (for **1**).

Calculations

From the crude of reaction, the **IGG** conversion was calculated using the resonances from the polymer (5.02 ppm, 1H), the cyclic carbonate **1** (4.81 ppm, 1H) and **IGG** (2.79 ppm, 1H) highlighted in Figure S3. The following equation was used:

$$Conv.(\%) = \left(\frac{I(5.02) + I(4.81)}{I(5.02) + I(4.81) + I(2.79)}\right) \times 100$$

Where I is the integral value of the selected resonance.

The selectivity was determined using the same resonances of the polymer and product **1**, using the following equation:

$$Select.(\%) = \left(\frac{I(5.02)}{I(5.02) + I(4.81)}\right) \times 100$$

Where I is the integral value of the selected resonance.

From the purified polymer, the DP was calculated from the resonance of the polymer and a characteristic resonance from the initiator. For MBA, the characteristic aromatic protons (7.16 ppm, 2H) or the methyl group (2.34 ppm, 3H) could be used. The following equation was used:

$$DP = \frac{I(5.02)}{\frac{I(7.16)}{2}}$$

Where I is the integral value of the selected resonance.

Representative acetal deprotection strategies

Representative deprotection – Protocol A (using an acidic resin)

The protected polymer **P1** (1 g) was dissolved in a solvent MeOH/THF (1:1) mixture (8 mL), at room temperature, until a homogeneous solution was obtained. Water (1 mL) and 10 w/w % acidic ion exchange resin (Dowex® 50WX8) (100 mg) were added to the solution. The reaction was heated to 50 °C, without stirring. The ion exchange resin was removed, by centrifugation, and the solution was concentrated, in vacuum, and dried (in vacuo) to yield pure deprotected polymer **P1d** (850 mg, isolated yield 95 %).

Representative deprotection – Protocol B (using hydrochloric acid)

The protected polymer **P1** (1 g) was dissolved in acetonitrile (5 mL), at room temperature, until a homogeneous solution was obtained. Water (2.5 mL) was added, followed by a 1M HCl solution (2.5 mL of solution, 0.6 equiv. HCl *vs* acetal group). After 1 h, the polymer was precipitated in cold acetonitrile. The mixture was vortexed and centrifuged (2 x 5 min). The viscous liquid was dried, under vacuum at room temperature, yielding a foaming sticky solid **P1d** (830 mg, isolated yield 93 %).

Synthesis of **P2-P6**

The synthetic protocol follows that of **P1**. The different initiators were used, always following the ratio [cat]:[initiator]:[**IGG**] = 1:40:1000. When the initiator was insoluble in toluene, CH_2Cl_2 was used instead.

Synthesis of **1** and **2**

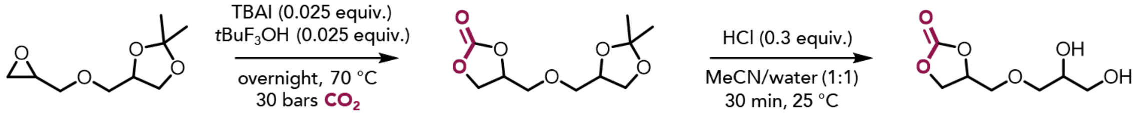

IGG (10 g, 53.2 mmol, 1 equiv.), TBAI (491 mg, 1.33 mmol, 0.025 equiv.) and perfluoro-tert-butanol (314 mg, 1.33 mmol, 0.025 equiv.) were stirred in a vial. The mixture was transferred in a Stainless-steel reactor and the vessel was pressurized to a constant pressure of 30 bar of CO_2 at 70 °C overnight. The reactor was cooled, depressurized, and the liquid was diluted in dichloromethane (50 mL). The solution was passed through a short silica column with diethyl ether / triethylamine (98/2) as eluent. Additional eluent (100 mL) was passed through the column and the pure product **1** was obtained as a transparent viscous oil (7.34 g, isolated yield 60 %) after removal of solvent under reduced pressure. **^{1}H NMR** (400 MHz, DMSO-d$_6$) δ 4.93 (m, 1H), 4.53 (t, J = 8.4 Hz, 1H), 4.26 (dd, J = 8.0, 5.96 Hz, 1H), 4.18 (quintet of d, J = 5.9, 1.26 Hz, 1H), 3.98 (dd, J = 8.2, 6.6 Hz, 1H), 3.76-3.58 (m, 3H), 3.54-3.49 (m, 2H), 1.29 (d, J = 18 Hz, 6H); **^{13}C NMR** (101 MHz, DMSO-d$_6$) δ 155.4, 109.0 (d), 76.0 (d), 74.7 (d), 72.2 (d), 70.8 (d), 66.4 (d), 66.1 (d), 27.0, 25.9 (d). **HRMS (ESI)**: Calculated for $C_{10}H_{16}O_6$ $[M+H]^+$: 233.1020. Found: 233.1019.

1 (400 mg, 1.72 mmol, 1 equiv.) was dissolved in acetonitrile (1 mL). Water (0.5 mL) and a 1 M aqueous solution of HCl (0.5 mL, 0.5 mmol, 0.3 equiv.) were subsequently added to the solution, and the mixture was stirred, at 25 °C, for 30 min. Acetonitrile (30 mL) was added to the mixture in a round-bottom flask and the solvent was reduced under pressure. The addition of acetonitrile (30 mL) and solvent removal were repeated twice. The product was dried, under vacuum, to yield the pure product **2** as a transparent viscous oil (284 mg, isolated yield 86 %). **^{1}H NMR** (400 MHz, DMSO-d_6) δ 4.92 (m, 1H), 4.69 (dd, J = 5.06, 2 Hz, 1H), 4.52 (m, 2H), 4.29 (m, 1H), 3.72-3.66 (m, 1H), 3.64-3.54 (m, 2H), 3.52-3.44 (m, 1H), 3.42-3.26 (m, 3H); **^{13}C NMR** (101 MHz, DMSO-d_6) δ 155.4, 76.1 (d), 73.5 (d), 71.0, 70.8, 70.7 (d), 66.5, 63.4 (d). **HRMS (ESI)**: Calculated for $C_7H_{12}O_6$ $[M+H]^+$: 193.0707. Found: 193.0708.

Representative methodology for the monitoring of degradation

The polymer **P2d** (375 mg) was dissolved in D_2O (13.125 mL) and the internal standard DMSO-*H_6* (25 µL) was added. The medium was aliquoted as t_0. A 0.8 M solution of the phosphate buffer in D_2O (1.875 mL, pH = 8) was added to the mixture to start the reaction, reaching a final buffer concentration of 0.1 M and a final polymer concentration of 25 mg mL^{-1}. The crude reaction medium was aliquoted (500 µL) for ^{1}H NMR spectroscopy analysis and quenched in D_2O (150 µL) acidified with D_2SO_4 (2 µL).

For **P2d** only, aliquots (500 µL) were analyzed by SEC (in DMF/LiBr) after dilution in the SEC eluent (1.25 mL) acidified with D_2SO_4 (2 µL). After 15 min, some inorganic salt crashed out and the solution was filtered over nylon filters (0.22 µm).

Monitoring the degradation kinetics by ^{1}H NMR spectroscopy

The reaction was monitored by integration of characteristic signals from both the polymer and product **2**, relative to the internal standard. At t_0, the integral of the polymer (δ_{1H} = 5.14 ppm) relative to the one of DMSO-*H_6* (internal standard, δ_{1H} = 2.71 ppm, normalized to an integral of 1) was defined as the starting value of content in linkage before degradation, I(P_{max}).

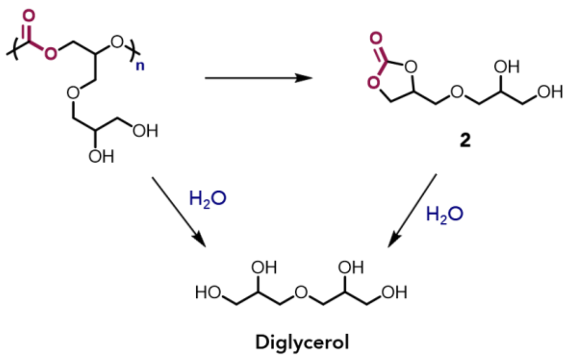

As degradation took place, the signal of the polymer (δ_{1H} = 5.14 ppm) overlapped with a signal from **2** (δ_{1H} = 5.07 ppm). The two signals were therefore used for integration (**P2d** + **2**, I(5.14+5.07)) and compared to an isolated peak of **2** (δ_{1H} = 4.66 ppm) – this signal corresponded to a well-resolved triplet and was therefore fitted to provide a more accurate integration (using the MestReNova software, see Figure S63). The amount of remaining polymer during degradation was estimated by subtracting the

overlapping signal of both **P2d** and **2** to the isolated signal of **2** (δ_{1H} = 4.66 ppm). The polymer conversion, yield in **2**, and yield in diglycerol could be calculated using the following equations:

$$Polym.conv.(\%) = (1 - \frac{I(5.14 + 5.07) - I(4.66)}{I(P_{max})}) \times 100$$

$$Yield\ in\ \mathbf{2}\ (\%) = \left(\frac{I(4.66)}{I(P_{max})}\right) \times 100$$

$$Yield\ in\ \boldsymbol{diglycerol}\ (\%) = \left(1 - \frac{I(5.14 + 5.07)}{I(P_{max})}\right) \times 100$$

Where *I* is the integral value of the selected resonance.

Synthesis of P1c

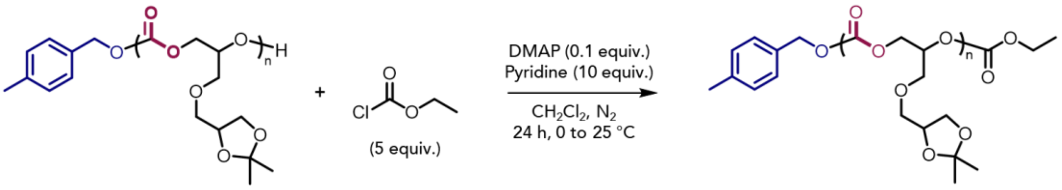

The pure polymer **P1** (1.76 g, 0.41 mmol of hydroxyl end group, 1 equiv.) was added to an air-tight vial, and was dried under vacuum for 2 h, before the start of reaction. In a glovebox, dry CH_2Cl_2 (14 mL) was added to the vial to dissolve the polymer. Once a solution was obtained, the vial was charged with ethyl chloroformate (195 µL, 2.05 mmol, 5 equiv.) and removed from the glovebox. The mixture was cooled to 0 °C, in an ice bath and pyridine (331 µL, 4.1 mmol, 10 equiv.) and DMAP (5 mg, 0.041 mmol, 0.1 equiv.) were added. The mixture was stirred for 1 h at 0 °C, and was then allowed to reach room temperature. After 24 h, the reaction was quenched with excess ethanol (400 µL, 6.86 mmol, 16.7 equiv.) and stirred, open to air, for 30 min. The polymer was then precipitated in pentane (50 mL). The viscous liquid was dissolved in CH_2Cl_2 (8 mL) and extracted with water (35 mL) three times. When appearing, emulsions were easily broken by centrifugation. The organic phase was then dissolved in more CH_2Cl_2 (40 mL) and dried with $MgSO_4$, followed by filtration and removal of the solvent under reduced pressure. The viscous material was dried under vacuum at room temperature to yield pure **P1c** as a viscous transparent liquid (1.48 g, isolated yield 84 %).

Methodology for the monitoring of degradation of 2

The isolated product **2** (17.5 mg) was dissolved in D_2O (612 µL) and the internal standard DMSO-H_6 (2 µL) was added. The solution was transferred in an NMR tube and was analyzed as t_0. A 0.8 M solution of the phosphate buffer in D_2O (1.875 mL, pH = 8) was added to the mixture to start the reaction, reaching a final buffer concentration of 0.1 M and a final concentration in **2** of 25 mg mL^{-1}. The NMR tube was shaken for several minutes, and was stored at room temperature for analysis over time.

Monitoring by ^{1}H NMR

The reaction was monitored by integration of characteristic signal from **2** (δ_{1H} = 5.07 ppm) relative to the internal standard. At t_0, the integral of **2** (δ_{1H} = 5.07 ppm) relative to the one of DMSO-H_6 (internal standard, δ_{1H} = 2.71 ppm, normalized to an integral of 1) was defined as the starting concentration in **2** $I(\mathbf{2}_{max})$. The conversion in **2** was calculated using the following equation:

$$Conv.in\ \mathbf{2}\ (\%) = (\frac{I(5.07)}{I(\mathbf{2}_{max})}) \times 100$$

Where *I* is the integral value of the selected resonance.

ROCOP mechanism

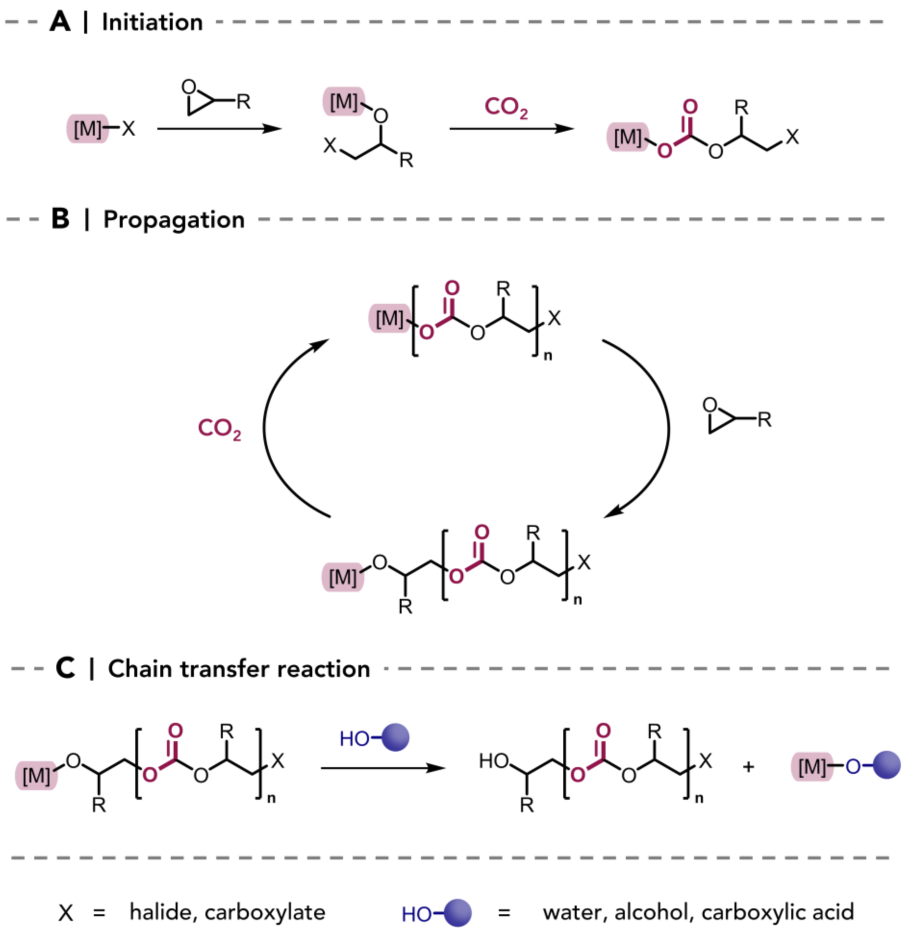

Scheme S 1 – Key steps of the ROCOP of CO_2 and epoxides.

Characterization of IGG

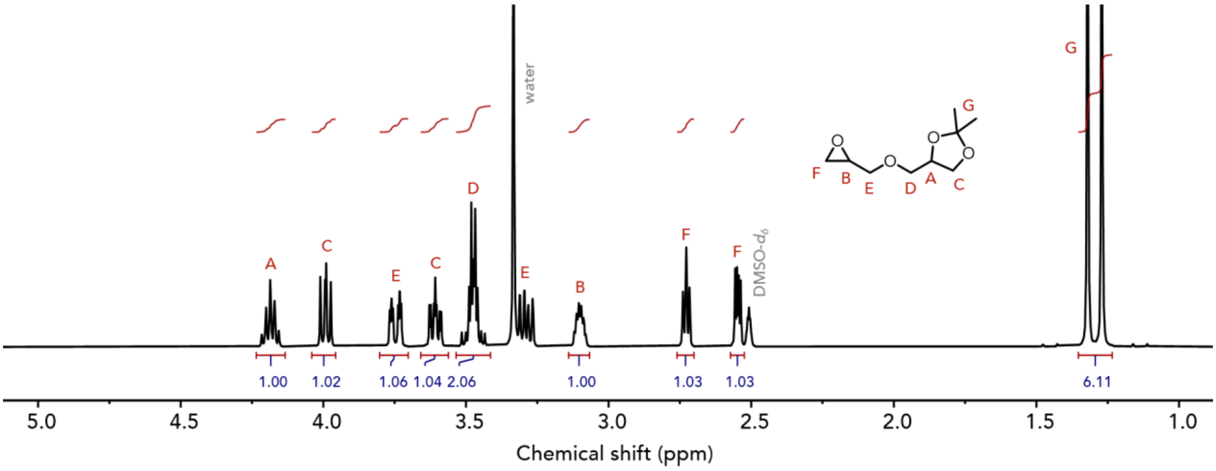

Figure S 1 – ^{1}H-NMR spectrum (400 MHz, DMSO-d_6) of **IGG**.

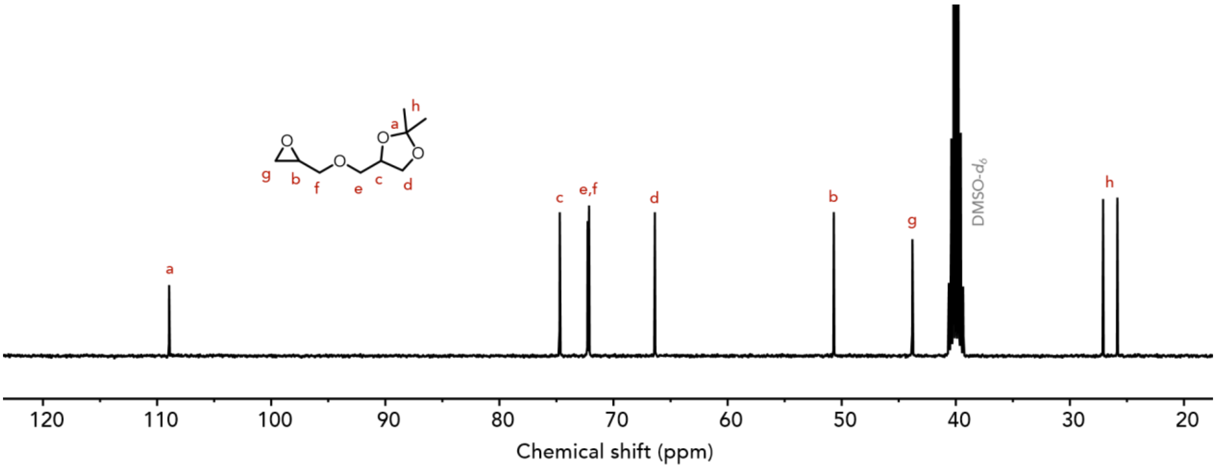

Figure S 2 – ^{13}C{^{1}H}-NMR spectrum (101 MHz, DMSO-d_6) of **IGG**.

Synthesis and characterization of P1

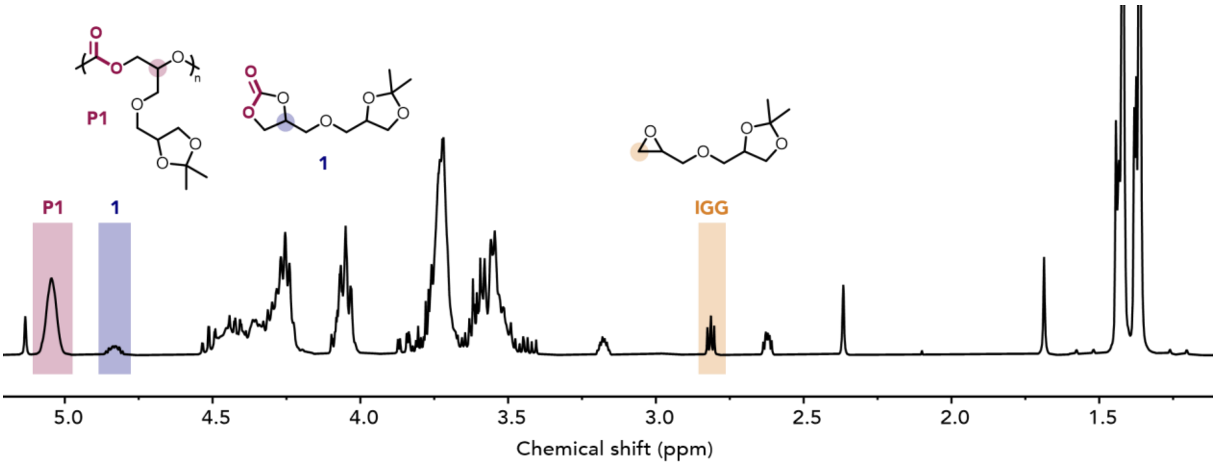

Figure S 3 – Typical ^{1}H-NMR spectrum (400 MHz, $CDCl_3$) of a crude reaction medium.

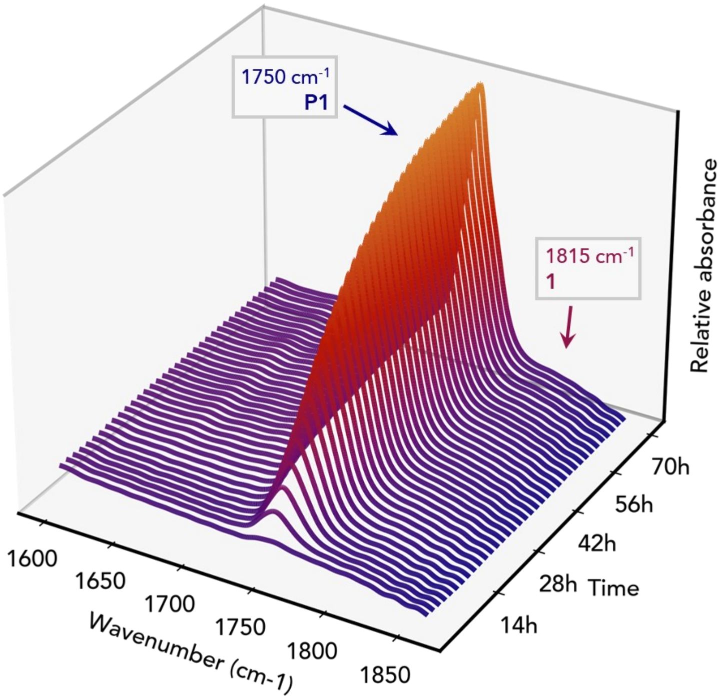

Figure S 4 – In-situ IR spectra monitoring used for the ROCOP of CO_2 and **IGG** at 25 °C (Table 1, entry 4).

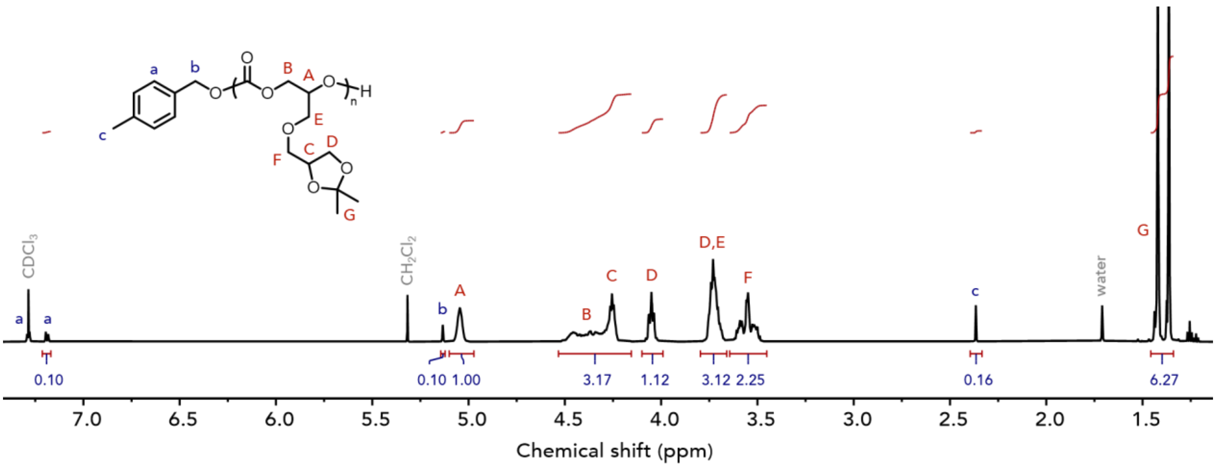

Figure S 5 – ^{1}H-NMR spectrum (600 MHz, $CDCl_3$) of **P1**.

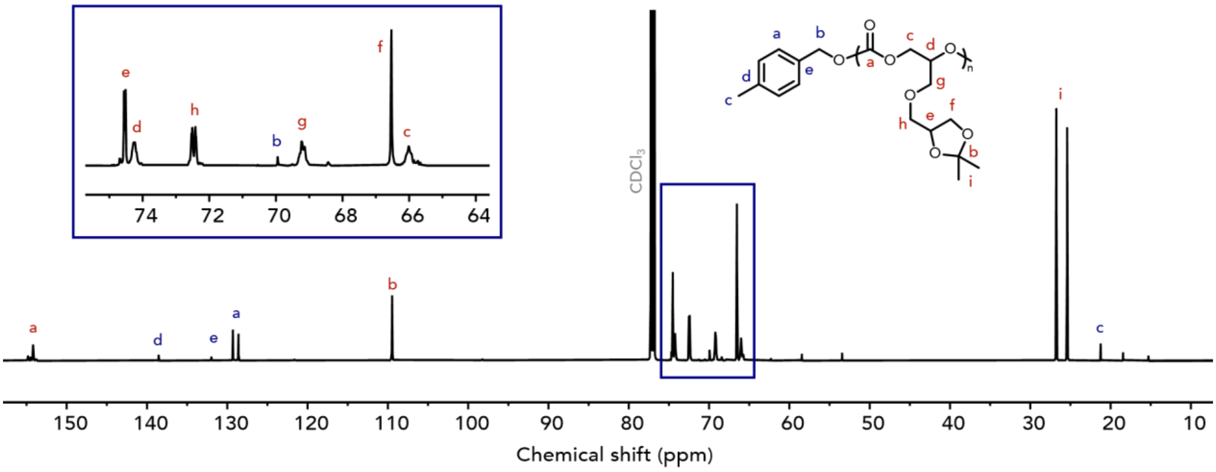

Figure S 6 – $^{13}C\{^{1}H\}$-NMR spectrum (151 MHz, $CDCl_3$) of **P1**.

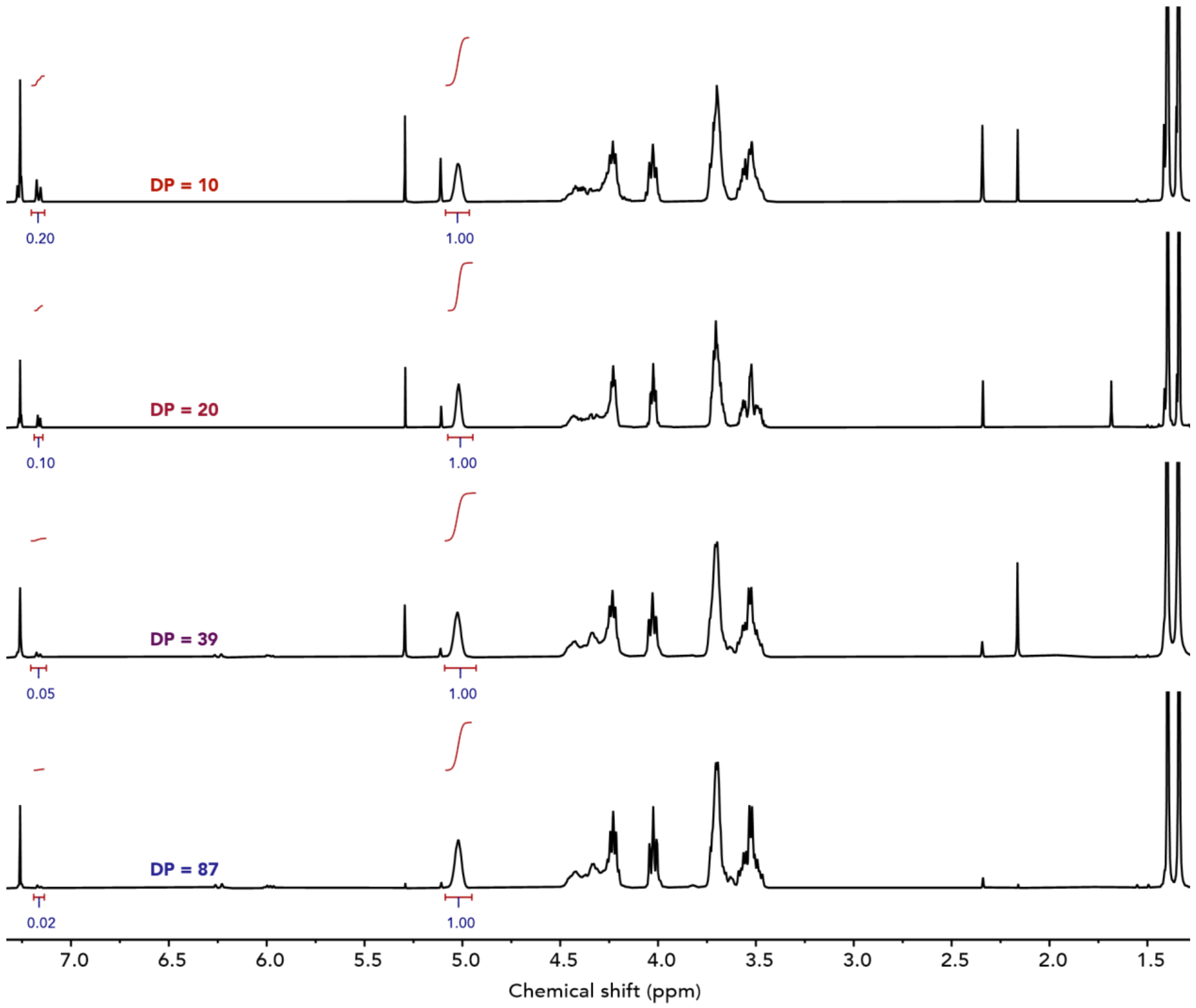

Figure S 7 – Stacked ^{1}H-NMR spectra (400 MHz, $CDCl_3$) of derivatives of **P1** with varied degree of polymerization (DP).

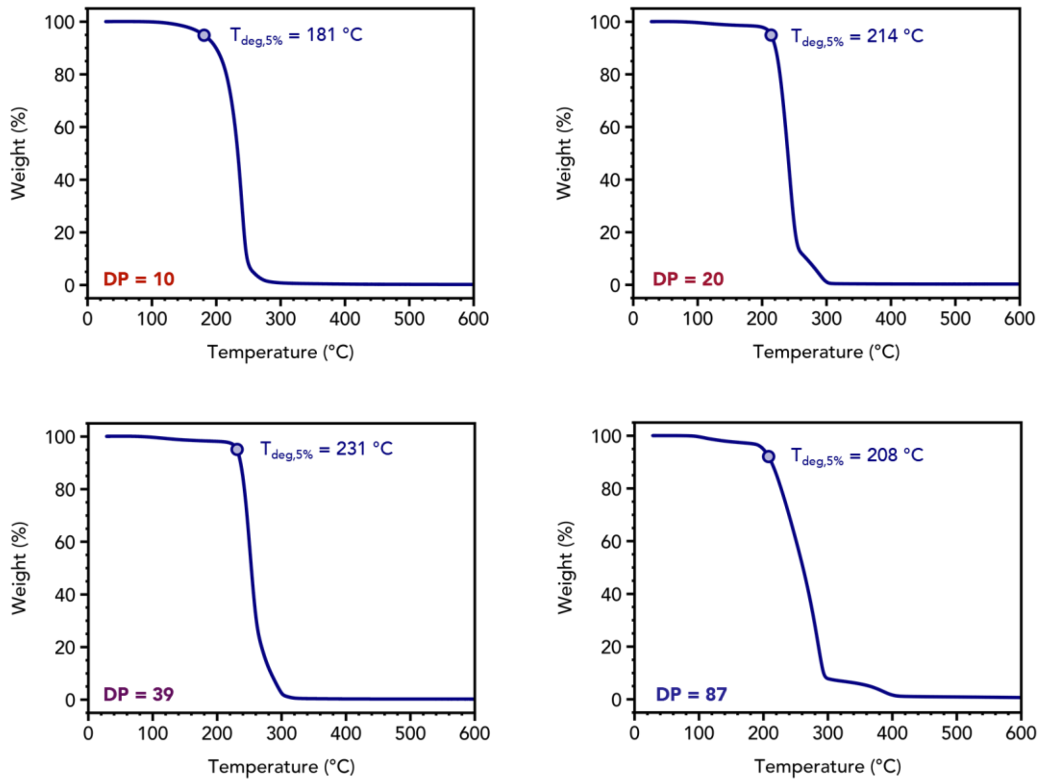

Figure S 8 – TGA data of **P1** samples with varied degree of polymerization (DP).

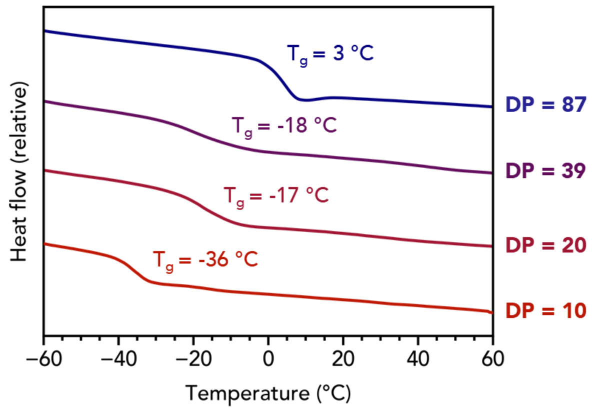

Figure S 9 – Stacked DSC data for **P1** samples with varied degree of polymerization (DP).

<u>**Deprotection of acetal in P1 and characterization of P1d**</u>

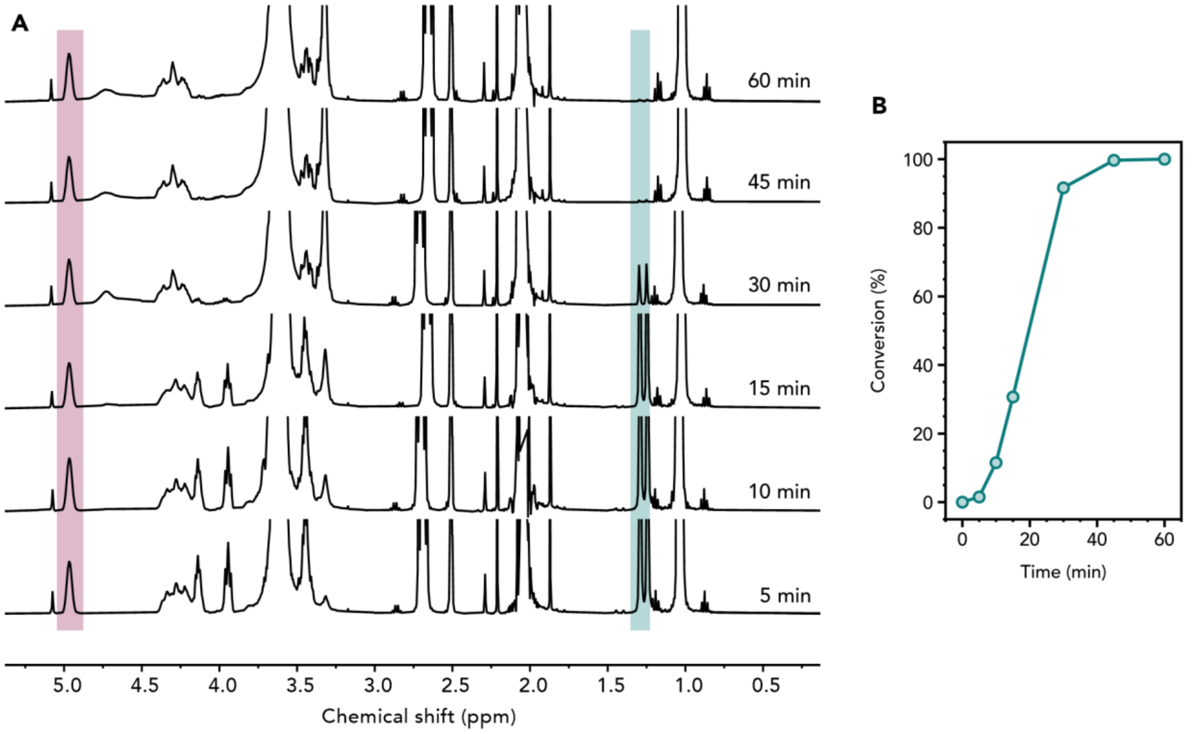

Figure S 10 – (A) Stacked ^{1}H-NMR spectra (400 MHz, DMSO-d_6) of the crude reaction medium for the deprotection of **P1** into **P1d**, following protocol **B**. Over time (from bottom to top), the typical resonance of the polymer at 4.97 ppm remains intact, while the resonances attributed to the acetal groups at 1.24 and 1.29 ppm disappeared. (B) Plot of acetal conversion *vs* time for the deprotection of **P1**.

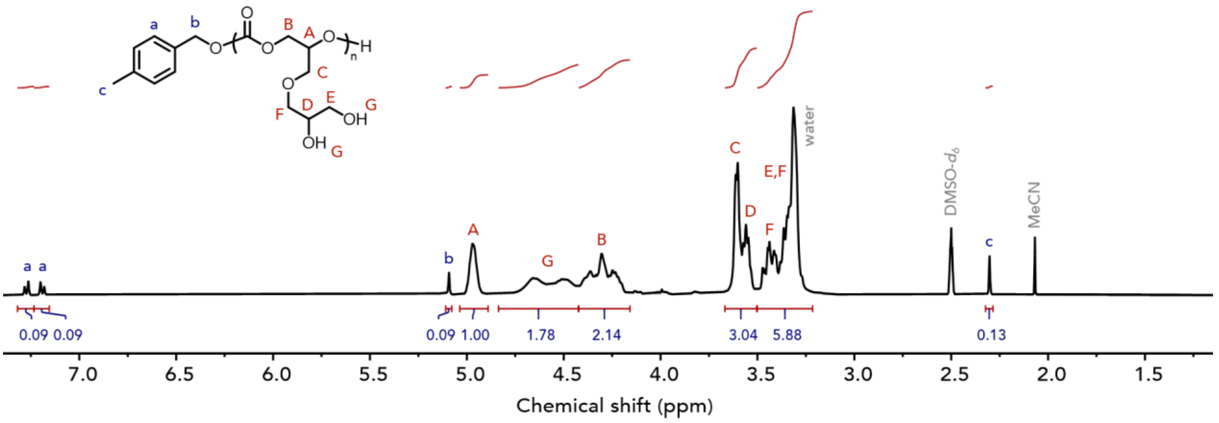

Figure S 11 – ^{1}H-NMR spectrum (400 MHz, DMSO-d_6) of **P1d**.

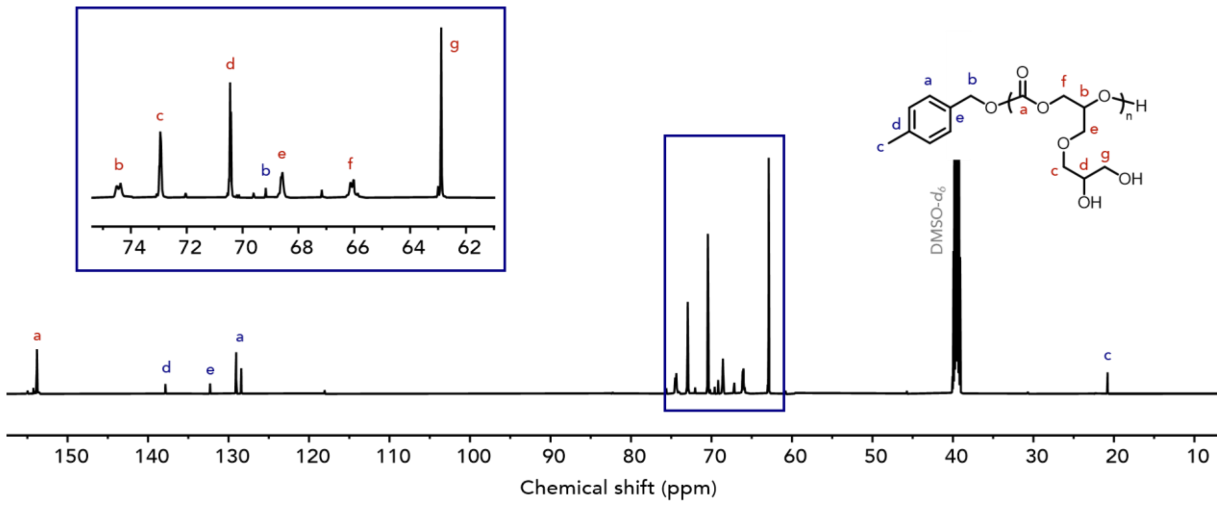

Figure S 12 – $^{13}C\{^{1}H\}$-NMR spectrum (151 MHz, DMSO-d_6) of **P1d**.

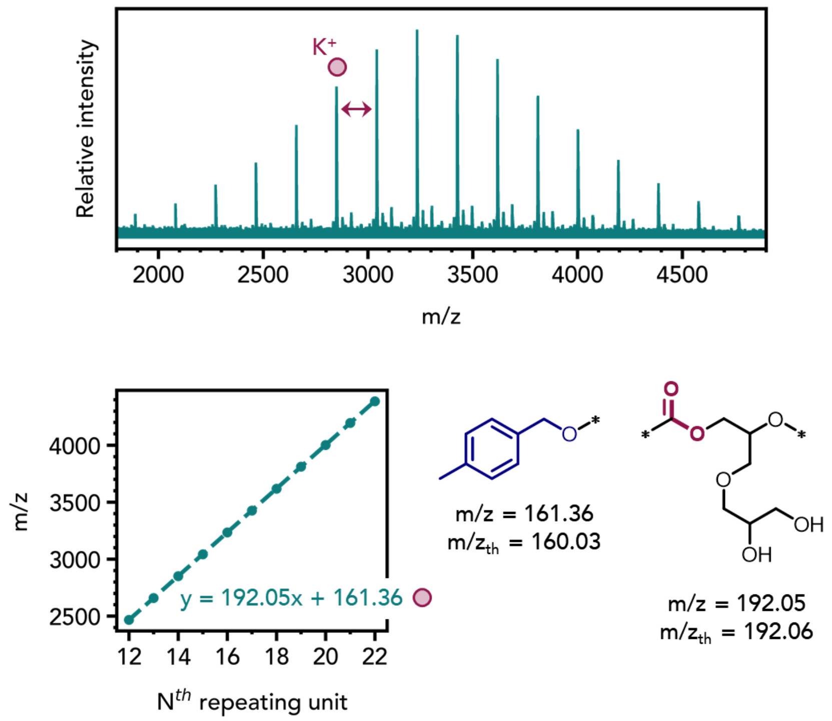

Figure S 13 – (A) MALDI-TOF spectrum of **P1d** (DP 20). (B) Plot of *m/z* vs Nth repeat unit. The theoretical molecular weights of the repeating unit and the end group match the experimental values.

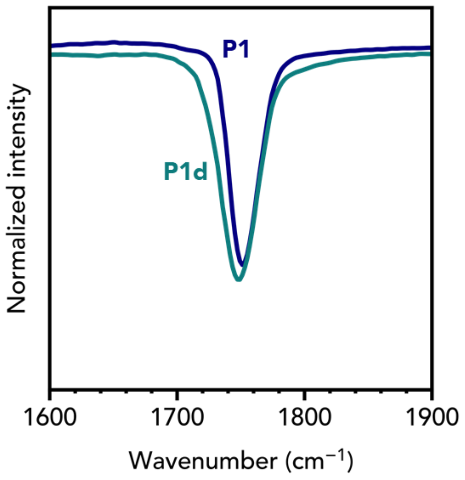

Figure S 14 – Zoomed ATR-IR spectra showing the C=O band for **P1** (blue) and **P1d** (green) (DP = 20).

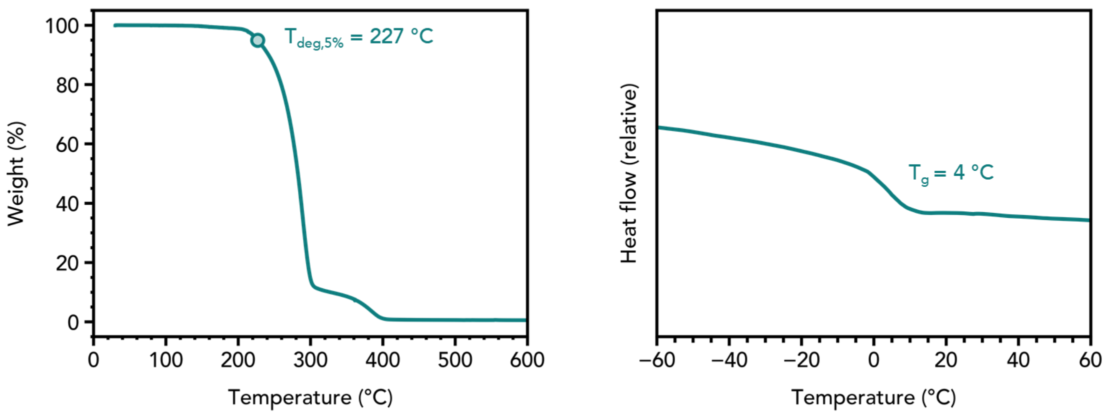

Figure S 15 – TGA (left) and DSC (right) data for **P1d** (DP = 20).

Rheological analyses of P1 and P1d

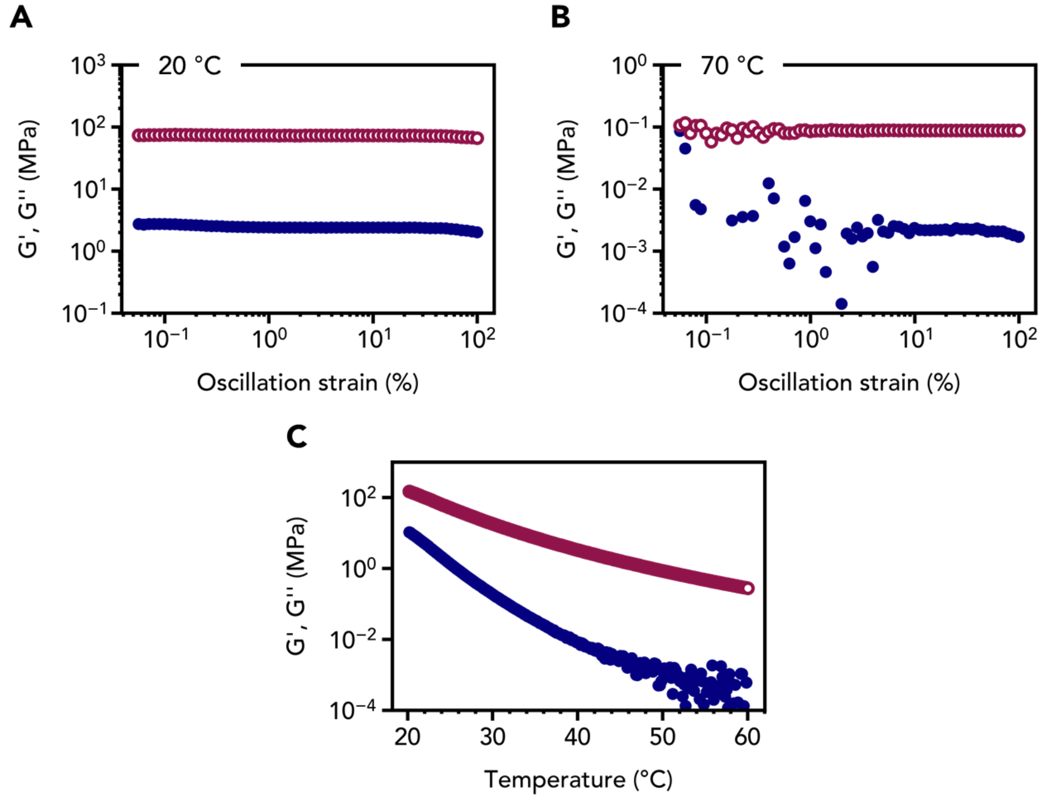

Figure S 16 – Rheological analyses for samples of **P1**: (A,B) Amplitude sweeps (frequency = 2 Hz) at 20 and 70 °C. (C) Temperature sweep (frequency = 2 Hz; strain = 5 %).

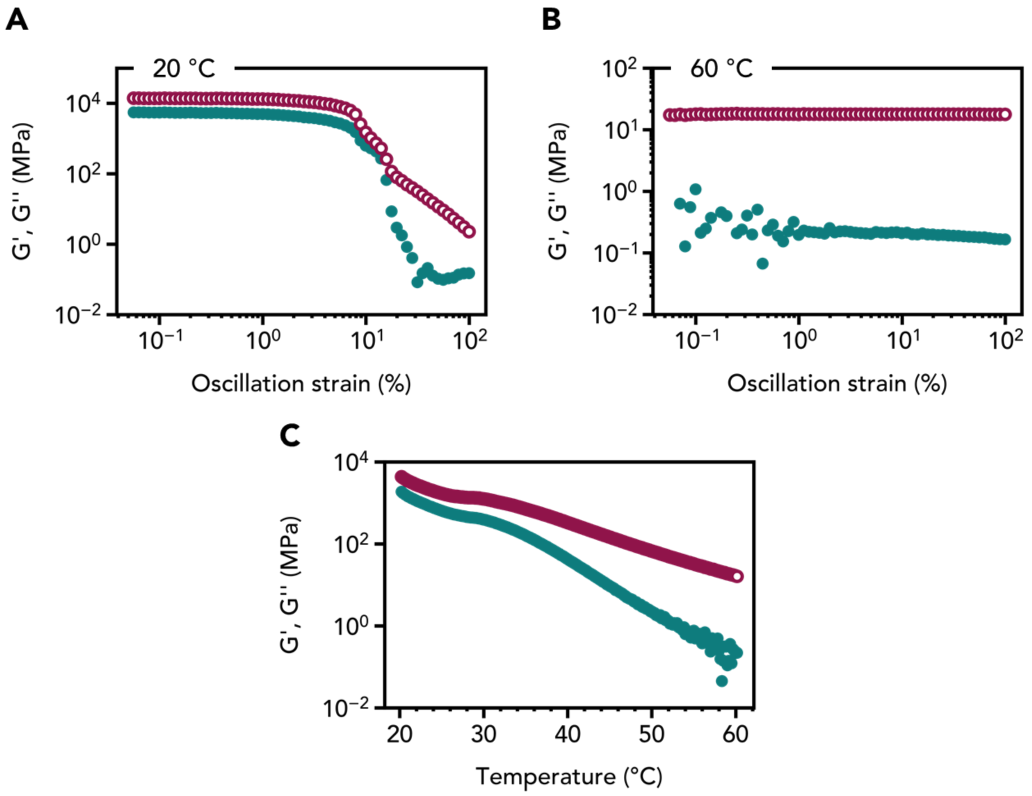

Figure S 17 – Rheological analyses for samples of **P1d**: (A,B) Amplitude sweeps (frequency = 2 Hz) at 20 and 60 °C. (C) Temperature sweep (frequency = 2 Hz; strain = 0.5 %).

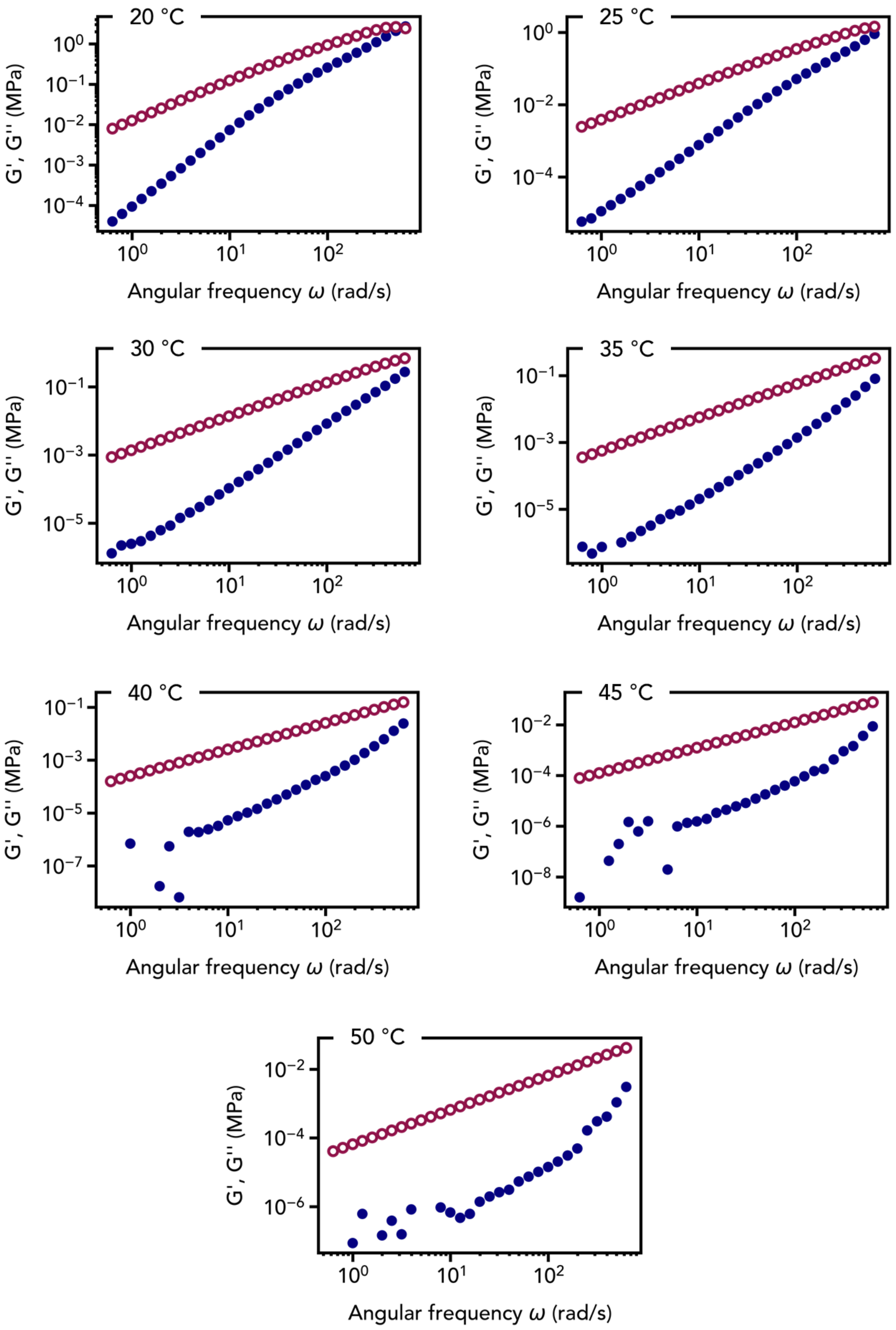

Figure S 18 – Rheological analyses for **P1**: Frequency sweeps (strain = 5 %) from 20 to 50 °C.

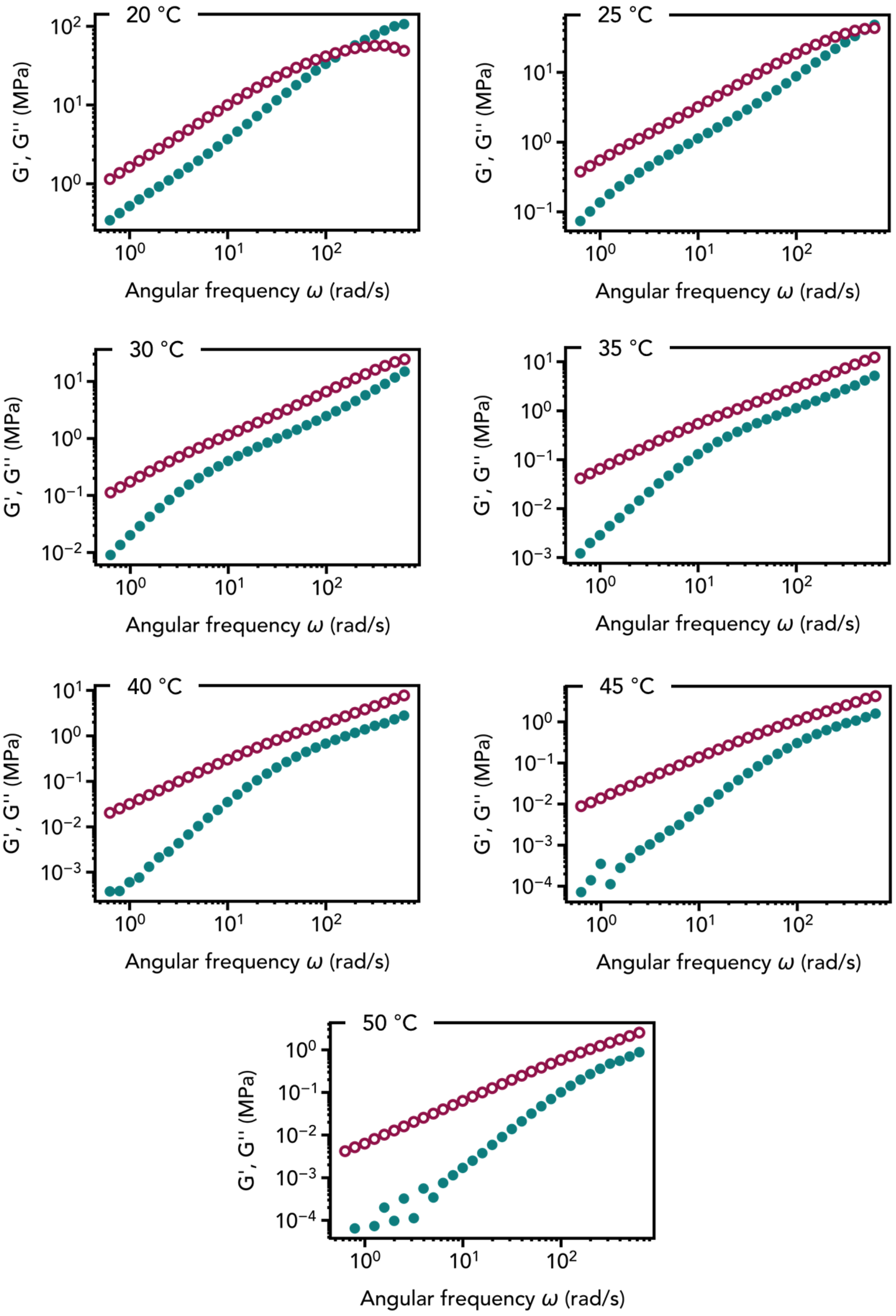

Figure S 19 – Rheological analyses for **P1d**: Frequency sweeps (strain = 0.5 %) from 20 to 50 °C.

Characterization of P2-6 and P2d-6d

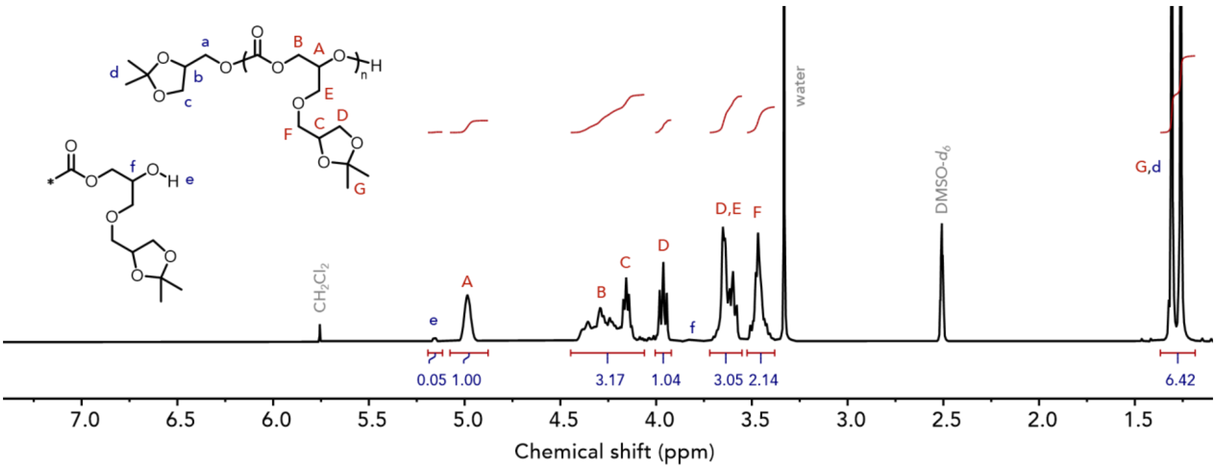

Figure S 20 – ^{1}H-NMR spectrum (400 MHz, DMSO-d_6) of **P2**.

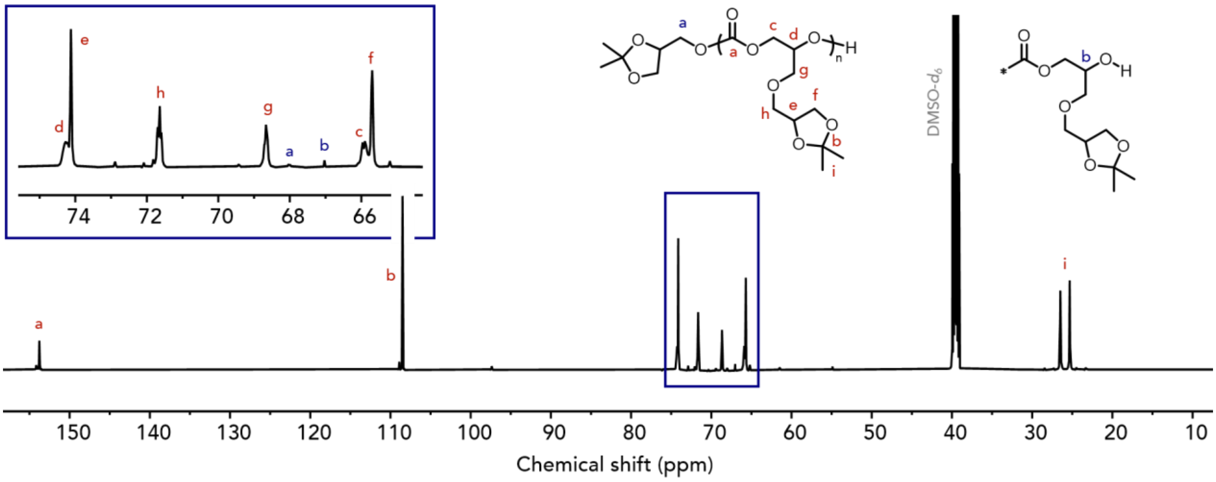

Figure S 21 – ^{13}C{^{1}H}-NMR spectrum (151 MHz, DMSO-d_6) of **P2**.

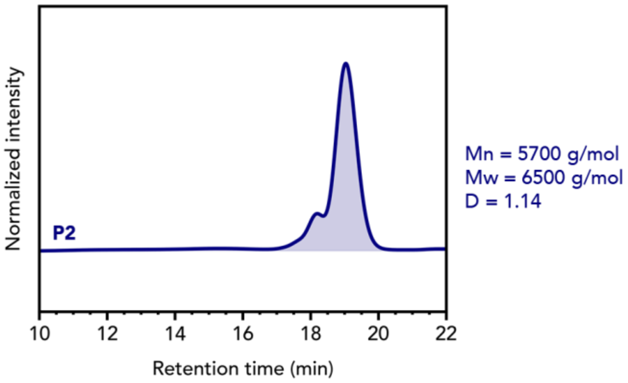

Figure S 22 – SEC trace (in THF) of **P2**.

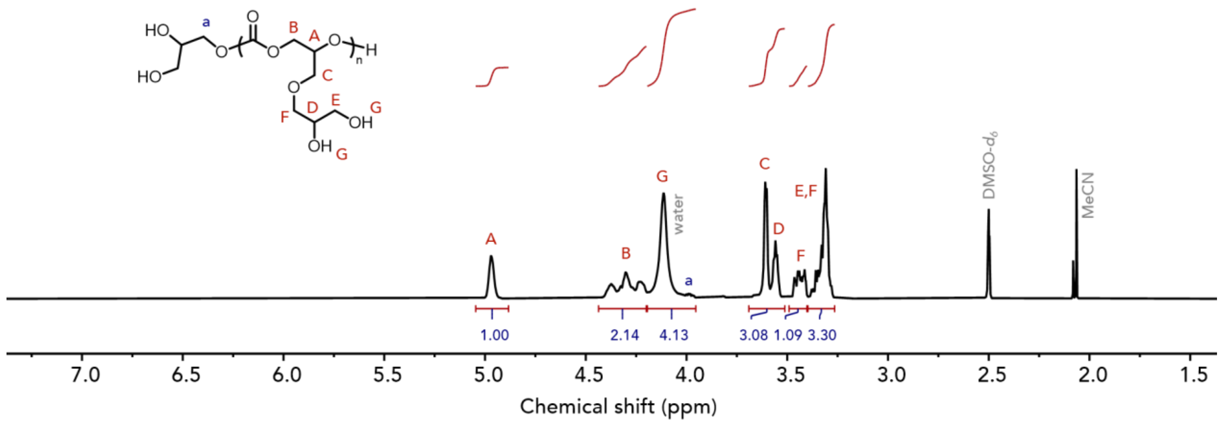

Figure S 23 – ^{1}H-NMR spectrum (600 MHz, DMSO-d_6) of **P2d**.

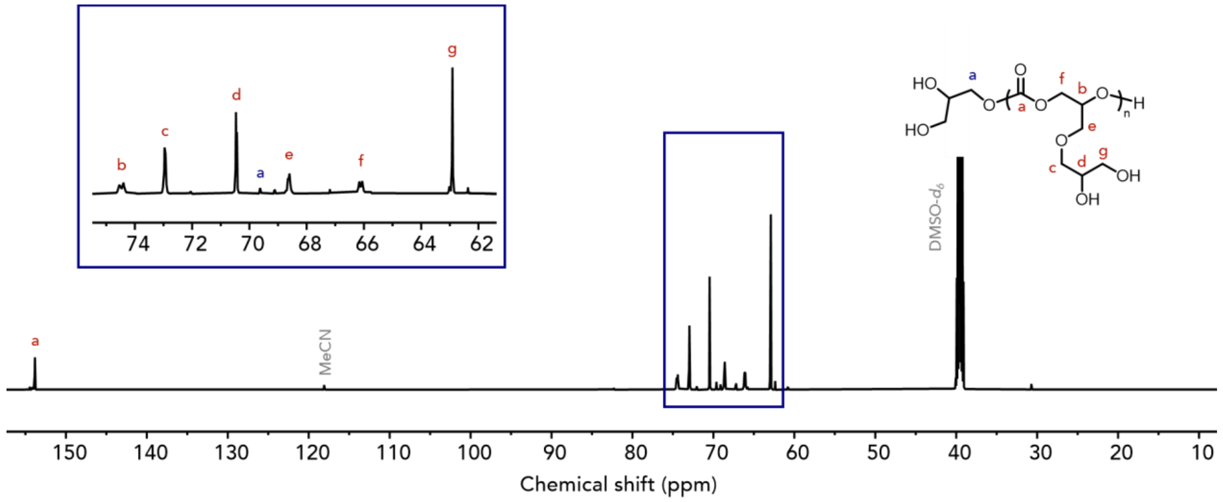

Figure S 24 – ^{13}C{^{1}H}-NMR spectrum (151 MHz, DMSO-d_6) of **P2d**.

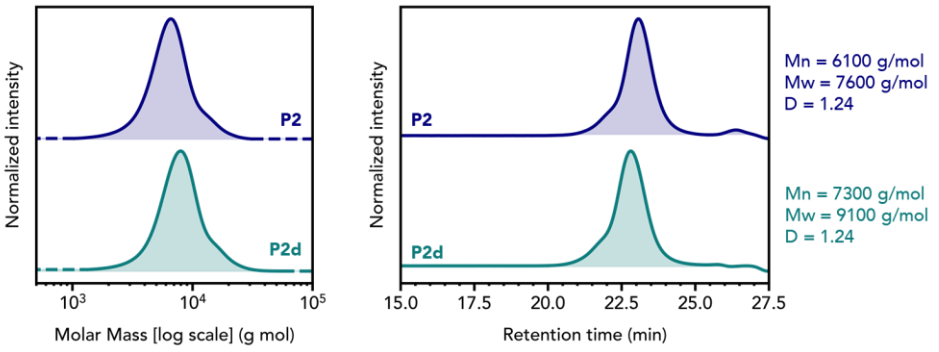

Figure S 25 – Stacked SEC traces (in DMF) of **P2** (top) and **P2d** (bottom) in term of molar mass (left) or retention time (right).

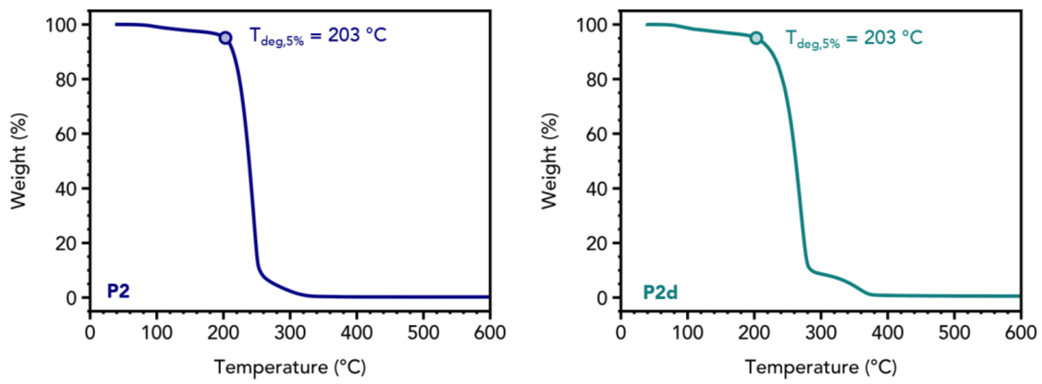

Figure S 26 – TGA data for **P2** and **P2d**.

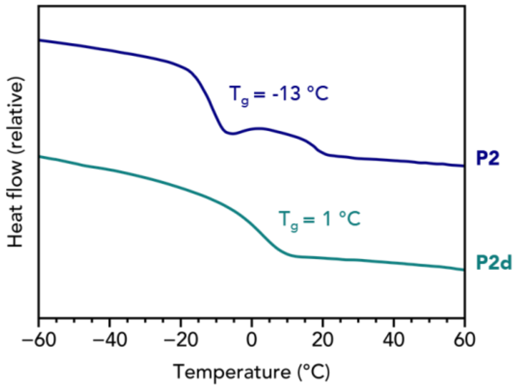

Figure S 27 – DSC data for **P2** and **P2d**.

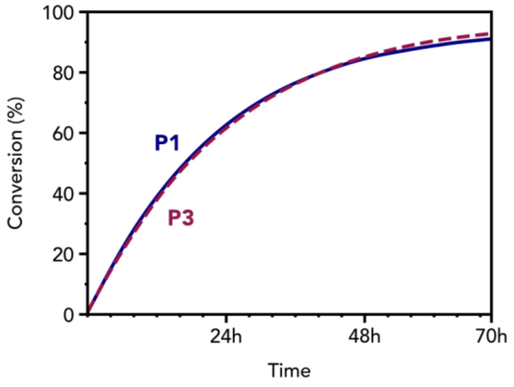

Figure S 28 – Plot of **IGG** conversion *vs* time for **P1** and **P3** using in-situ IR spectroscopy monitoring.

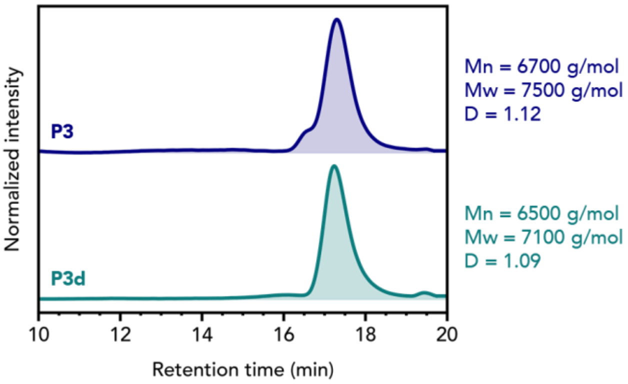

Figure S 29 – Stacked SEC traces (in DMF) of **P3** (top) and **P3d** (bottom).

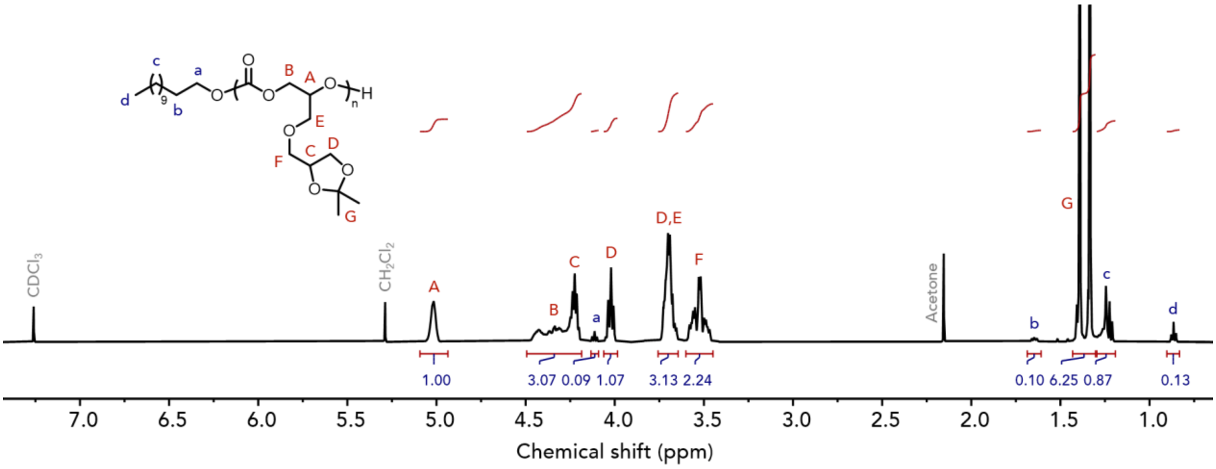

Figure S 30 – ^{1}H-NMR spectrum (500 MHz, $CDCl_3$) of **P3**.

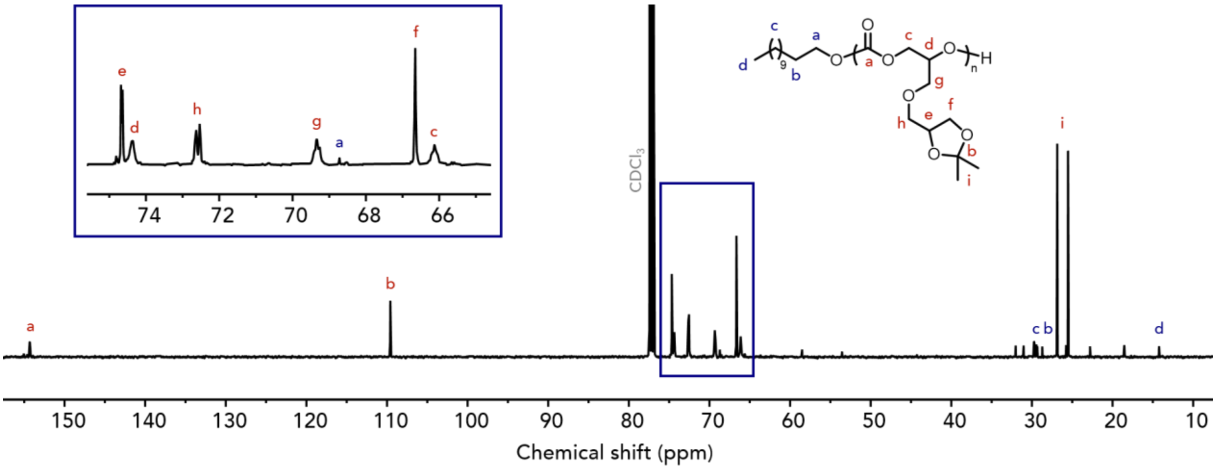

Figure S 31 – $^{13}C\{^{1}H\}$-NMR spectrum (126 MHz, $CDCl_3$) of **P3**.

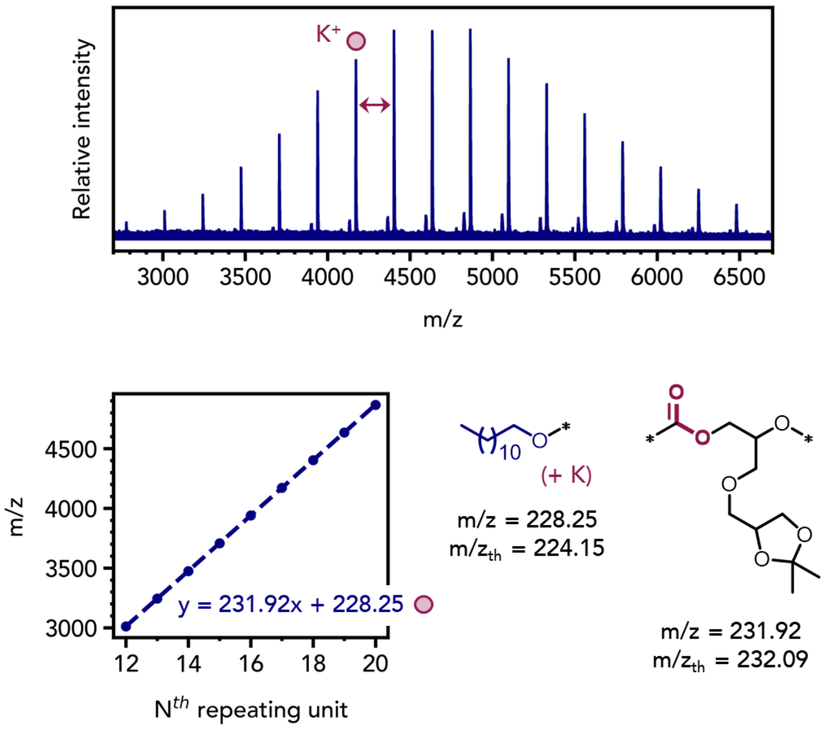

Figure S 32 – (A) MALDI-TOF spectrum of **P3**. (B) Plot of *m/z* vs N^th^ repeat unit. The theoretical molecular weights of the repeating unit and the end group match the experimental values.

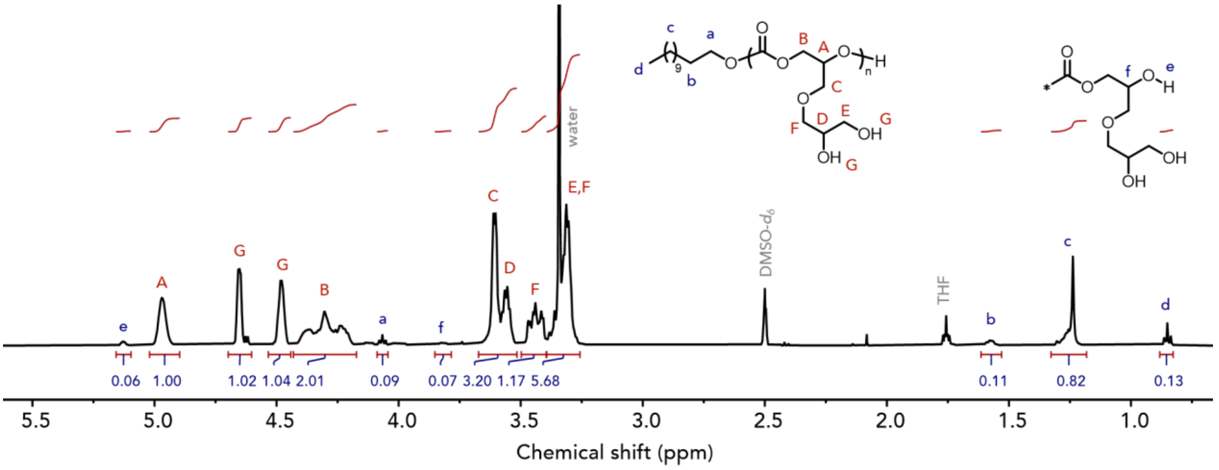

Figure S 33 – ^{1}H-NMR spectrum (500 MHz, DMSO-d_6) of **P3d**.

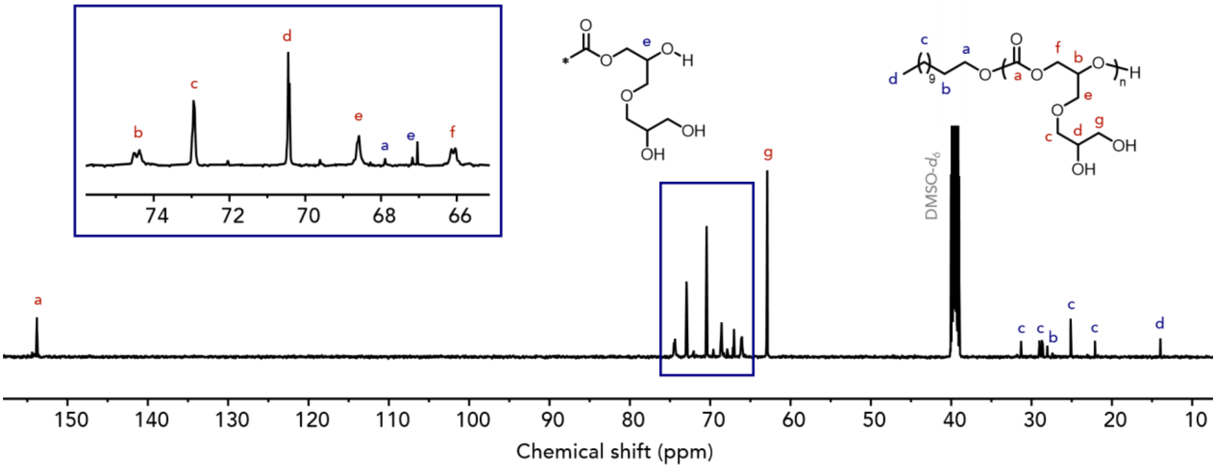

Figure S 34 – $^{13}C\{^1H\}$-NMR spectrum (126 MHz, DMSO-d_6) of **P3d**.

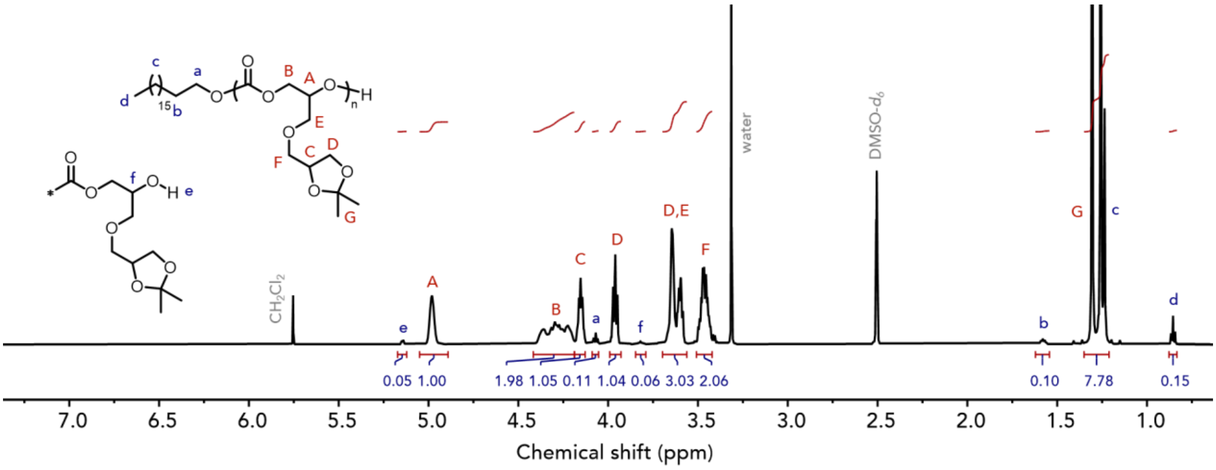

Figure S 35 – ^{1}H-NMR spectrum (600 MHz, DMSO-d_6) of **P4**.

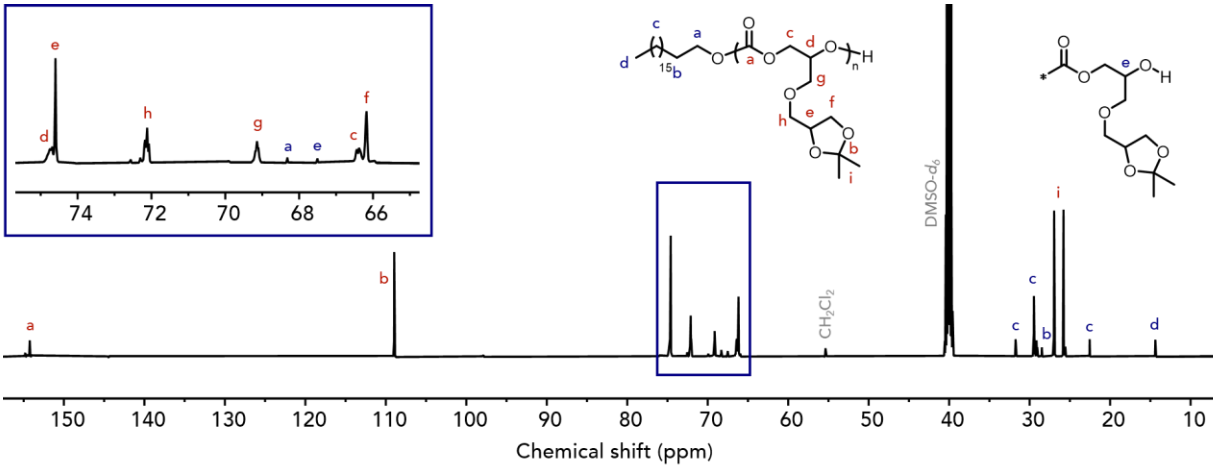

Figure S 36 – $^{13}C\{^1H\}$-NMR spectrum (151 MHz, DMSO-d_6) of **P4**.

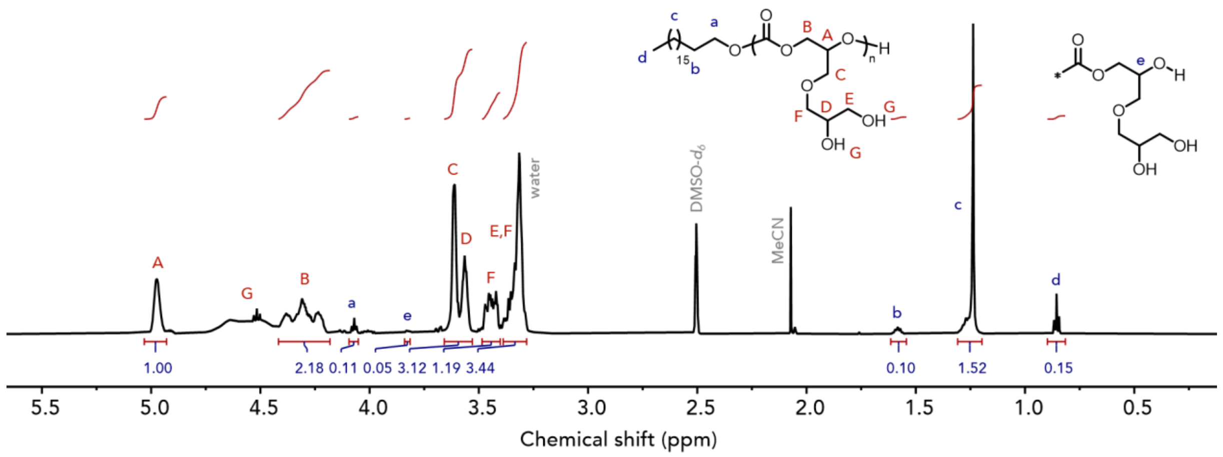

Figure S 37 – ^{1}H-NMR spectrum (600 MHz, DMSO-d_6) of **P4d**.

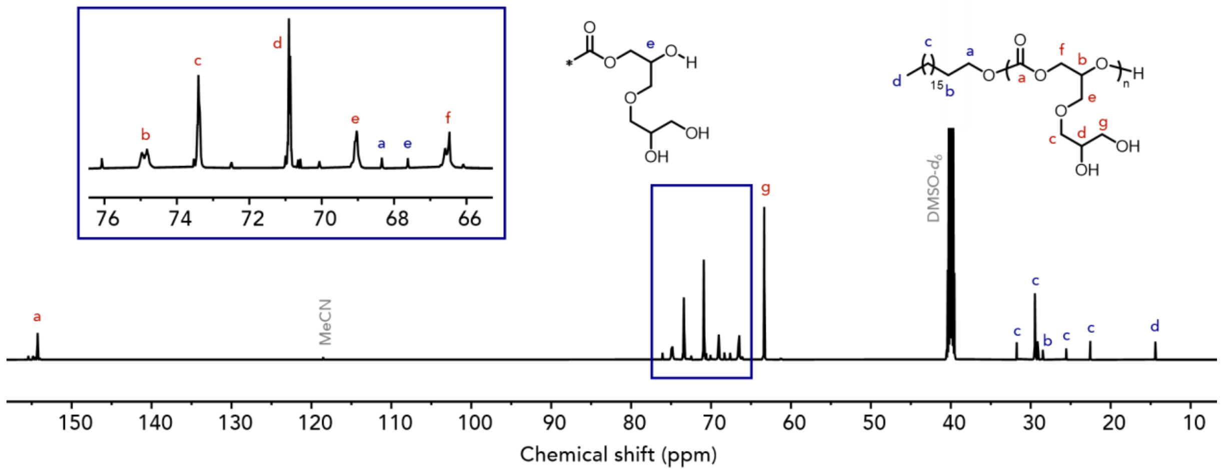

Figure S 38 – ^{13}C{^{1}H}-NMR spectrum (151 MHz, DMSO-d_6) of **P4d**.

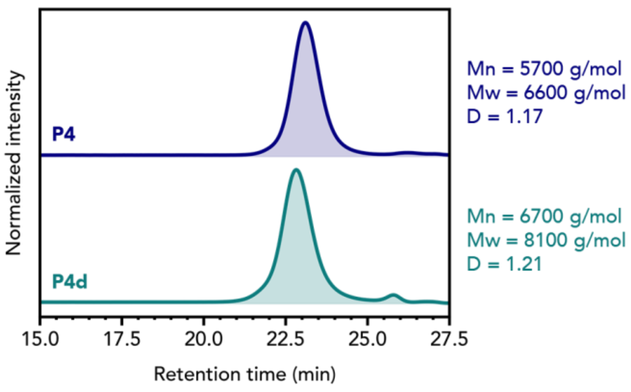

Figure S 39 – Stacked SEC traces (in DMF) of **P4** (top) and **P4d** (bottom).

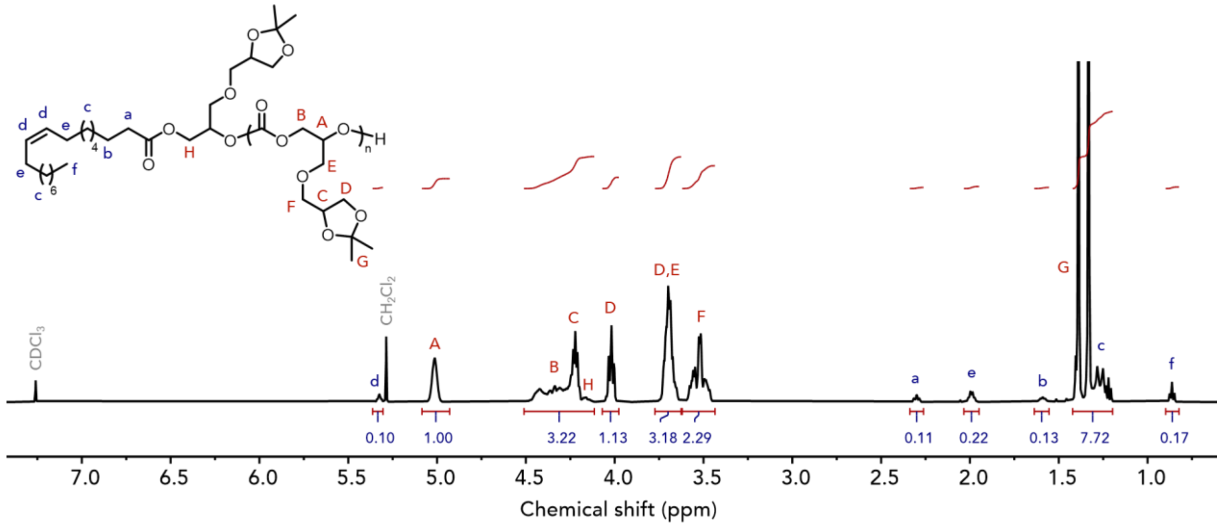

Figure S 40 – ^{1}H-NMR spectrum (500 MHz, $CDCl_3$) of **P5**.

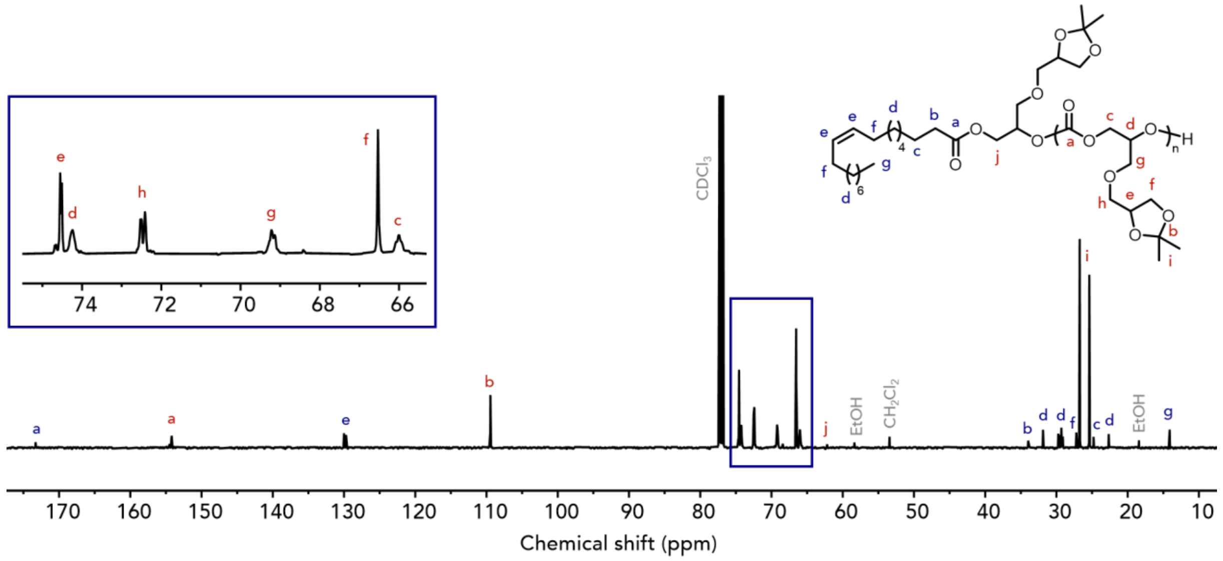

Figure S 41 – $^{13}C\{^{1}H\}$-NMR spectrum (126 MHz, $CDCl_3$) of **P5**.

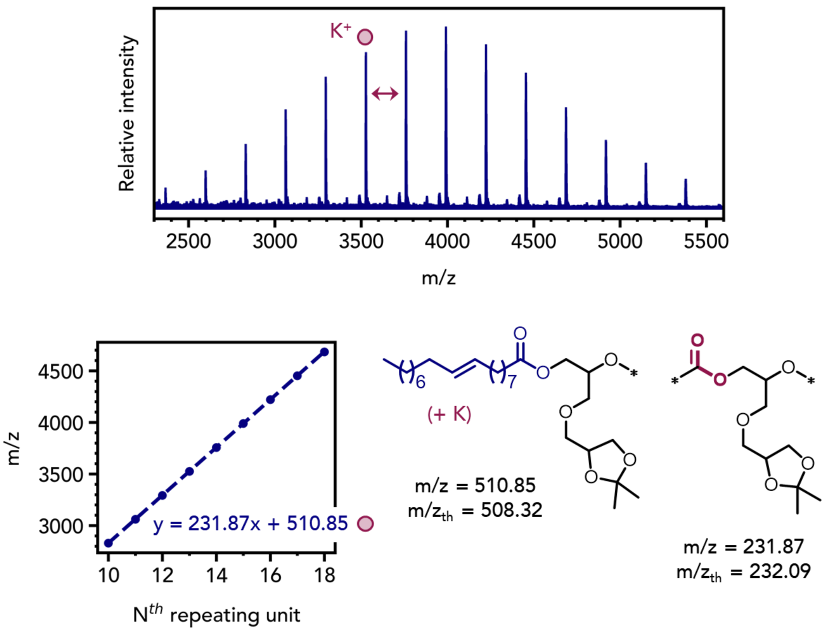

Figure S 42 – (A) MALDI-TOF spectrum of **P5**. (B) Plot of *m/z* vs Nth repeat unit. The theoretical molecular weights of the repeating unit and the end group match the experimental values.

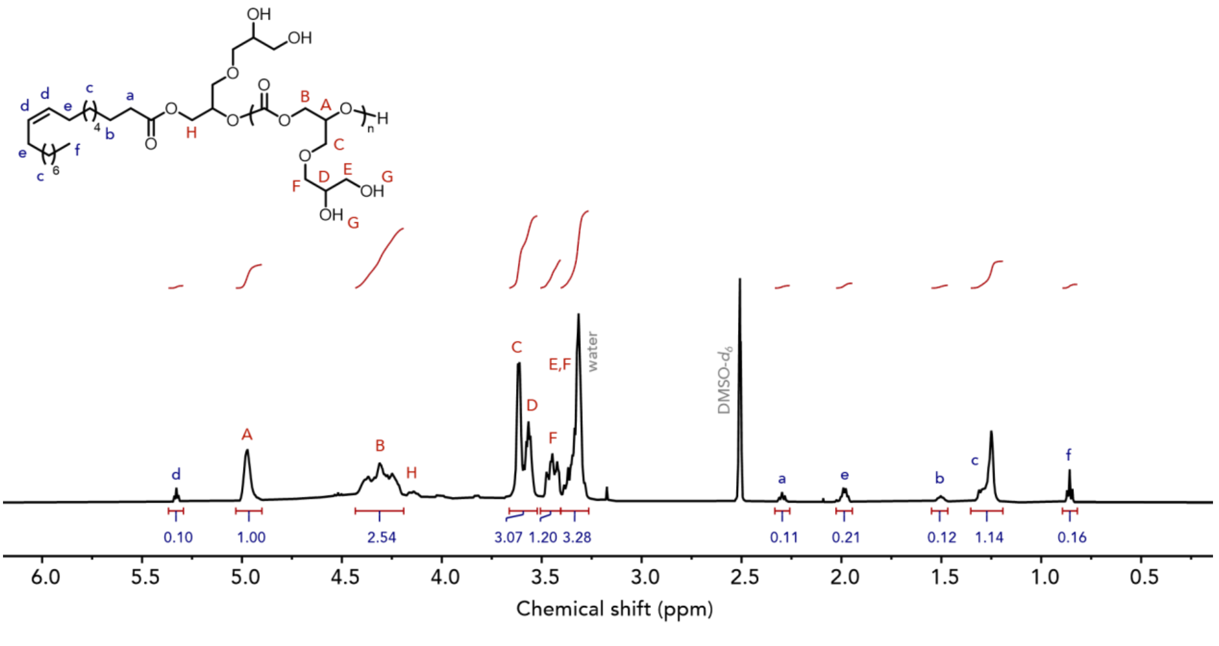

Figure S 43 – ^{1}H-NMR spectrum (500 MHz, DMSO-d_6) of **P5d**.

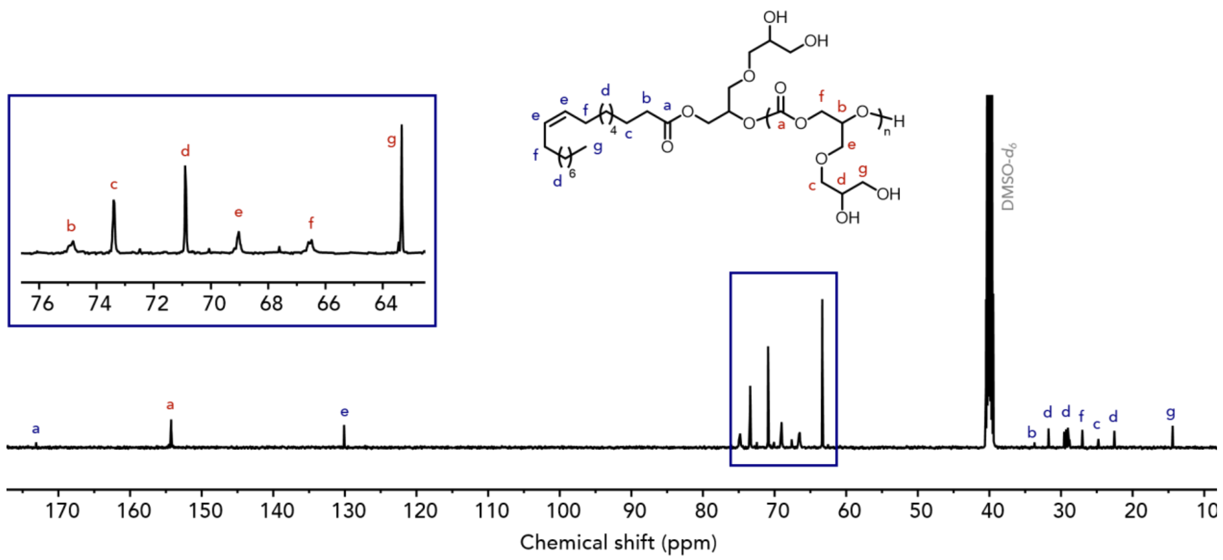

Figure S 44 – $^{13}C\{^{1}H\}$-NMR spectrum (126 MHz, DMSO-d_6) of **P5d**.

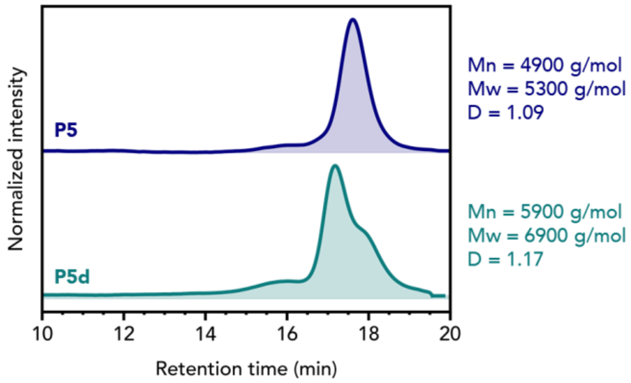

Figure S 45 – Stacked SEC traces (in DMF) of **P5** (top) and **P5d** (bottom).

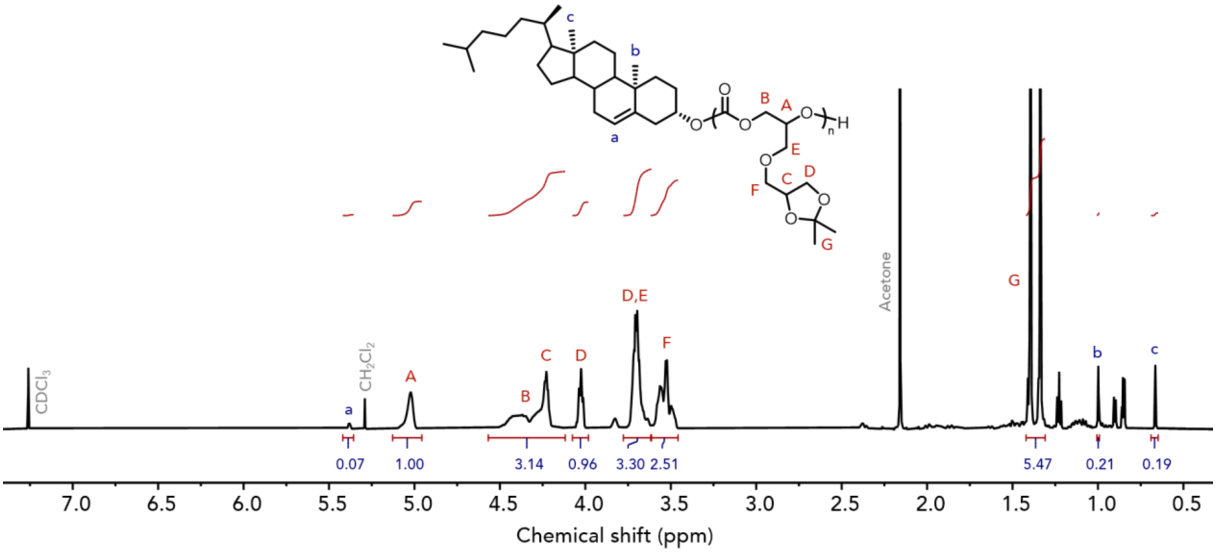

Figure S 46 – ^{1}H-NMR spectrum (600 MHz, $CDCl_3$) of **P6**.

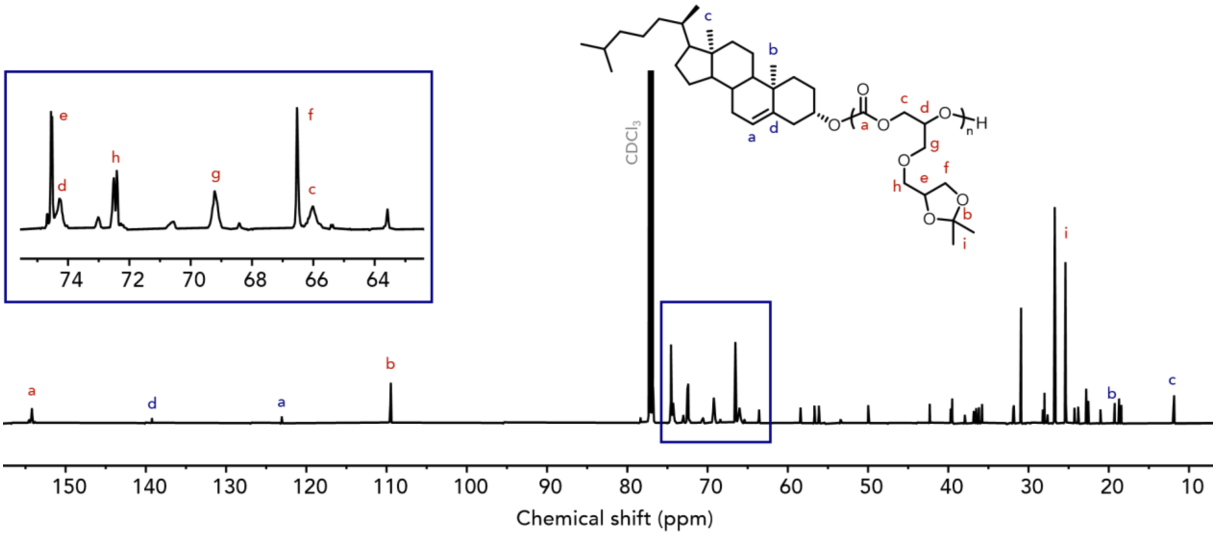

Figure S 47 – $^{13}C\{^1H\}$-NMR spectrum (151 MHz, $CDCl_3$) of **P6**.

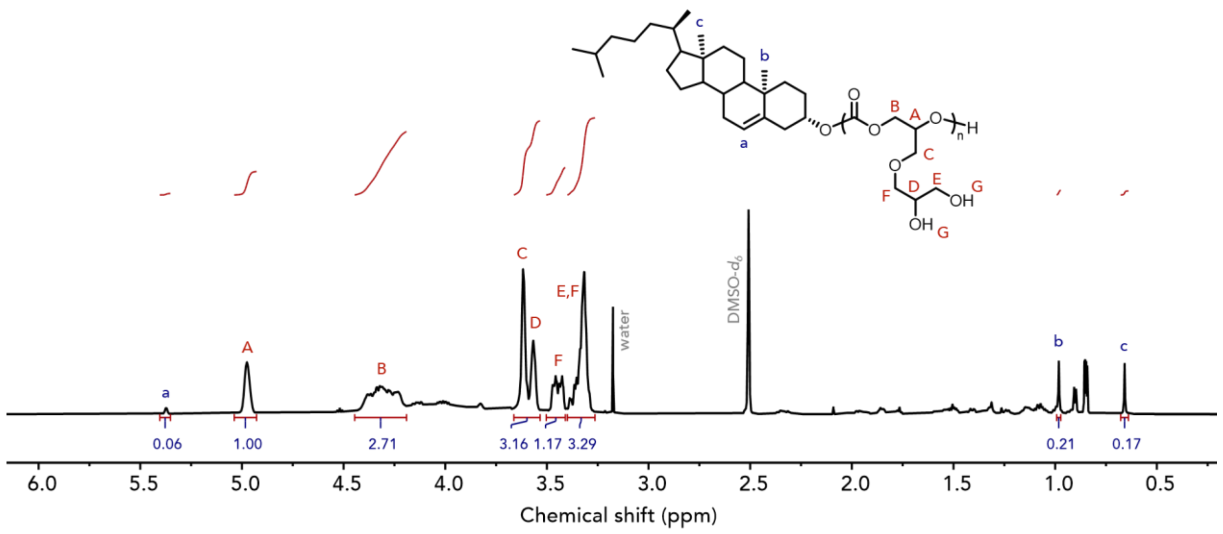

Figure S 48 – ^{1}H-NMR spectrum (700 MHz, DMSO-d_6) of **P6d**.

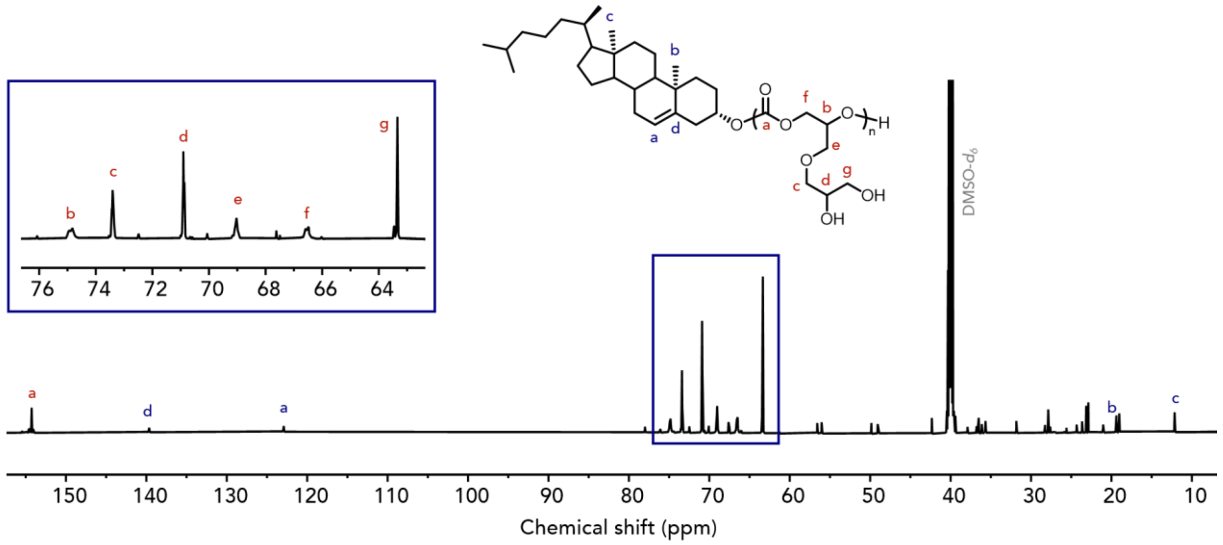

Figure S 49 – $^{13}C\{^{1}H\}$-NMR spectrum (176 MHz, DMSO-d_6) of **P6d**.

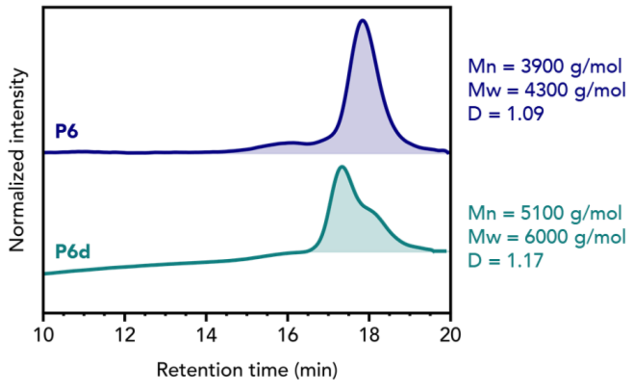

Figure S 50 – Stacked SEC traces (in DMF) of **P6** (top) and **P6d** (bottom).

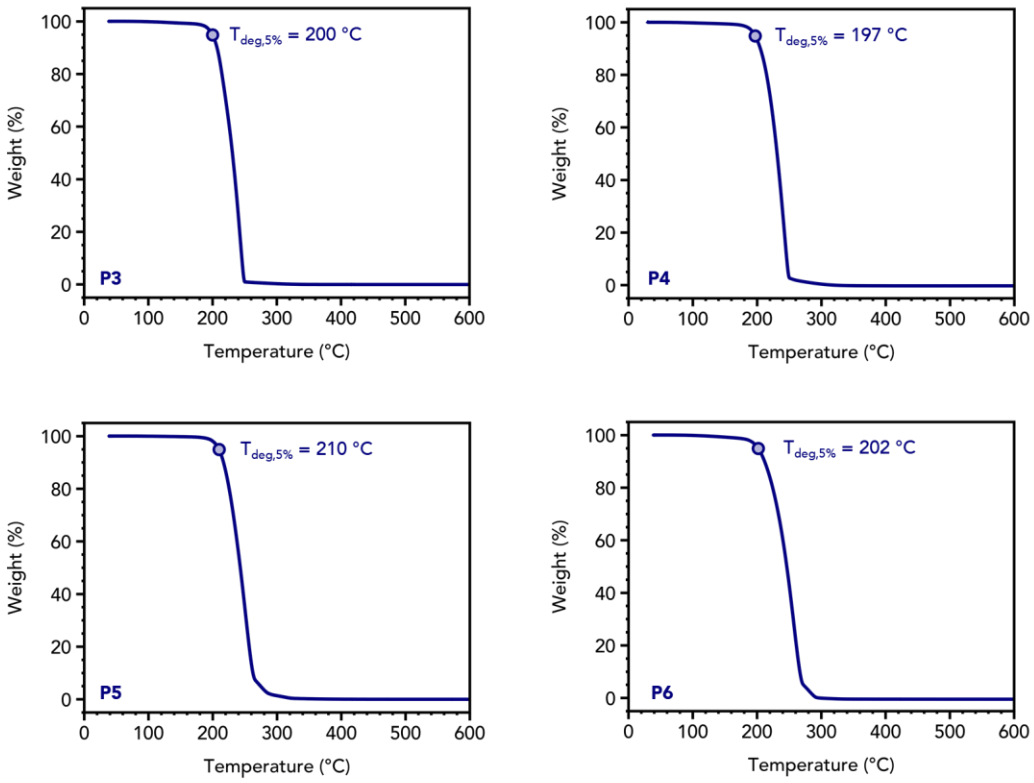

Figure S 51 – TGA data for **P3**, **P4**, **P5** and **P6**.

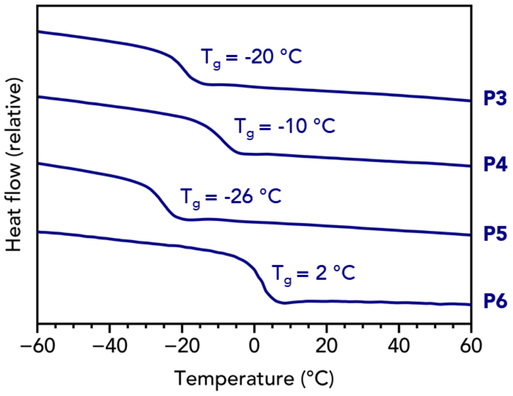

Figure S 52 – DSC data for **P3**, **P4**, **P5** and **P6**.

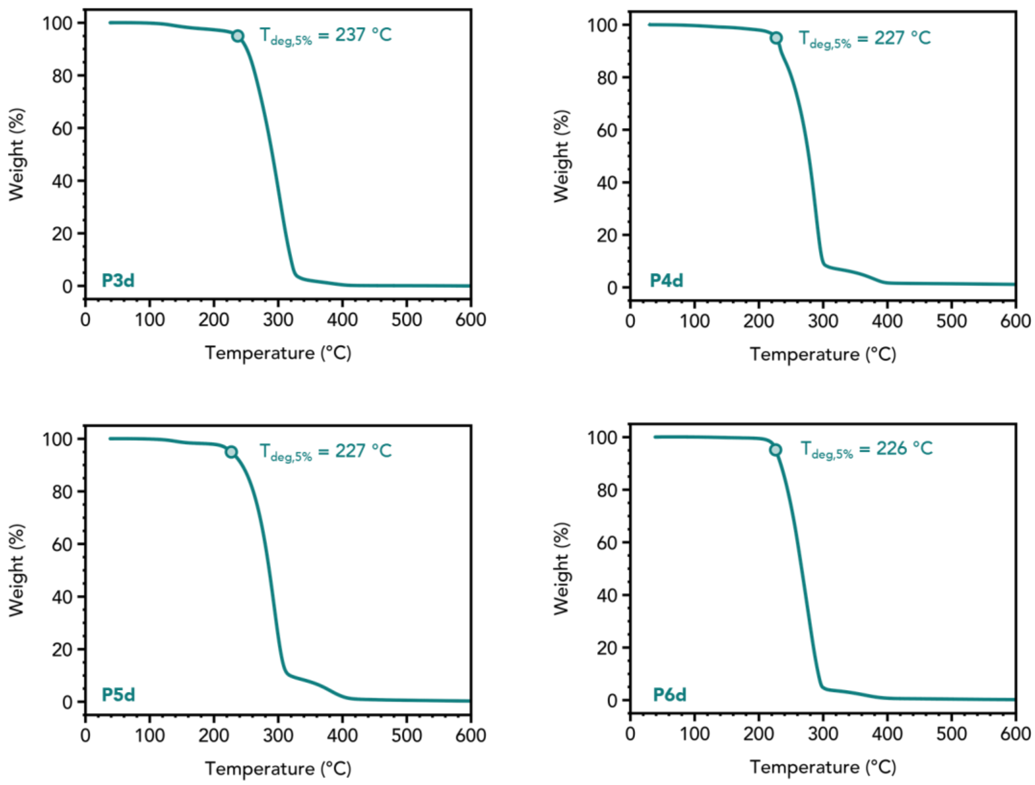

Figure S 53 – TGA data for **P3d**, **P4d**, **P5d** and **P6d**.

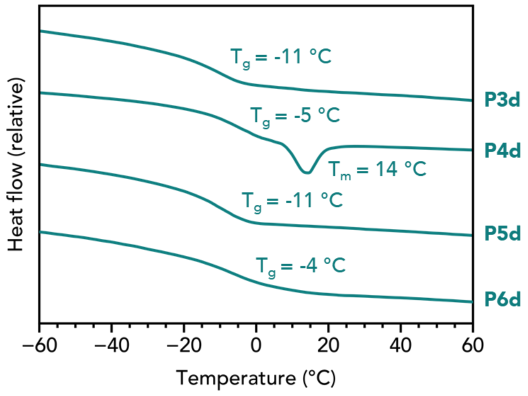

Figure S 54 – DSC data for **P3d**, **P4d**, **P5d** and **P6d**.

<u>CryoTEM imaging of P4d</u>

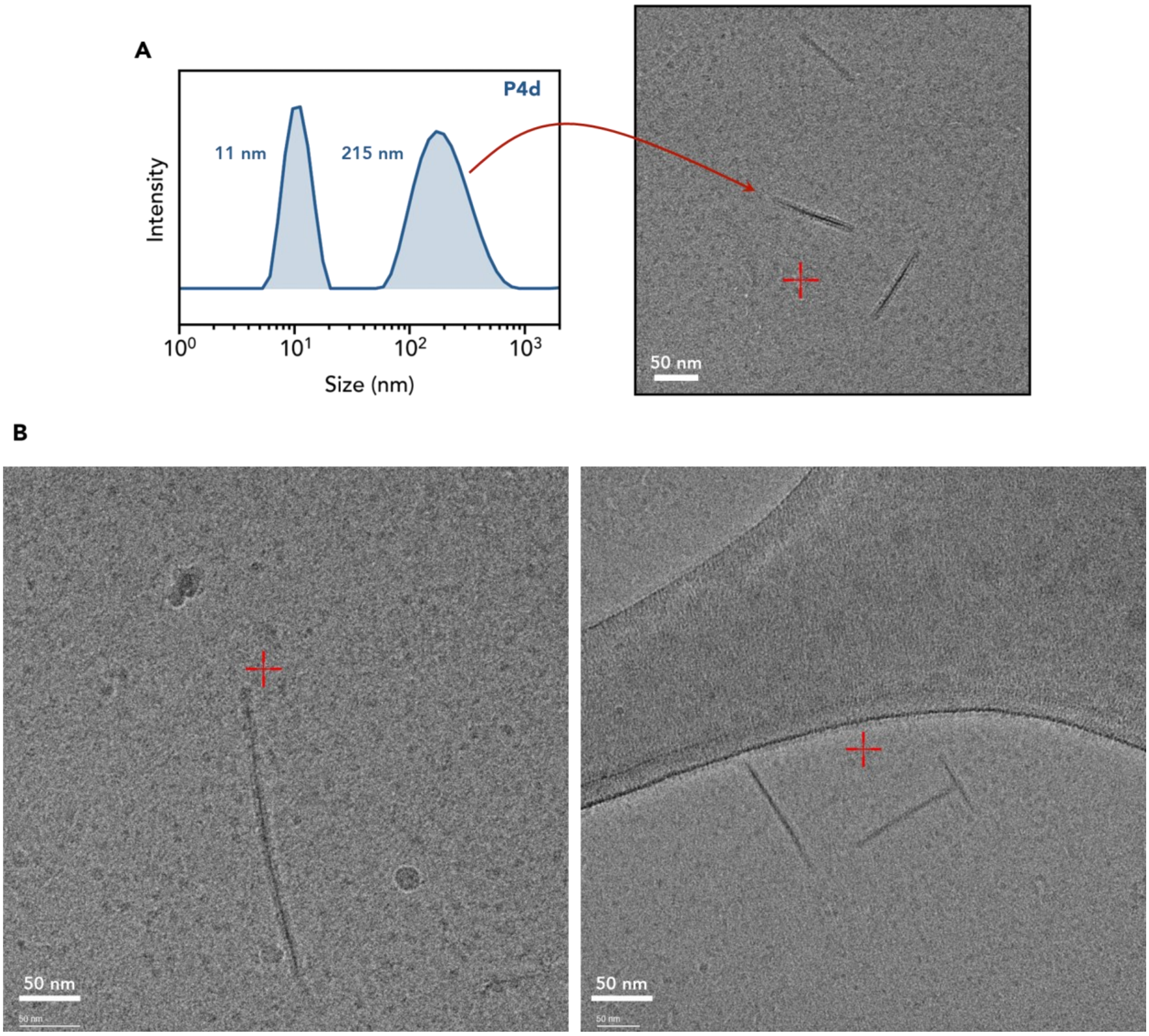

Figure S 55 – (A) DLS particle size distribution recorded for a 10 mg mL^{-1} aqueous solution of the polymer **P4d** (same as in Figure 5). A CryoTEM image of the same solution of **P4d** shows rod-like assemblies, which is consistent with the minor population of larger particles with a hydrodynamic sphere-equivalent diameter of 215 nm detected by DLS. (B) Additional images from the same CryoTEM grid. These rods have lengths of 70–230 nm and mean diameters of 6–13 nm.

Surface tensiometry of P4d

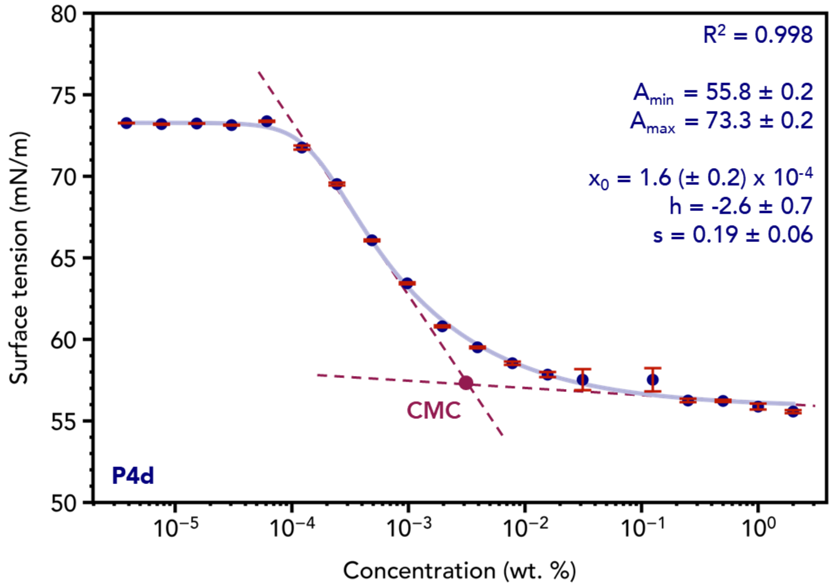

Figure S 56 – Aqueous surface tension vs. concentration plots obtained for polymer **P4d**. The raw data was fitted to a 5-parameter logistic function.

Degradation study and characterization of compounds 1 and 2, and polymers P1c and P1cd

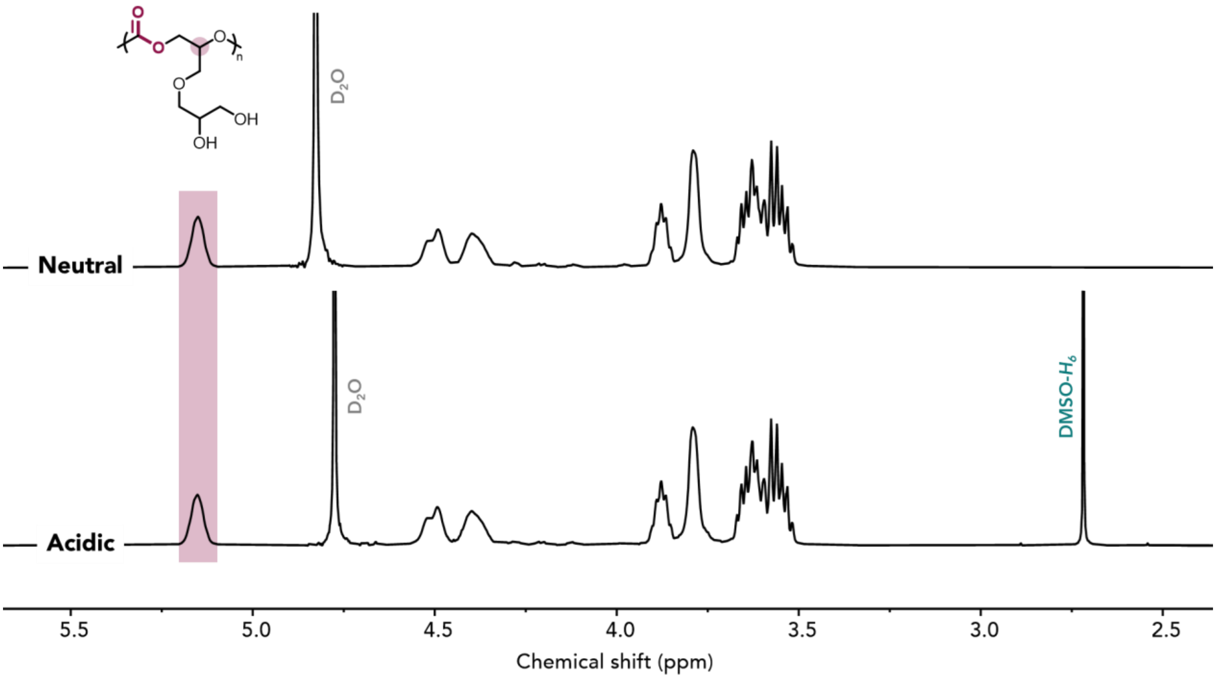

Figure S 57 – Stacked ^{1}H-NMR spectra (400 MHz, D_2O) of a 25 mg mL^{-1} solution of **P2d** dissolved in neutral D_2O (top) and acidified D_2O by addition of 1 % (v/v) D_2SO_4 (bottom). DMSO-H_6 was added to the acidic solution to track degradation. No changes were observed in both cases after one month.

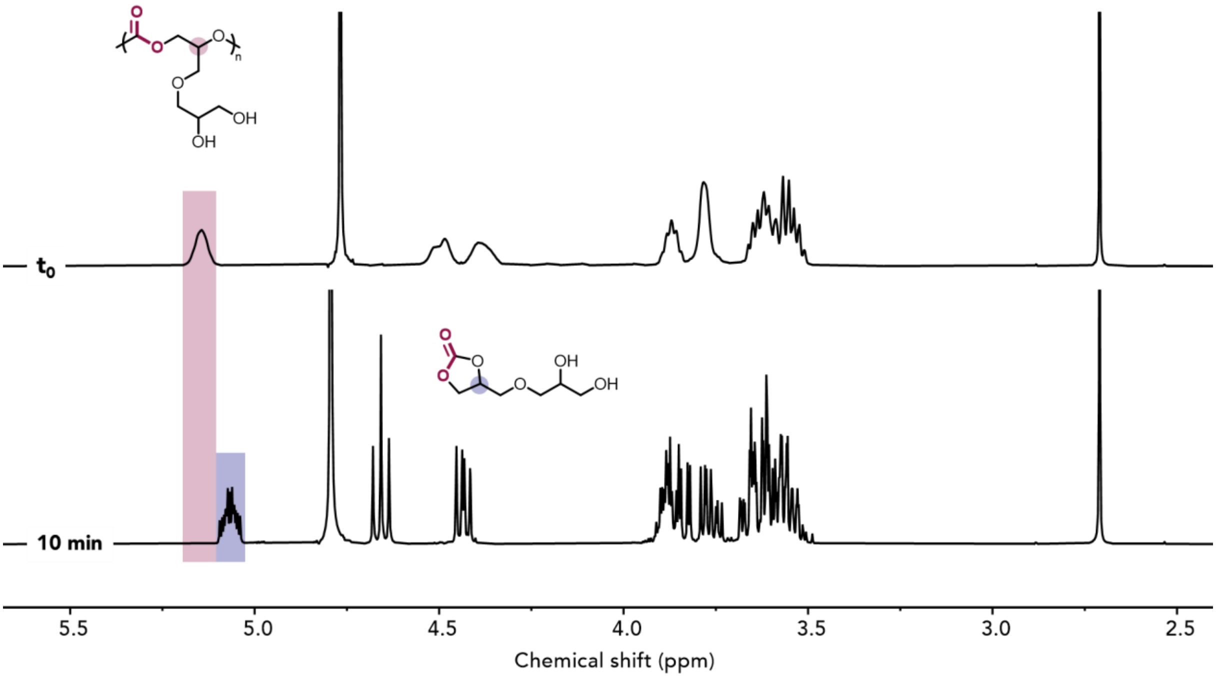

Figure S 58 – Stacked ^{1}H-NMR spectra (400 MHz, D_2O) of the crude reaction medium at t_0 (top) and 10 min (bottom) for the degradation of **P2d** at pH 10.

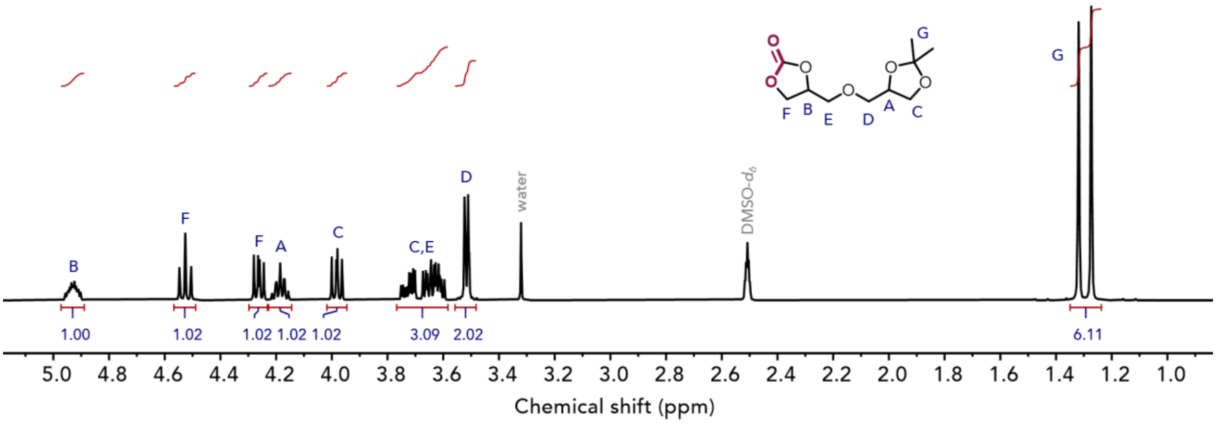

Figure S 59 – ^{1}H-NMR spectrum (400 MHz, DMSO-d_6) of **1**.

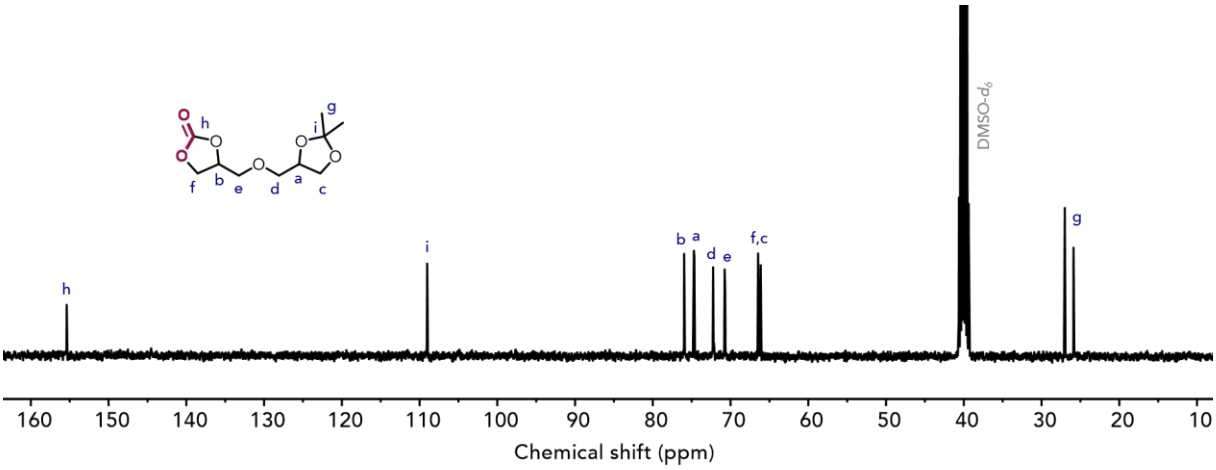

Figure S 60 – ^{13}C{^{1}H}-NMR spectrum (101 MHz, DMSO-d_6) of **1**.

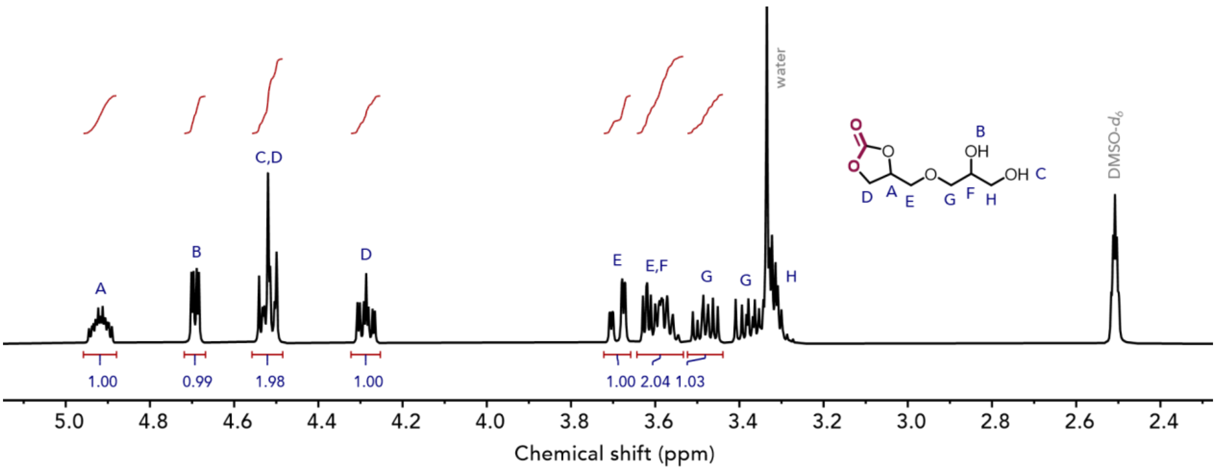

Figure S 61 – ^{1}H-NMR spectrum (400 MHz, DMSO-d_6) of **2**.

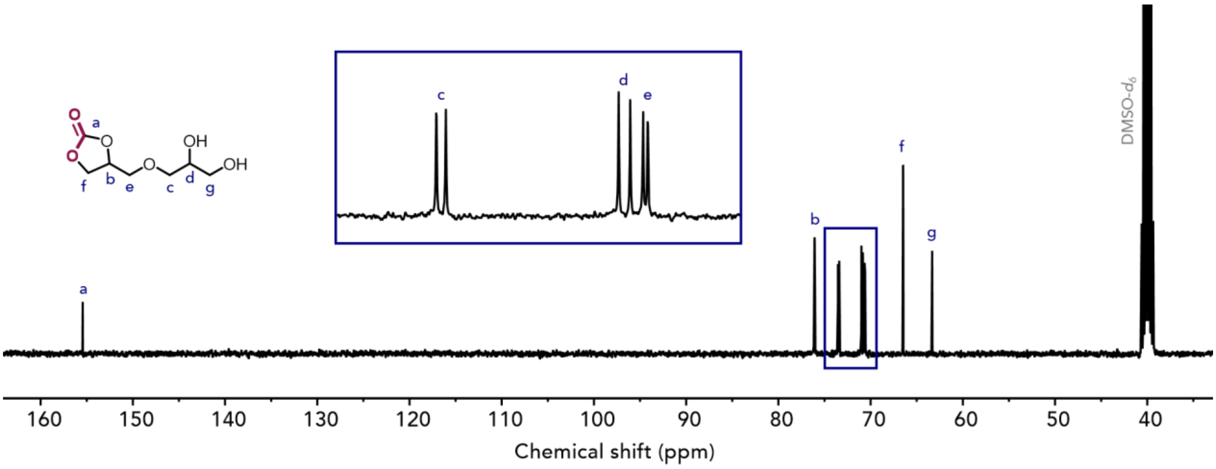

Figure S 62 – $^{13}C\{^1H\}$-NMR spectrum (101 MHz, DMSO-d_6) of **2**.

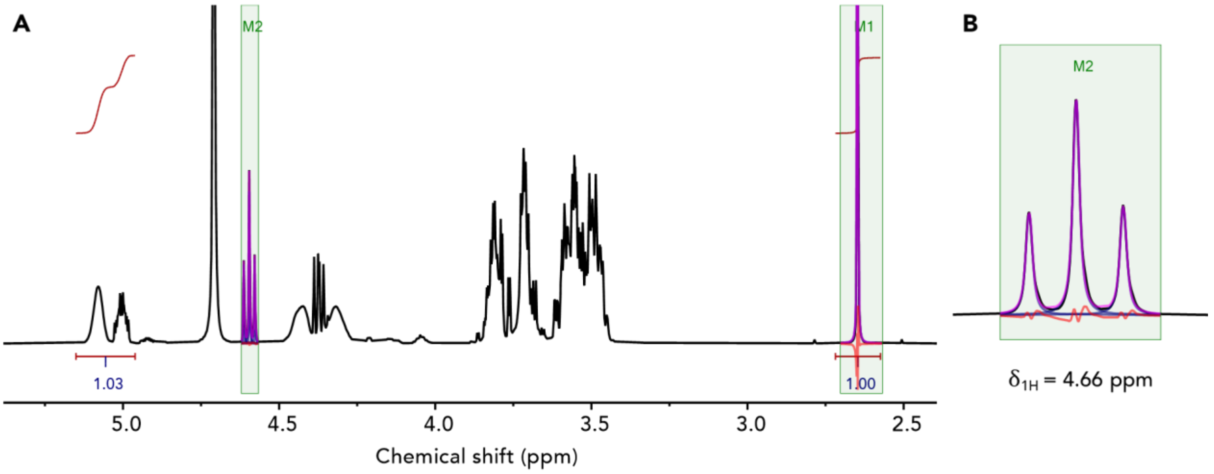

Figure S 63 – (A) Representative ^{1}H-NMR spectrum (400 MHz, D_2O) of the crude reaction medium (at t = 2h) for the degradation of **P2d** at pH 8. (B) Peak fitting was achieved on the well-defined triplet (4.66 ppm) to get accurate integration.

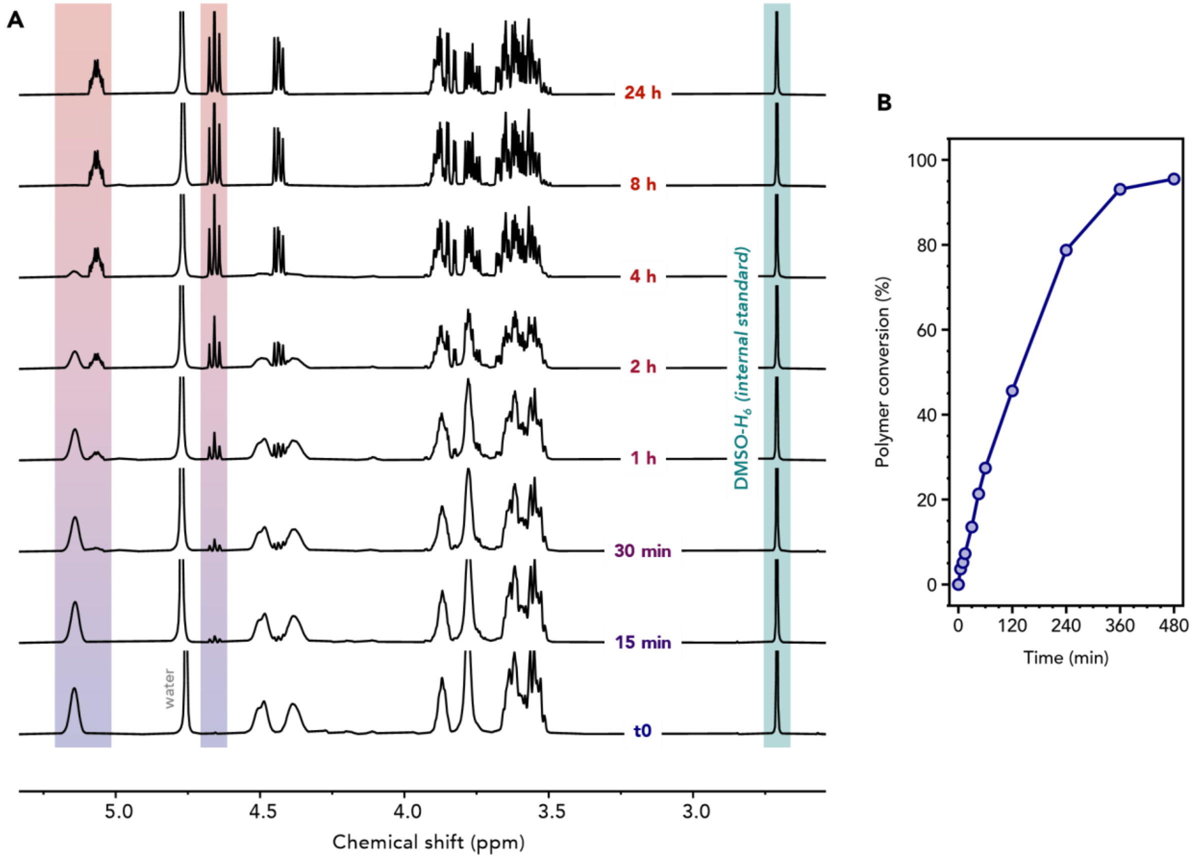

Figure S 64 – (A) Stacked ^{1}H-NMR spectra (400 MHz, D_2O) of the crude reaction medium for the degradation of **P2d** at pH 8. Over time (from bottom to top), the typical resonance of the polymer (5.14 ppm) disappears while typical resonances of product **2** appear (5.07 and 4.66 ppm). (B) Plot of the polymer linkage conversion *vs* time for the degradation of **P2d**.

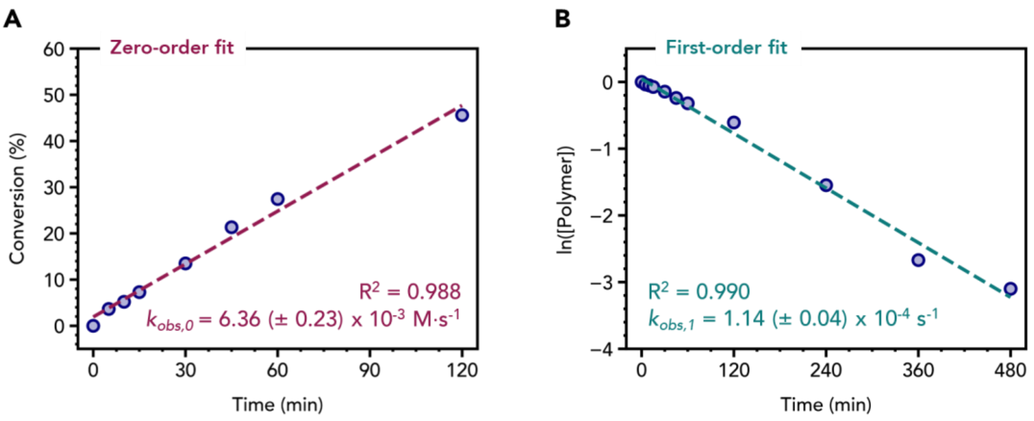

Figure S 65 – Plots used to monitor the kinetics of degradation of **P2d**: (Left) Plot of the polymer linkage conversion *vs* time until half conversion. A zero-order was observed in this regime and a rate constant k_{zero} could be extracted from a linear fit. (Right) Plot of ln([Polymer]) *vs* time over a broader timescale. A first-order dependence on polymer concentration was observed and a rate constant k_{first} could be extracted from a linear fit.

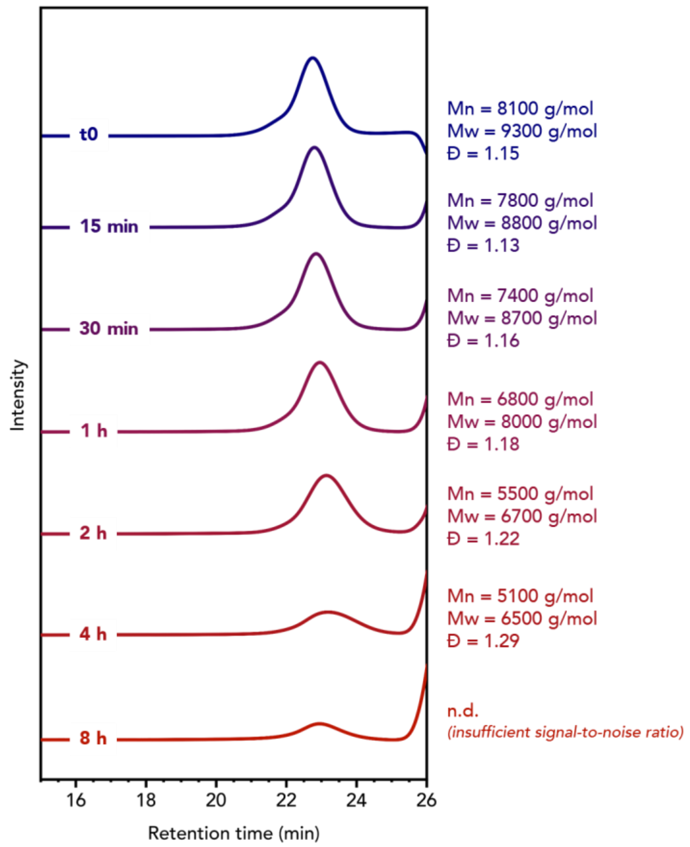

Figure S 66 – Stacked SEC traces (in DMF) for the degradation of **P2d** along time (pH 8).

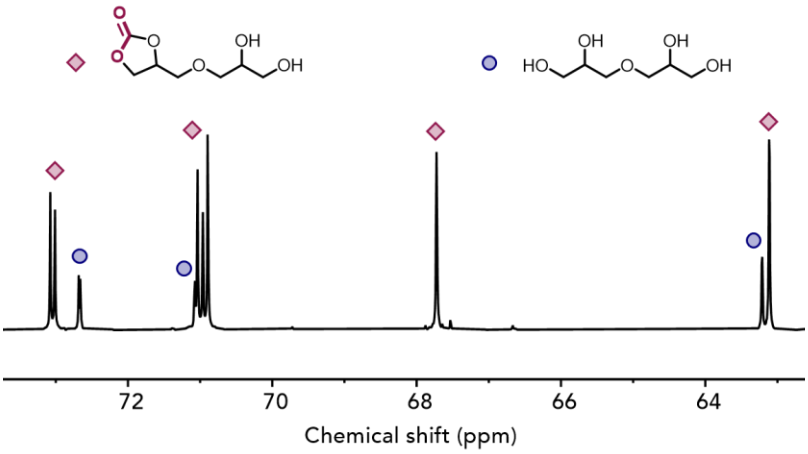

Figure S 67 – $^{13}C\{^1H\}$-NMR spectrum (151 MHz, D_2O) of the crude reaction medium at the end of degradation of **P2d**.

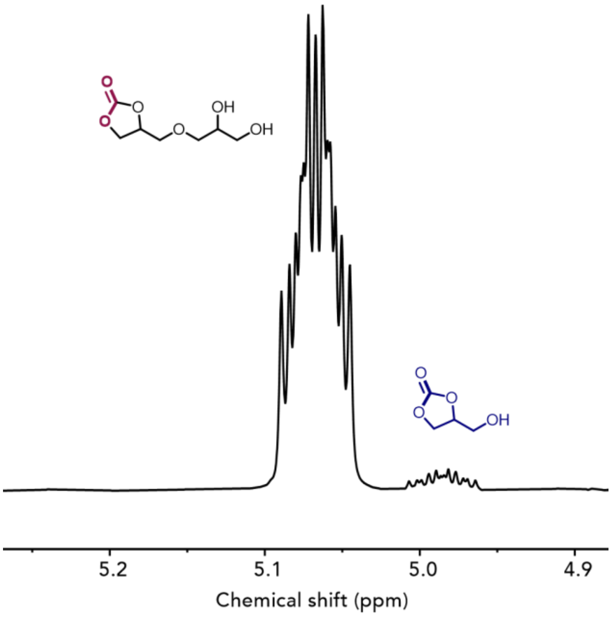

Figure S 68 – Zoomed ^1H-NMR spectrum (400 MHz, D_2O) of the crude reaction medium (at t = 24h) for the degradation of **P2d** at pH 8. A small peak corresponding to glycerol carbonate is visible close to the peak corresponding to **2**.

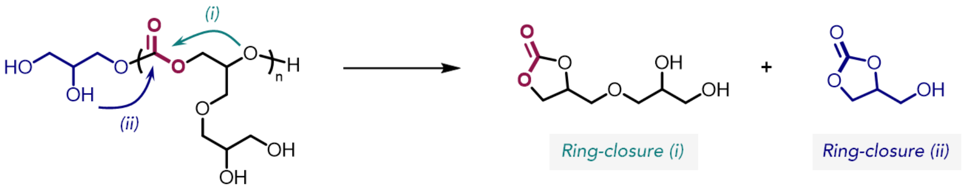

Scheme S 2 – The degradation of **P2d** leads to two cyclic carbonates. A first ring-closure (i) from the hydroxyl end -group leads to **2**, and a second ring-closure (ii) from the hydroxyl of the deprotected solketal chain-end leads to glycerol carbonate.

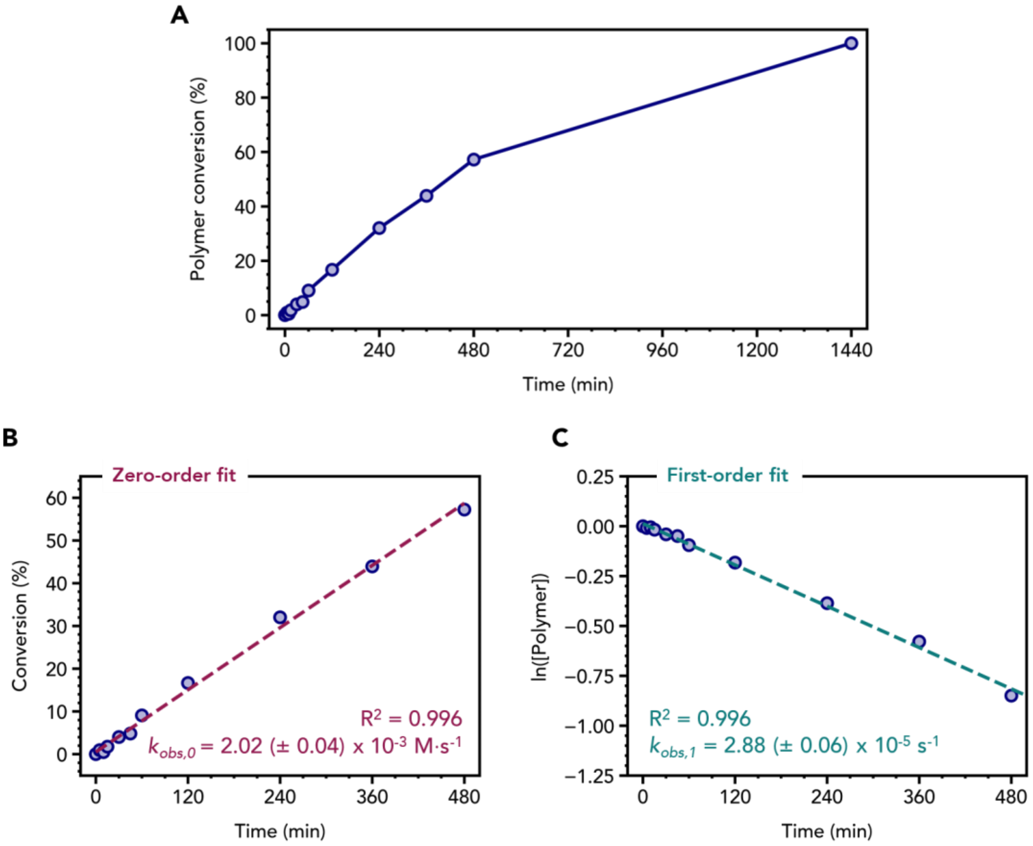

Figure S 69 – Plots used to monitor the kinetics of degradation of **P4d**: (A) Complete plot of the polymer linkage conversion *vs* time for the degradation of **P4d**. (B) Plot of the polymer linkage conversion *vs* time until half conversion. A zero-order was observed in this regime and a rate constant k_{zero} could be extracted from a linear fit. (C) Plot of ln([Polymer]) *vs* time over a broader timescale. A first-order dependence on polymer concentration was observed and a rate constant k_{first} could be extracted from a linear fit.

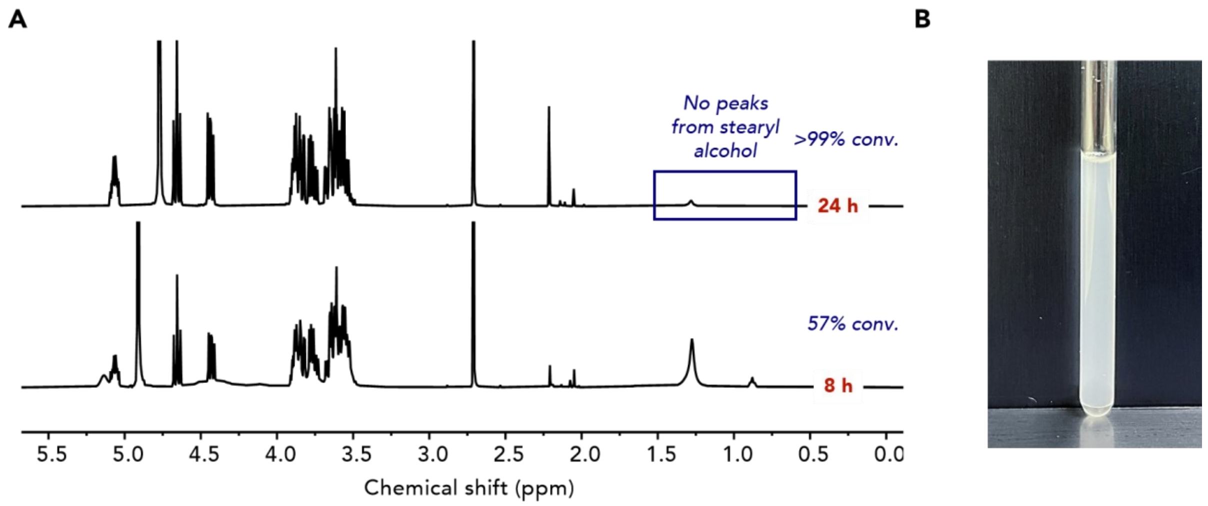

Figure S 70 – (A) Stacked ^{1}H-NMR spectra (400 MHz, D_2O) of the crude reaction medium for the degradation of **P4d** at pH 8 after 8 h and 24 h. (B) Photograph of the NMR tube prepared from the crude of reaction after 24 h: an opaque colloid is observed.

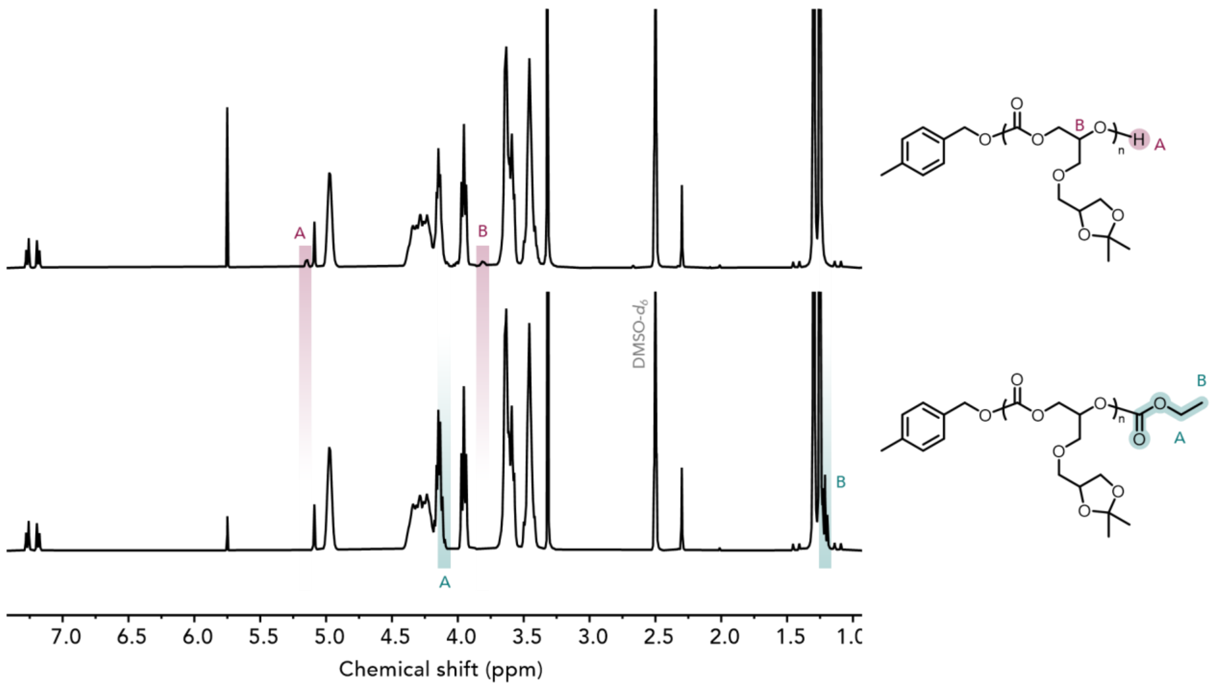

Figure S 71 – Stacked ^{1}H-NMR spectra (400 MHz, DMSO-d_6) of **P1** (top) and **P1c** (bottom).

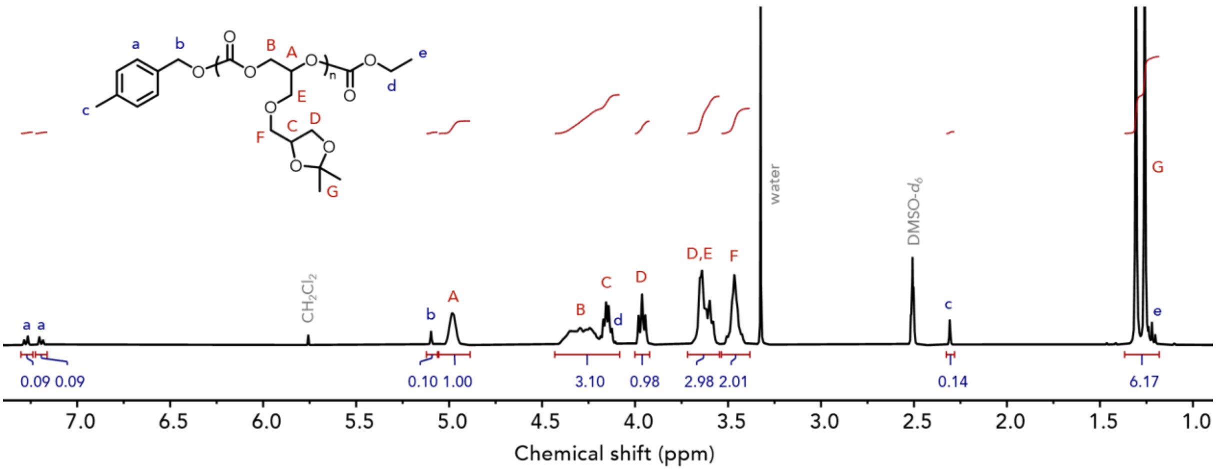

Figure S 72 – ^{1}H-NMR spectrum (400 MHz, DMSO-d_6) of **P1c**.

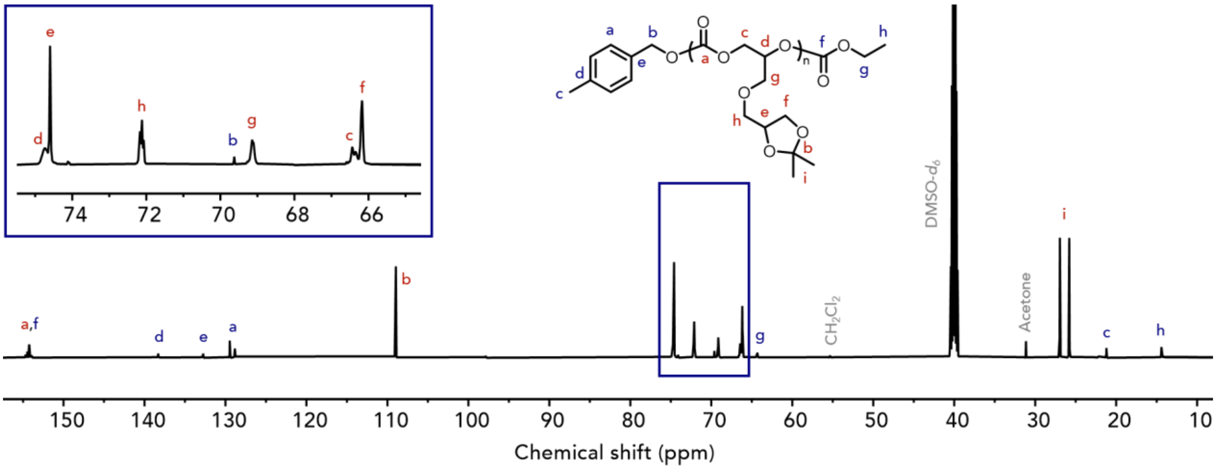

Figure S 73 – ^{13}C{^{1}H}-NMR spectrum (151 MHz, DMSO-d_6) of **P1c**.

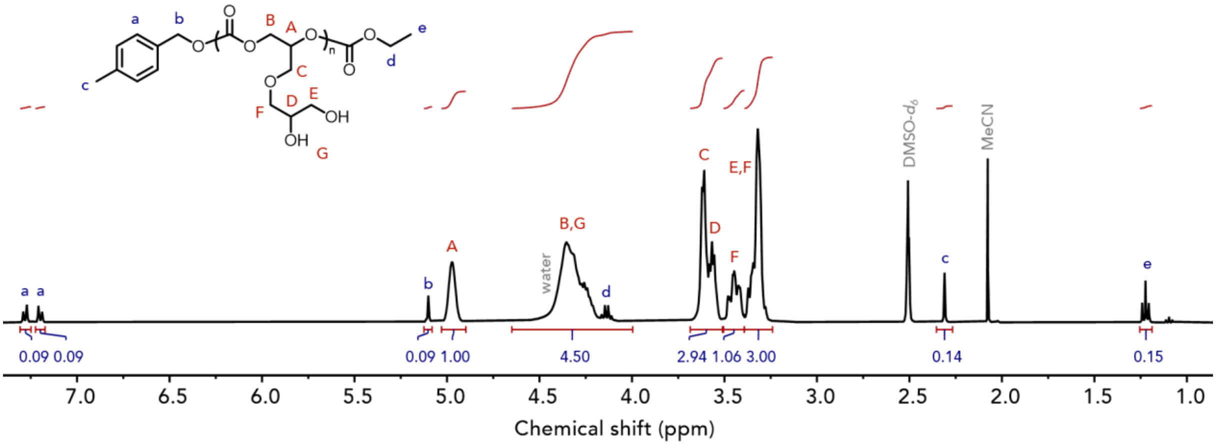

Figure S 74 – ^{1}H-NMR spectrum (400 MHz, DMSO-d_6) of **P1cd**.

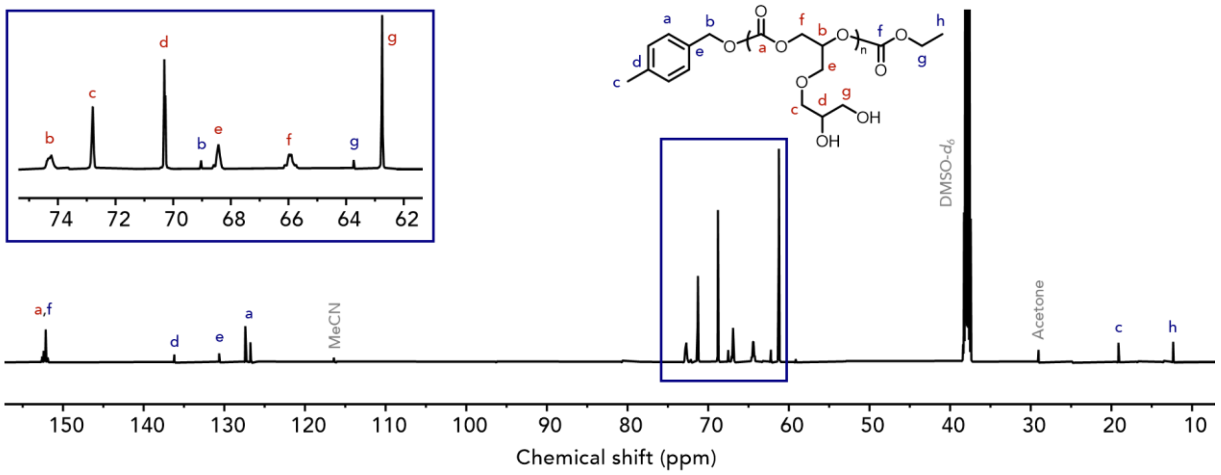

Figure S 75 – $^{13}C\{^1H\}$-NMR spectrum (151 MHz, DMSO-d_6) of **P1cd**.

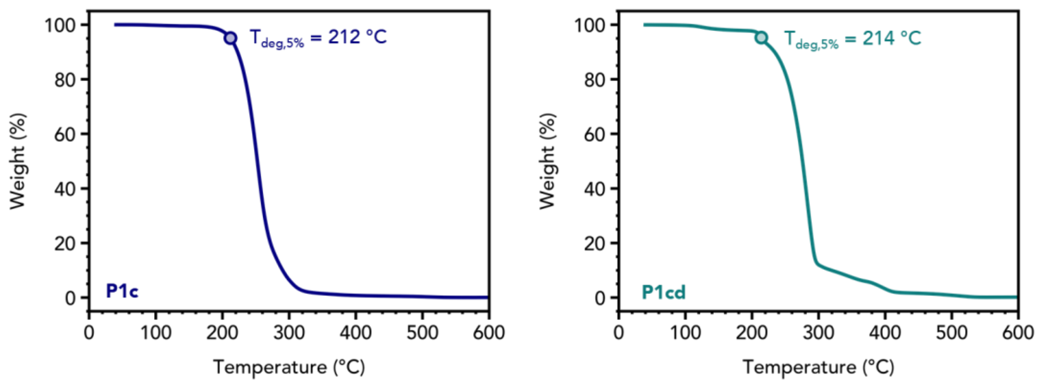

Figure S 76 – TGA data for **P1c** and **P1cd**.

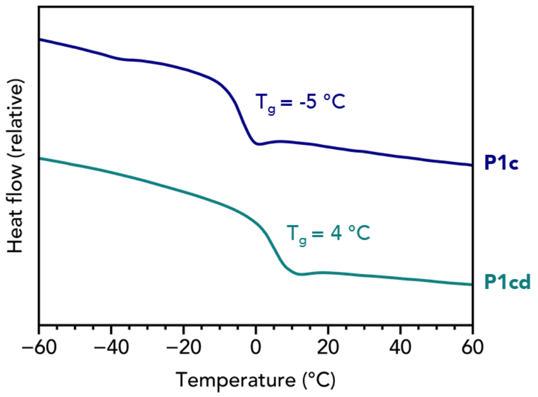

Figure S 77 – DSC data for **P1c** and **P1cd**.

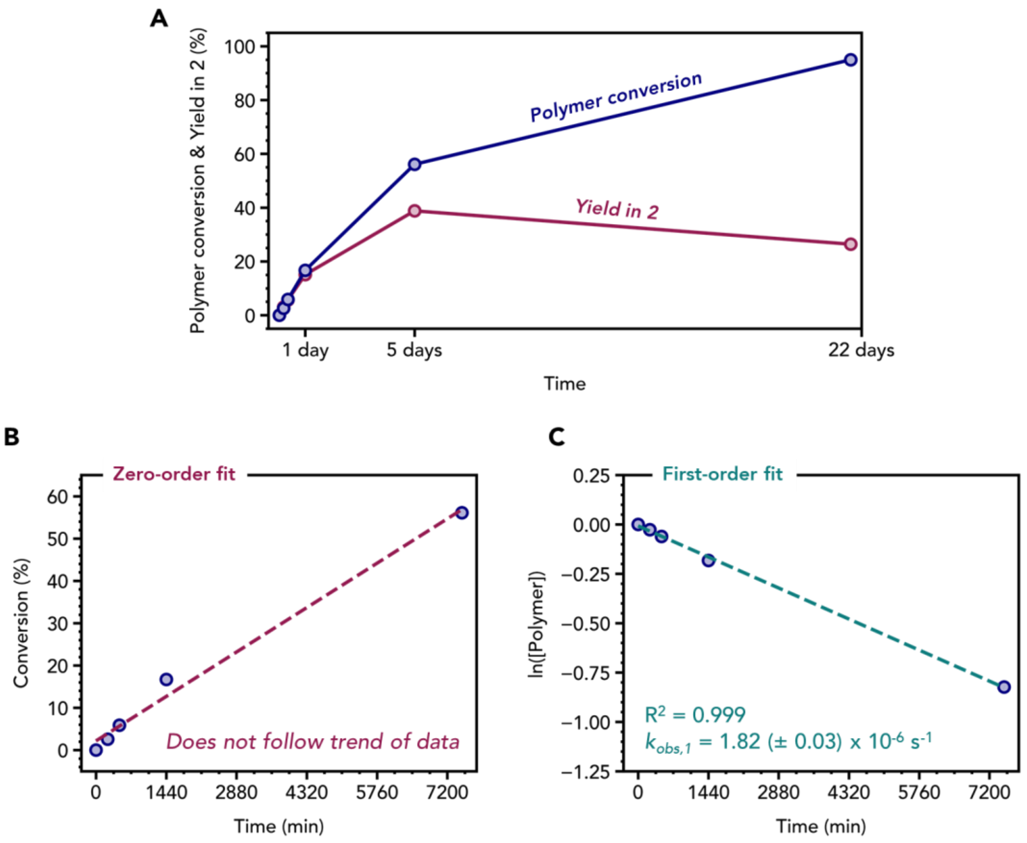

Figure S 78 – Plots used to monitor the kinetics of degradation of **P1cd**: (A Complete plot of the polymer linkage conversion and yield in **2** *vs* time for the degradation of **P1cd**. (B) Plot of the polymer linkage conversion *vs* time until half conversion. A zero-order does not describe well the data. (C) Plot of ln([Polymer]) *vs* time over a broader timescale. A first-order dependence on polymer concentration was observed and a rate constant k_{first} could be extracted from a linear fit.

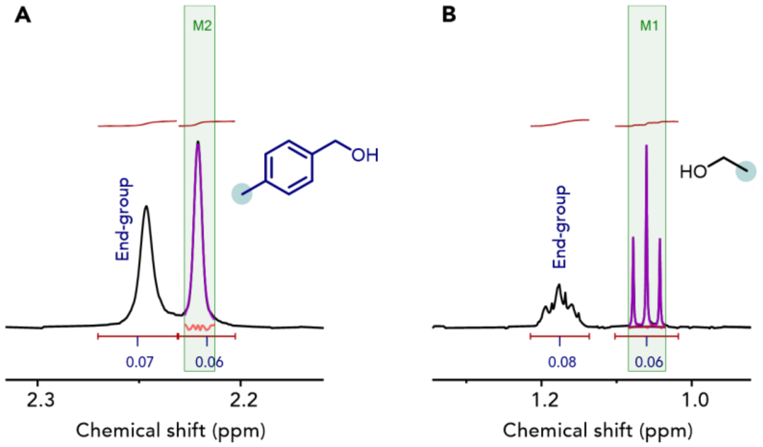

Figure S 79 – Zoomed ^{1}H-NMR spectra (400 MHz, D_2O) of the crude reaction medium for the degradation of **P1cd** at pH 8 after 5 days. (A) Both end groups and free MBA are observed. (B) Both end groups and free ethanol are observed. Integration proves that both small molecules are released at an identical loading.

References

(1) Deacy, A. C.; Moreby, E.; Phanopoulos, A.; Williams, C. K. Co(III)/Alkali-Metal(I) Heterodinuclear Catalysts for the Ring-Opening Copolymerization of CO(2) and Propylene Oxide. *J Am Chem Soc* **2020**, *142* (45), 19150–19160. DOI: 10.1021/jacs.0c07980.

(2) d'Arcy, R.; El Mohtadi, F.; Francini, N.; DeJulius, C. R.; Back, H.; Gennari, A.; Geven, M.; Lopez-Cavestany, M.; Turhan, Z. Y.; Yu, F.; Lee, J. B.; King, M. R.; Kagan, L.; Duvall, C. L.; Tirelli, N. A Reactive Oxygen Species-Scavenging 'Stealth' Polymer, Poly(thioglycidyl glycerol), Outperforms Poly(ethylene glycol) in Protein Conjugates and Nanocarriers and Enhances Protein Stability to Environmental and Biological Stressors. *Journal of the American Chemical Society* **2022**, *144* (46), 21304–21317. DOI: 10.1021/jacs.2c09232.
